# Consecutive HDDA and TDDA reactions of silicon-tethered tetraynes for the synthesis of dibenzosilole-fused polycyclic compounds and their unique reactivity†
†Electronic supplementary information (ESI) available. CCDC 1826048, 1826049, 1890968 and 1830394. For ESI and crystallographic data in CIF or other electronic format see DOI: 10.1039/c9sc00960d


**DOI:** 10.1039/c9sc00960d

**Published:** 2019-05-30

**Authors:** Akihito Mitake, Rikako Nagai, Ayato Sekine, Hideaki Takano, Natsuhiko Sugimura, Kyalo Stephen Kanyiva, Takanori Shibata

**Affiliations:** a Department of Chemistry and Biochemistry , School of Advanced Science and Engineering , Waseda University , Shinjuku , Tokyo 169-8555 , Japan . Email: tshibata@waseda.jp ; Fax: +81-3-5286-8098; b Materials Characterization Central Laboratory , School of Advanced Science and Engineering , Waseda University , Shinjuku , Tokyo 169-8555 , Japan; c Global Center for Science and Engineering , School of Advanced Science and Engineering , Waseda University , Shinjuku , Tokyo 169-8555 , Japan

## Abstract

Silicon-tethered tetraynes possessing a 1,3-diyne moiety underwent consecutive hexadehydro- and tetradehydro-Diels–Alder reactions to give a series of fused polycyclic aromatic compounds containing a dibenzosilole skeleton. The benzene ring in the product acted as a 1,3-diene and reacted with the active alkyne as well as oxygen to provide [4 + 2] cycloadducts.

## Abstract 2

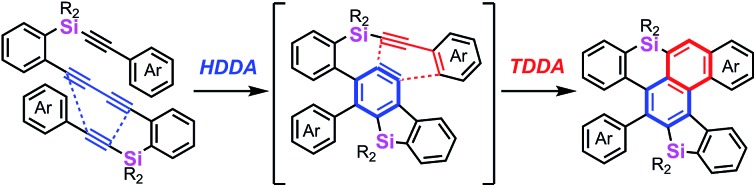
Silicon-tethered tetraynes with a 1,3-diyne moiety underwent consecutive hexadehydro- and tetradehydro-Diels–Alder reactions to give fused polycyclic aromatic compounds.

## 

While the Diels–Alder reaction is a [4 + 2] cycloaddition of a 1,3-diene and an alkene, the dehydro-Diels–Alder (DDA) reaction involves an alkyne moiety(ies) in the substrate(s).^1^ For example, the tetradehydro-Diels–Alder (TDDA) reaction of a 1,3-enyne and an alkyne gives a substituted benzene ring with perfect atom-economy *via* a strained cyclic allene intermediate along with a 1,5-hydrogen shift. In particular, the intramolecular TDDA reaction of arylalkynes, where part of the arene acts as an ene moiety, is attractive, because fused polycyclic aromatic compounds can be prepared in one pot. Saá is a pioneer in the synthetic use of TDDA and comprehensively studied an intramolecular reaction of diarylacetylene and alkyne;^2^ the reaction of ynamides gave carbazole derivatives (Scheme 1a).^3^ Our group also reported an intramolecular TDDA reaction for the synthesis of binaphthyl compounds.^4^ Recently, we developed the consecutive intramolecular TDDA reaction of sulfur-tethered tetraynes for the preparation of axially chiral bis(benzothiophene) derivatives and further upgraded this transformation to an enantioselective synthesis by a chiral metal-catalyzed reaction (Scheme 1b).^5^

**Scheme 1:**
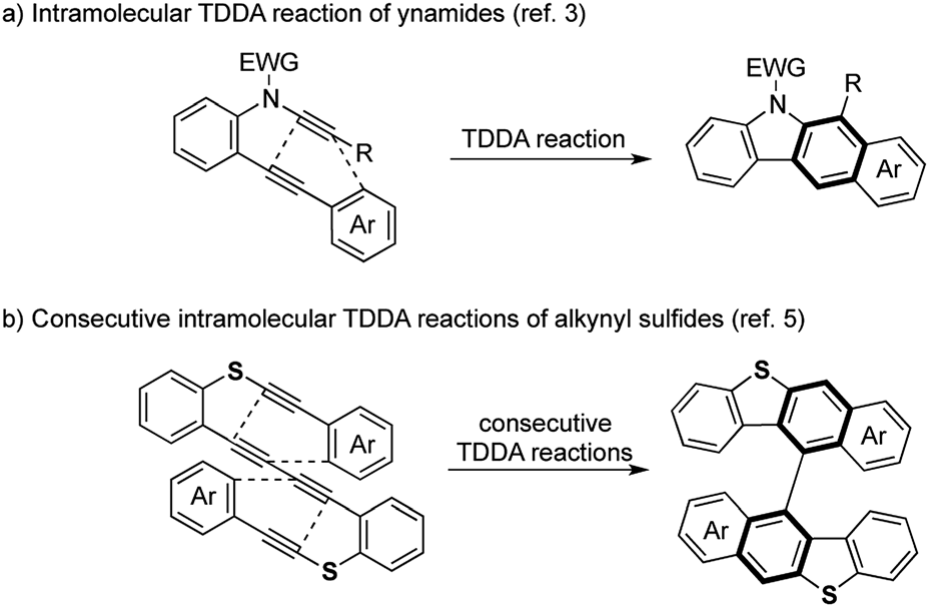
Intramolecular TDDA reactions for the synthesis of dibenzoheteroles.

Against this background, we next examined the thermal reaction using silicon analogue **1a**^6^ in hot toluene for the preparation of an axially chiral bis(benzosilole) derivative. Unexpectedly, we obtained dibenzosilole-fused heptacyclic compound **2a**, the structure of which was finally decided upon based on X-ray analysis (Scheme 2). We considered that consecutive intramolecular DDA reactions gave polycyclic compounds. The first step is a hexadehydro-Diels–Alder (HDDA) reaction of 1,3-diyne and alkyne to give benzosilole-fused benzyne and the second step is a TDDA reaction with the remaining arylalkyne moiety. Since the first report of the HDDA reaction,7*a* Hoye reported various reagents for trapping of the reactive benzyne intermediates.7b–v Recently, consecutive HDDA reactions were developed for the one-pot synthesis of fused polycyclic compounds.7*w* In contrast, we demonstrate here the first example of consecutive HDDA and TDDA reactions as well as a HDDA reaction along with [2 + 2 + 2 + 2] cycloaddition. We further discuss the unique reactivity of the benzene moiety of the silicon-containing polycyclic compound.

**Scheme 2:**
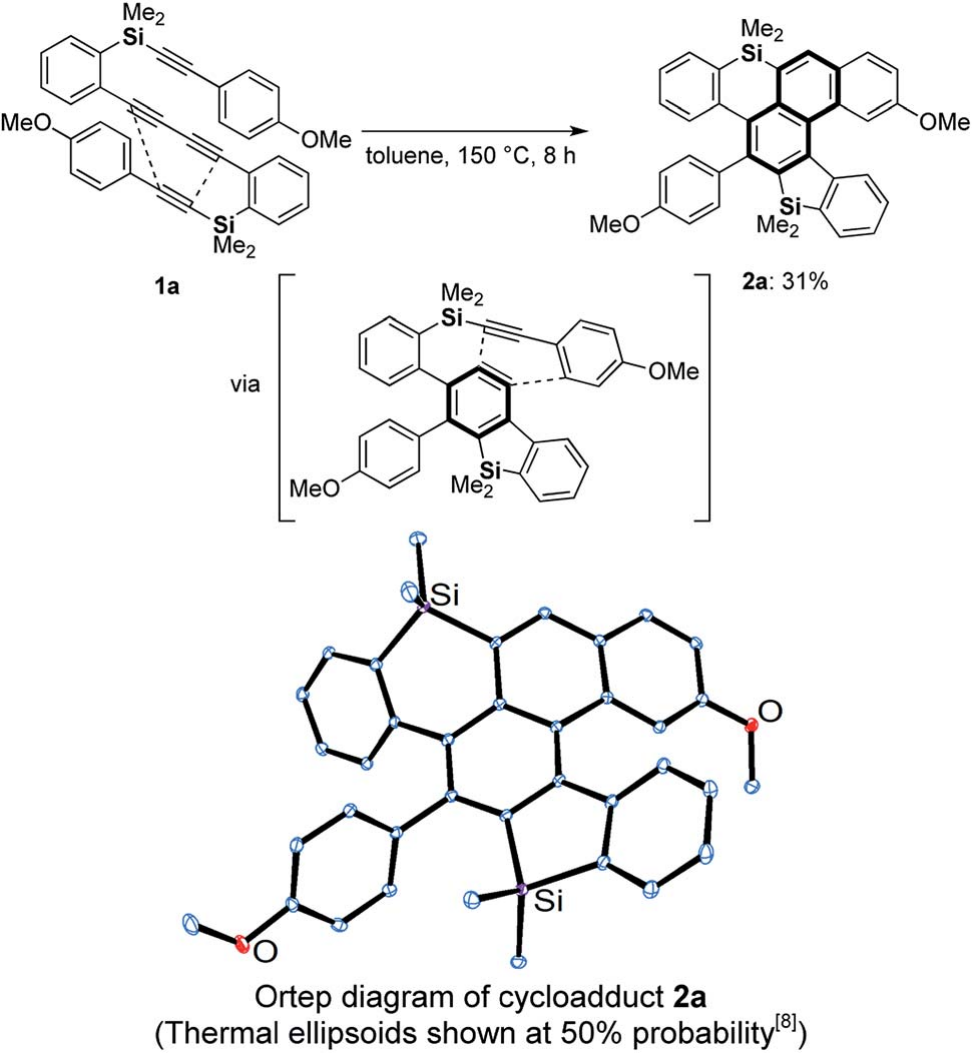
Consecutive HDDA and TDDA reactions of silicon-tethered tetrayne **1a** possessing a 1,3-diyne moiety.

We chose 1,4-bis(2-(dimethyl-(2-(4-methoxyphenyl)ethynyl)-silyl)phenyl)buta-1,3-diyne (**1a**) as a model substrate and screened the thermal conditions for the consecutive HDDA and TDDA reactions (Table 1). When dibutyl ether was used, tetrayne **1a** was completely consumed within 8 h and dibenzosilole-fused cycloadduct **2a** was obtained in moderate NMR yield (entry 1). While benzonitrile realized a yield comparable to that with the etherate solvent, propionitrile gave the best yield of 72% with a longer reaction time (entries 2 and 3). After we investigated the concentration effect, the yield was improved to 83% under dilute conditions, but with a prolonged reaction time (entries 3–5). Tetrayne **1a** was completely consumed within 1 h under microwave irradiation, but the reaction became messy, and the yield of **2a** was low (entry 6). We determined that entry 5 represented the best conditions.

**Table 1:** Screening of thermal conditions for the consecutive DDA reactions^*a*^

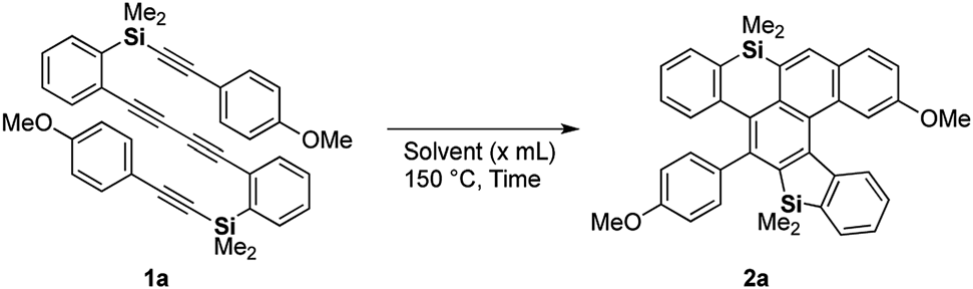			
Entry	Solvent (*x* mL)	Time (h)	NMR yield^*b*^ (%)
1	Dibutyl ether (5)	8	56
2	PhCN (5)	4	52
3	CH_3_CH_2_CN (5)	48	72
4	CH_3_CH_2_CN (1.7)	48	41
5	CH_3_CH_2_CN (15)	48	83
6^*c*^	CH_3_CH_2_CN (3)	1	27

^*a*^The reaction was conducted on a 0.05 mmol scale.

^*b*^Yields were determined by NMR using 1,1,2,2-tetrachloroethane as an internal standard.

^*c*^The reaction was conducted on a 0.03 mmol scale under microwave irradiation.

We next examined the substrate scope of aryl groups on the alkyne termini in propionitrile at 150 °C (Table 2). Compound **2a** was obtained in 81% isolated yield. Phenyl- and 4-fluorophenyl-substituted tetraynes **1b** and **1c** were transformed into the corresponding heptacyclic compounds **2b** and **2c** in moderate yields. Electron-rich and *ortho*-substituted arenes could also be used and cycloadducts **2d** and **2e**^9^ were obtained in moderate yields. In the case of 4-biphenyl-substituted product **2f**, the yield was low because it was difficult to isolate due to its high crystallinity. The reactions of 1-naphthyl and 2-benzothiophenyl-substituted tetraynes **1g** and **1h** also proceeded to give octacyclic cycloadduct **2g**^9^ and dihetero[6]helicene **2h** consisting of dibenzothiophene and dibenzosilole, respectively.
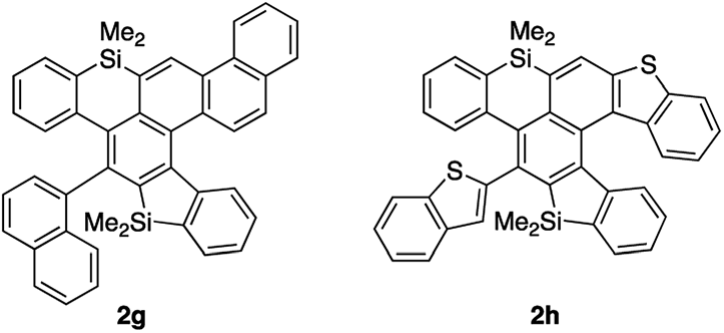


**Table 2:** Substrate scope of tetrayne **1a**^*a*^

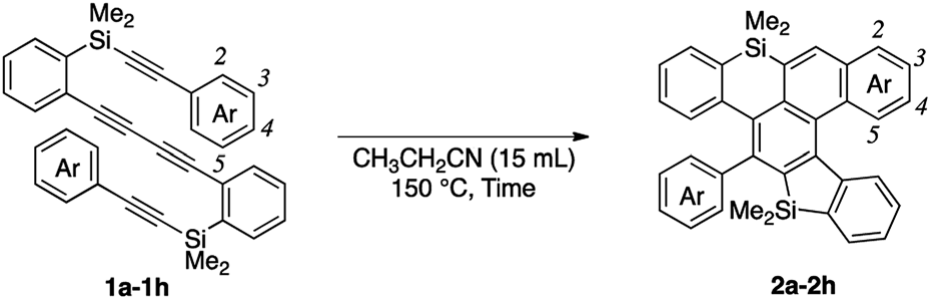			
Entry	Ar	Time (h)	Yield (%)
1	4-MeOC_6_H_4_	40	81 (**2a**)
2	C_6_H_5_	48	55 (**2b**)
3	4-FC_6_H_4_	48	51 (**2c**)
4	3,5-(MeO)_2_C_6_H_3_	24	56 (**2d**)
5	2-MeOC_6_H_4_	40	52 (**2e**)
6	4-PhC_6_H_4_	24	43 (53)^*b*^ (**2f**)
7	1-Naphthyl	24	53 (**2g**)
8	2-Benzothienyl	24	51 (**2h**)

^*a*^Tetrayne (0.05 mmol) and propionitrile (15 ml) were used and the product was purified by preparative TLC.

^*b*^Yield in parenthesis was determined by NMR using 1,1,2,2-tetrachloroethane as an internal standard.

While the reaction of 2-naphthyl-substituted tetrayne **1i** possibly affords two regioisomers **2i** and **2i′**, sila[6]helicene **2i** was the only cycloadduct detected, probably due to the higher reactivity of the α-position of the naphthyl group (Scheme 3). Diphenylsilyl-tethered tetrayne **1j** was also transformed into the corresponding cycloadduct **2j** (Scheme 4).

**Scheme 3:**
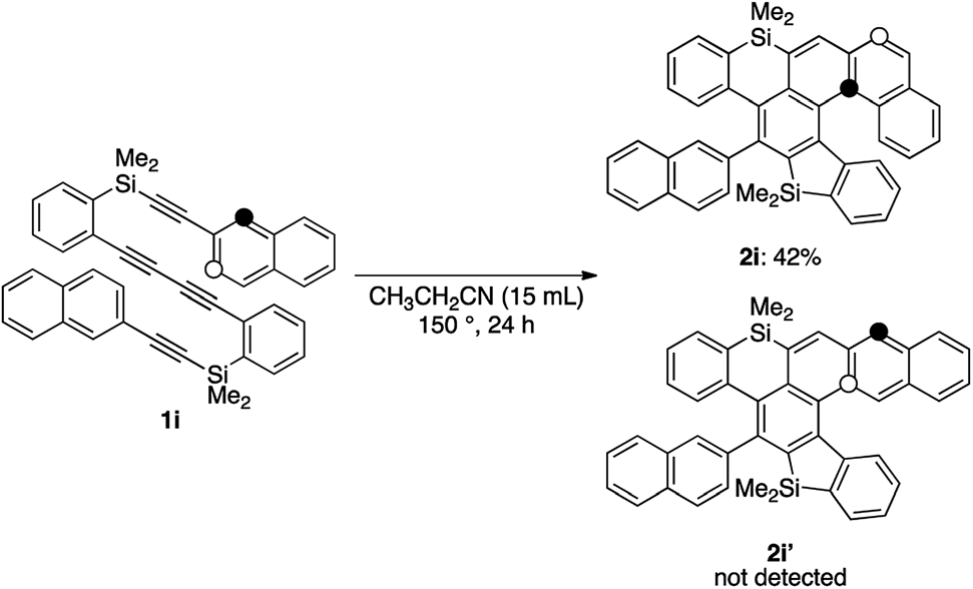
Regioselective reaction of tetrayne **1i**.

**Scheme 4:**
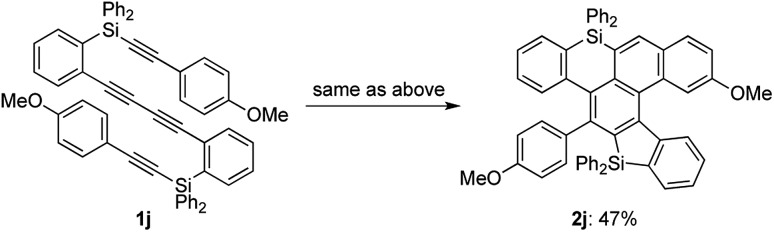
Reaction of diphenylsilyl-tethered tetrayne **1j**.

We conducted mechanism study in order to figure out why silicon-tethered tetraynes underwent the HDDA reaction, not the TDDA reaction. As shown by the results of DFT calculations using triyne **A** as a model substrate, the activation energy of both HDDA and TDDA reactions *via* diradical intermediates7*k* was large (54.3 and 49.0 kcal mol^–1^, respectively). In contrast, the concerted pathway showed smaller activation energy; moreover, the concerted HDDA reaction (27.1 kcal mol^–1^) was clearly more favorable than the concerted TDDA reaction (35.6 kcal mol^–1^) (Fig. 1).^10^

**Fig. 1:**
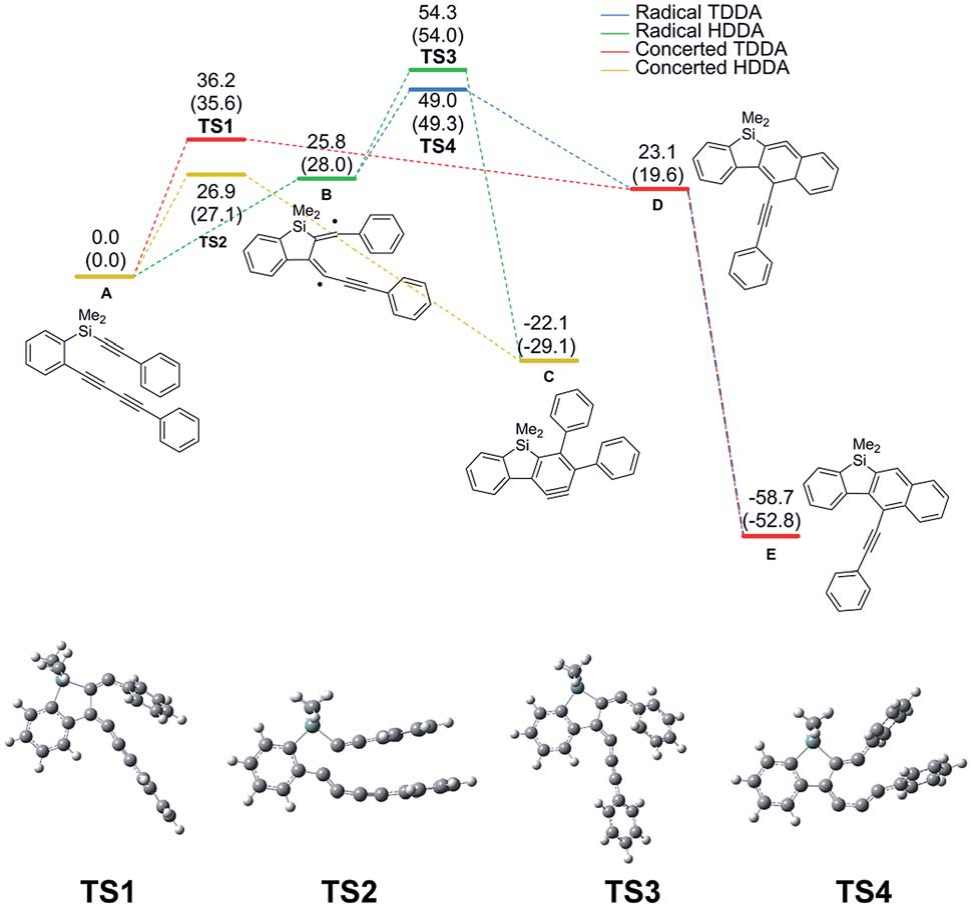
Relative Gibbs free energy (Δ*G*) diagram of Si-tethered triyne **A** at 423.15 K (kcal mol^–1^). Relative electronic energy (Δ*E*) is in parentheses.

In all of the entries above, some by-products were always formed. Among them, the major isolated by-products were cyclooctatetraene derivatives. The structure of the by-product in the reaction of **1a** was finally determined to be saddle-shaped compound **3a** by X-ray crystallographic analysis (Scheme 5 below). We considered the mechanism to be dimerization of *in situ*-generated benzosilole-fused benzyne (Scheme 5 above). To the best of our knowledge, this is the first example of the thermal [2 + 2 + 2 + 2] cycloaddition of alkynes for the construction of an eight-membered ring system.^11^ Therefore, we further examined the reaction conditions.^12^ As a result, **3b** was obtained in moderate yield as a major product under more concentrated conditions in chlorobenzene (Scheme 6).^13^

**Fig. 2:**
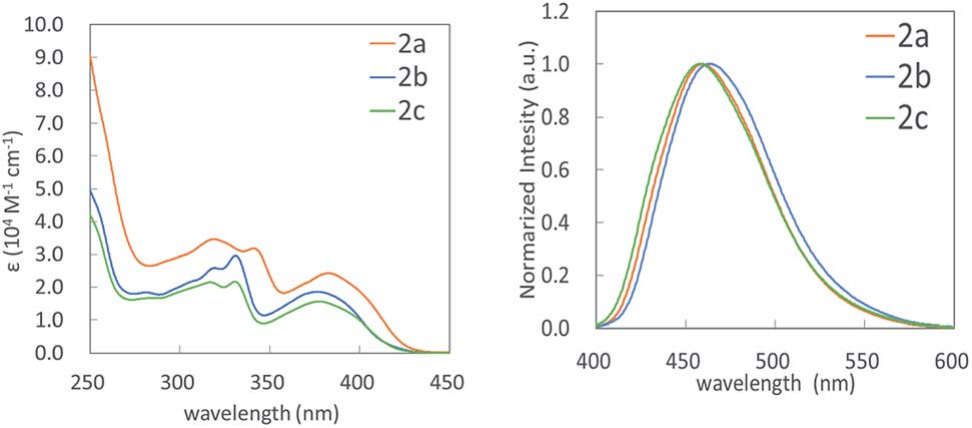
UV-vis (left) and fluorescence spectra (right) of **2a–2c**.

**Scheme 5:**
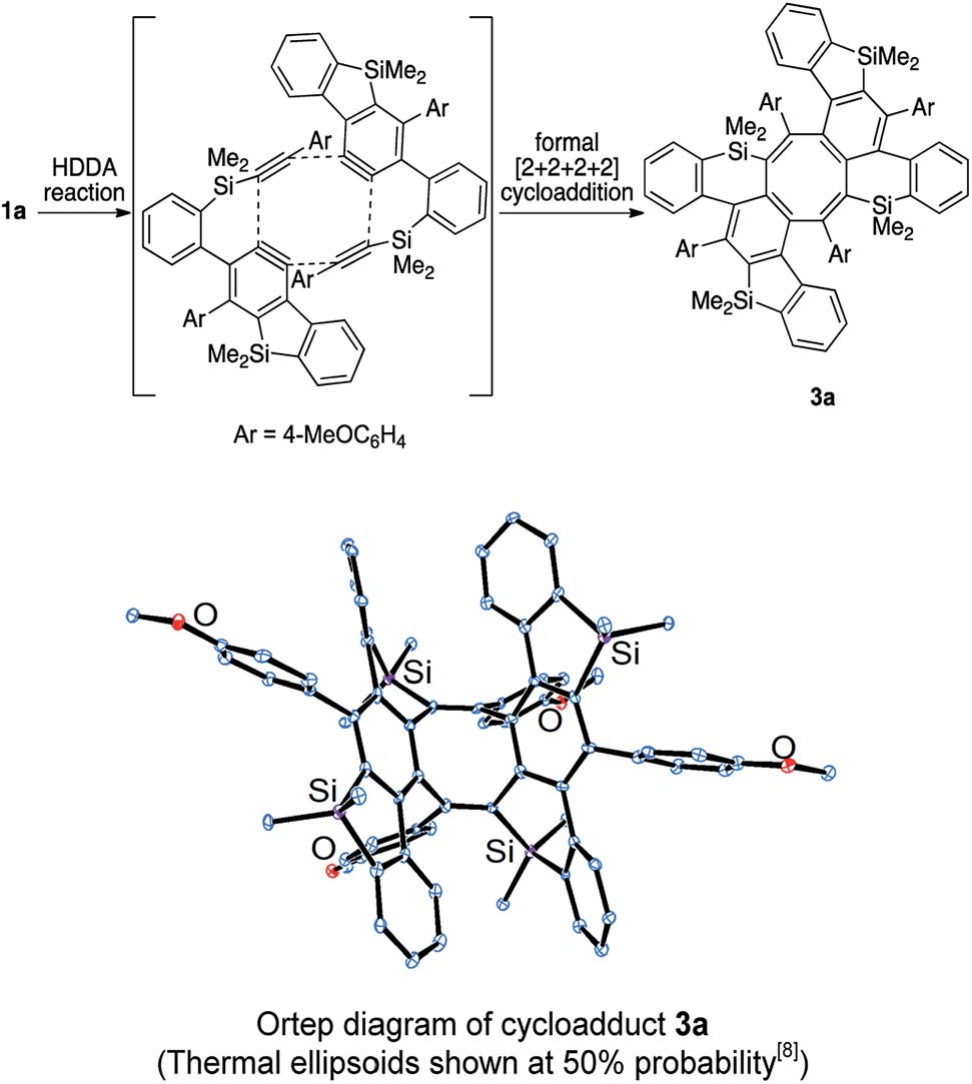
Proposed mechanism for the formation of saddle-shaped compound **3a**.

**Scheme 6:**
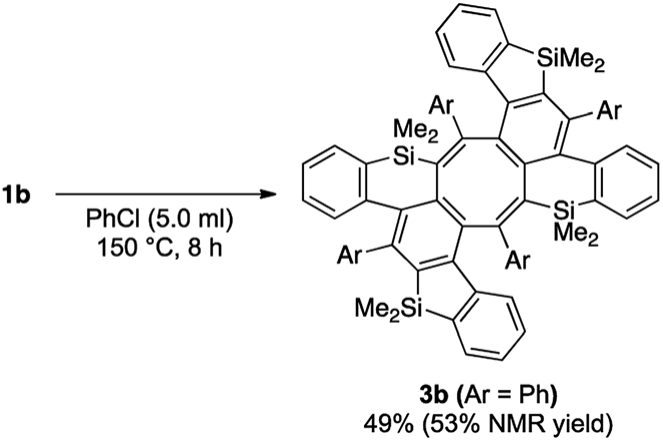
Formal [2 + 2 + 2 + 2] cycloaddition of two benzynes and two alkynes.

We measured UV-vis and fluorescence spectra of benzosilole-fused polycyclic compounds **2a–2c** in dichloromethane as well as their quantum yields in both dichloromethane solution and the solid state (Fig. 2 and Table 3). These compounds are fluorescent and their quantum yields in the solid state were higher than those in solution. Substitution at the 6- and 15-positions did not affect their photophysical properties. The Stokes shifts were twice as much as those for simple sila[5]helicene,^14^ probably due to the fused siline ring. The torsion angle of the silahelicene moiety of **2a** was 27.0°, which was significantly larger than that of the simple sila[5]helicene (17.6°).^14^

**Table 3:** Photophysical properties of **2a–2c**^*a*^

Comp.	*λ*_max(abs)_ ^*b*^ [nm], (*ε* [×10^4^ cm^–1^ M^–1^])	*λ*_max(emi)_ ^*c*^ ^,^ ^*d*^ [nm]	*Φ* ^*c*^ ^,^ ^*d*^ (solution, solid)
**2a**	319 (3.5), 341 (3.2), 383 (2.4)	459	0.15, 0.22
**2b**	319 (2.6), 331 (3.0), 376 (1.9)	463	0.14, 0.21
**2c**	317 (2.1), 331 (2.2), 377 (1.6)	458	0.14, 0.16

^*a*^UV-vis and fluorescence spectra of **2** were measured in CH_2_Cl_2_.

^*b*^ **2a** 1.7 × 10^–5^ M; **2b** 2.1 × 10^–5^ M; **2c** 3.0 × 10^–5^ M.

^*c*^ **2a** 1.7 × 10^–6^ M; **2b** 2.1 × 10^–6^ M; **2c** 3.0 × 10^–6^ M.

^*d*^Excitation wavelength: **2a** 383 nm; **2b** 376 nm; **2c** 377 nm.

We further investigated the reactivity of the unique π-system containing silicon atoms. We first conducted the reaction of silahelicene **2a** with excess amounts of *in situ*-generated benzyne as a reactive alkyne; consecutive [4 + 2] cycloaddition proceeded at 30 °C for 3 h to give polycyclic compound **4** possessing two bridged systems, the structure of which was confirmed by X-ray crystallographic analysis (Scheme 7).^15^ Even when the amount of the benzyne precursor was decreased, a 1 : 1 cycloadduct could not be detected.

**Scheme 7:**
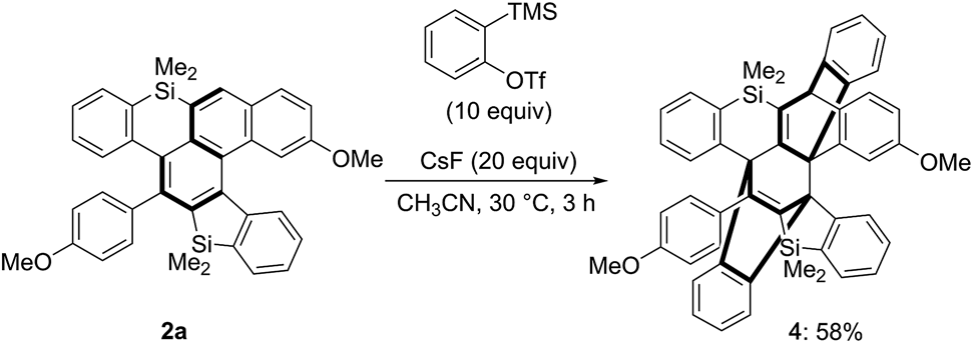
Consecutive [4 + 2] cycloadditions of silahelicene **2a** with benzyne.

While the [4 + 2] cycloaddition of **2a** with dimethyl acetylenedicarboxylate (DMAD) required a high reaction temperature as well as a long reaction time, 1 : 1 cycloadduct **5** was the only detectable product (Scheme 8 above).^16^ Interestingly, the reaction of **2a** proceeded under an atmospheric pressure of oxygen at 50 °C to give cyclic peroxide **6** in moderate yield, the structure of which was also confirmed by X-ray crystallographic analysis (Scheme 8 below).^17^

**Scheme 8:**
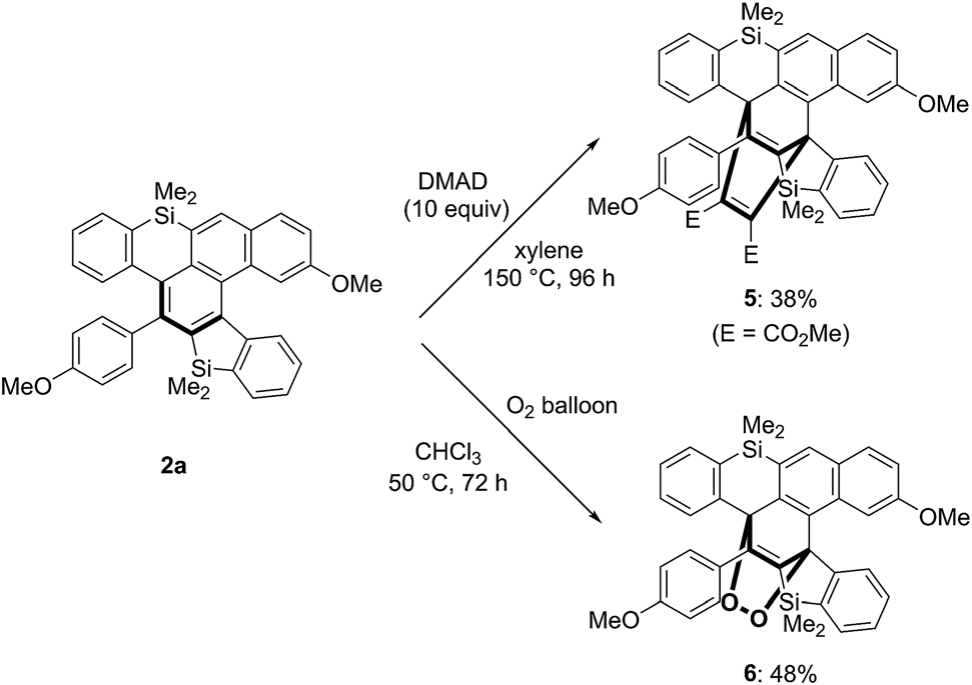
Diels–Alder reactions of silahelicene **2a** with DMAD and oxygen.

To explain the high reactivity with oxygen, we calculated the values of NICS(0) for cycloadduct **2a** and cyclic peroxide **6** (Table 4).^18^ The aromaticities of rings c and e of **2a** were relatively weak, because they are fused with the antiaromatic siline (ring b). Ring c gained aromaticity by [4 + 2] cycloaddition with oxygen (–3.30 to –7.22).

**Table 4:** NICS(0) of sila[5]helicene **2a** and **6**^*a*^

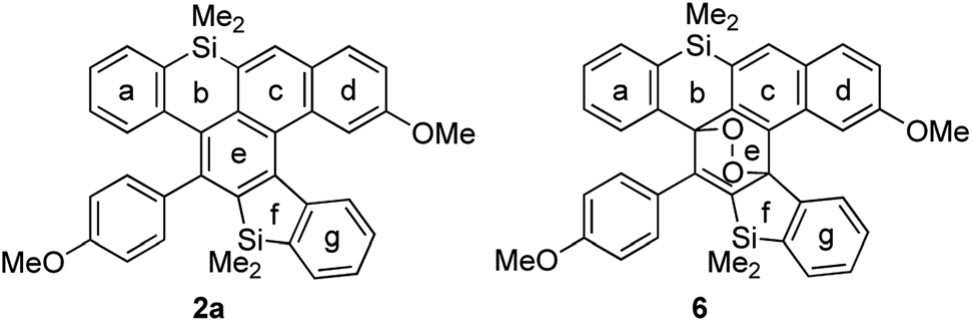		
Ring	**2a**	**6**
a	–6.09	–6.89
b	4.50	3.68
c	–3.30	–7.22
d	–8.27	–7.75
e	–5.03	2.46
f	4.39	2.25
g	–4.85	–6.22

^*a*^GIAO B3LYP/6-31+G(d,p).

In conclusion, we have developed the first example of consecutive HDDA and TDDA reactions using silicon-tethered tetraynes possessing a 1,3-diyne moiety *via* benzosilole-fused benzynes. The obtained silicon-containing polycyclic aromatic compound acted as a diene and underwent [4 + 2] cycloaddition with active alkynes. Notably, it could react with non-activated oxygen to give a cyclic peroxide.

## Conflicts of interest

There are no conflicts to declare.

## Supplementary Material

Supplementary information

Crystal structure data
