# Supplementary material for: Consecutive HDDA and TDDA reactions of silicon-tethered tetraynes for the synthesis of dibenzosilole-fused polycyclic compounds and their unique reactivity
Source: Chem Sci. 2019 May 30;10(27):6715–20. doi: 10.1039/c9sc00960d (PMC6625486; doi:10.1039/c9sc00960d)
Supplement: Supplementary file 1 [file SC-010-C9SC00960D-s001.pdf]

Electronic Supplementary Material (ESI) for Chemical Science.
This journal is © The Royal Society of Chemistry 2019

Supporting Information

Table of contents

i.	Experimental general information	S2
ii.	General procedure for consecutive intramolecular DDA under thermal condition	S3
iii.	Synthetic transformation from sila[5]helicene	S3
iv.	Reaction of carbon analogue tetrayne S1	S3
v.	Condition screening for the synthesis of 8-memberd ring system	S3
vi.	Characterization data for new compounds	S4
vii.	DFT calculations	S13
viii.	References	S27
ix.	Copies of ^{1}H and ^{13}C NMR spectra for new compounds	S29

i. Experimental General Information

^{1}H NMR spectra were recorded on JEOL ECX-500 (500 MHz) spectrometers. The chemical shifts were reported in parts per million (δ) relative to internal standard TMS (0 ppm) for $CDCl_3$. The peak patterns are indicated as follows: s, singlet; d, doublet; dd, doublet of doublet; m, multiplet. The coupling constants, *J*, are reported in Hertz (Hz). ^{13}C NMR spectra were obtained by JEOL ECX-500 (125 MHz) spectrometers and referenced to the internal solvent signals (central peak is 77.16 ppm in $CDCl_3$). $CDCl_3$ was used as an NMR solvent. High-resolution mass spectra (HRMS) were measured on an ESI (Electro Spray Ionization) – Orbitrap mass spectrometer method or positive mode DART.[1] Ultraviolet spectrum was measured on a JASCO V-630 spectrometer. Fluorescence spectrum was measured on a JASCO FP-8200 spectrofluorometer. X-ray structures were obtained by a Rigaku R-AXIS RAPID diffractometer. Preparative thin-layer chromatography (PTLC) was performed with silica gel-precoated glass plates (Merck 60 GF254) prepared in our laboratory, Flash column chromatography was performed over silica gel 200-300. All reagents were weighed and handled in air and backfilled under argon at room temperature. Unless otherwise noted, all reactions were performed under an argon atmosphere. All reagents were purchased from Wako, Kanto, Aldrich and TCI and used without further purification.

Preparation of tetraynes

Tetraynes **1a-1i** were prepared by the literature procedure starting from 1,4-bis(2-bromophenyl)buta-1,3-diyne (**S1**).[2] A THF solution of disilylated product **S2** were *in situ* prepared by the dilithiation of **S2** along with quenching with dimethoxydimethylsilane. Then a THF solution of arylethynyllithium, which was prepared from the corresponding terminal alkyne in another pot, was transferred by cannula. Tetraynes **1a-1c** were already reported.[2]

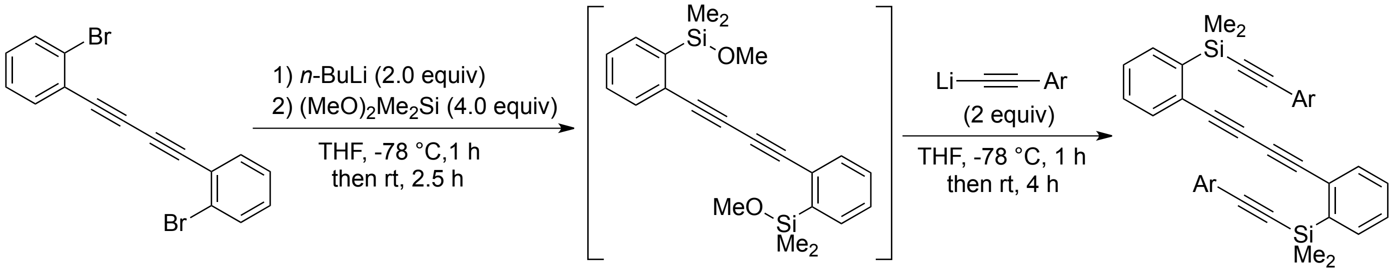

S1 **S2** **1a-1i**

ii. General procedure for consecutive intramolecular DDA under thermal condition

Tetrayne (0.05 mmol) was placed in a sealed tube, which was then backfilled with argon (x3). To the reaction vessel was added propionitrile (15.0 mL). Then the solution was heated at 150 °C for 24-48 h. The reaction mixture was cooled to room temperature and the solvent was evaporated to dryness. The obtained crude products were purified by preparative TLC.

iii. Synthetic transformation from sila[5]helicene

Synthetic transformation into 4

Cycloadduct **2a** (0.025 mmol) and cesium fluoride (20 equiv) were placed in a sealed tube, which was then backfilled with argon (x3). To a reaction vessel was added 2-(trimethylsilyl)pheny trifluoromethanesulfonate

(10 equiv) and acetonitrile (0.2 mL). Then, the solution was stirred at 30 °C for 3 h. The reaction mixture was quenched with H_2O. The aqueous layer was extracted with dichloromethane and the organic layer was dried over Na_2SO_4. The dry organic extract was filtered, and evaporated under reduced pressure. After removal of solvent, the crude products were purified by preparative TLC.

Synthetic transformation into 5

Cycloadduct **2a** (0.025 mmol) was placed in a sealed tube, which was then backfilled with argon (x3). To a reaction vessel was added dimethylacetylenedicarboxylate (10 equiv) and xylene (0.2 mL). Then, the solution was stirred at 150 °C for 96 h. The reaction mixture was cooled to room temperature and evaporated to dryness. The obtained crude products were purified by preparative TLC.

Synthetic transformation into 6

Cycloadduct **2a** (0.025 mmol) was placed in a schlenk tube, which was then backfilled with O_2. To a reaction vessel was added chloroform (0.25 mL). Then, the solution was stirred at 50 °C for 72 h. The reaction mixture was cooled to room temperature and evaporated to dryness. The obtained crude products were purified by preparative TLC.

iv. Reaction of carbon analogue tetrayne S1

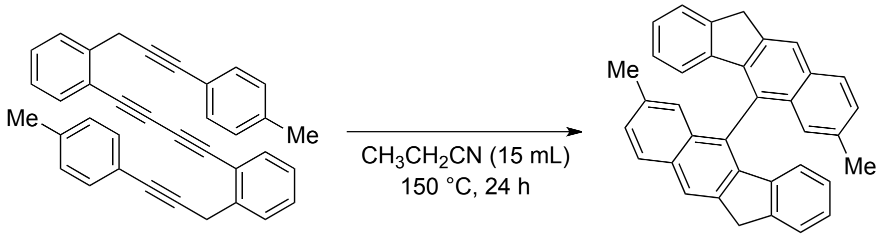

S1 **S2:** 57%

v. Condition screening for the synthesis of 8-memberd ring system

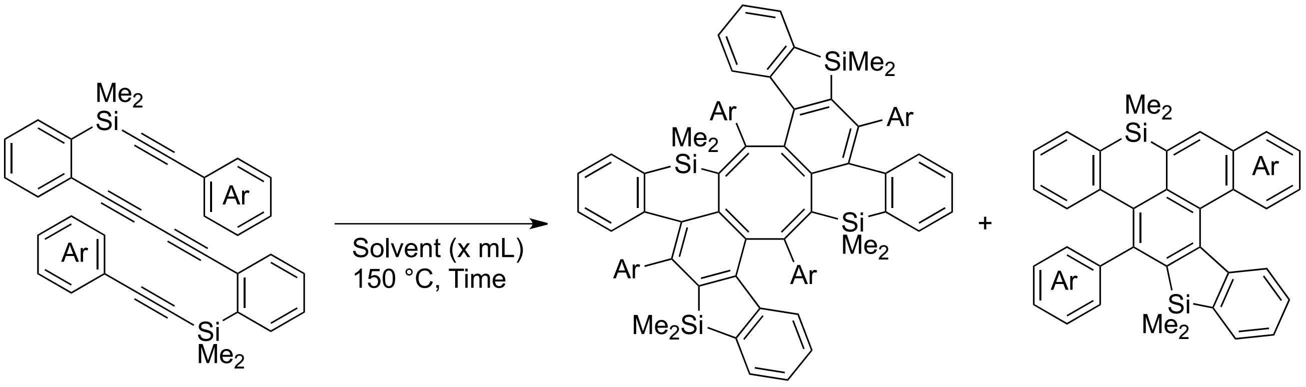

1 **3** **2**

Comp.	Solvent	Solvent (x)	Time / h	NMR Yield of **3** / %	NMR Yield of **2** / %
**1a**	Dibutyl ether	0.25	24	24	18
↑	PhCN	↑	↑	31	40
↑	PhCl	↑	↑	32	13
**1b**	↑	↑	↑	43	11
↑	↑	1.7	6	39	17
↑	↑	5	8	53	21

vi. Characterization data for new compounds

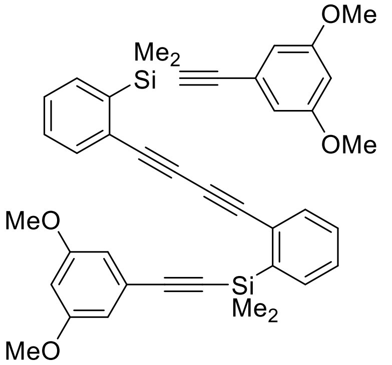

1,4-Bis(2-(((3,5-dimethoxyphenyl)ethynyl)dimethylsilyl)phenyl)buta-1,3-diyne (1d). Isolated by column chromatography on silica gel (hexane/ethyl acetate = 20/1 to 5/1). The title compound was obtained as a brown solid (38%). Mp 40-41 °C; ^{1}H NMR δ 7.86-7.91 (m, 2H), 7.53-7.57 (m, 2H), 7.34-7.42 (m, 4H), 6.68-6.71 (m, 4H), 6.43-6.46 (m, 2H), 3.75 (s, 12H), 0.63 (s, 12H); ^{13}C NMR δ 160.6, 140.8, 135.3, 133.5, 129.5, 128.6, 126.8, 124.3, 109.9, 107.5, 102.5, 91.5, 83.5, 77.7, 55.5, -0.7; HRMS (ESI, positive) calcd. for $C_{40}H_{38}NaO_4Si_2$ $[M+Na]^+$: 661.2201; found: 661.2197.

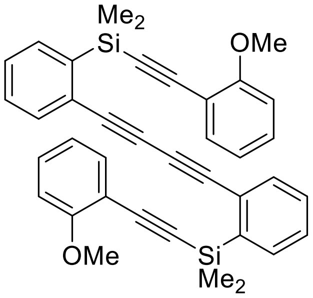

1,4-Bis(2-(((2-methoxyphenyl)ethynyl)dimethylsilyl)phenyl)buta-1,3-diyne (1e). Isolated by column chromatography on silica gel (hexane/ethyl acetate = 20/1 to 5/1). The title compound was obtained as a brown solid (32%). Mp 56-57 °C; ^{1}H NMR δ 8.10-8.15 (m, 2H), 7.54-7.61 (m, 4H), 7.36-7.46 (m, 4H), 7.29-7.35 (m, 2H), 6.86-6.95 (m, 4H), 3.92 (s, 6H), 0.66 (s, 12H); ^{13}C NMR δ 160.3, 140.8, 135.4, 133.8, 132.9, 129.9, 128.9, 128.1, 126.3, 120.0, 111.9, 110.4, 103.5, 95.9, 83.1, 55.5, -1.0 (one of alkynyl peaks is overlapped with a chloroform peak); HRMS (ESI, positive) calcd. for $C_{38}H_{34}NaO_2Si_2$ $[M+Na]^+$: 601.1990; found: 601.1988.

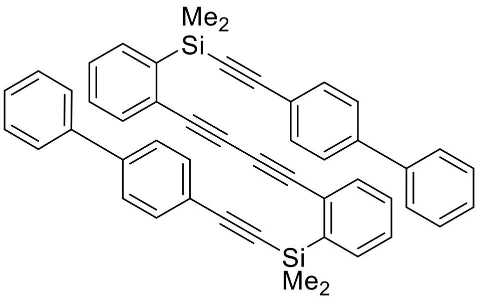

1,4-Bis(2-(([1,1'-biphenyl]-4-ylethynyl)dimethylsilyl)phenyl)buta-1,3-diyne (1f). Isolated by recrystallization (dichloromethane). The title compound was obtained as a white solid (43%). Mp 177-178 °C; ^{1}H NMR δ 7.91-7.94 (m, 2H), 7.52-7.65 (m, 14H), 7.34-7.47(m, 10H), 0.66 (s, 12H); ^{13}C NMR δ 141.4, 140.8,

140.3, 135.3, 133.4, 132.6, 129.4, 128.8, 128.5, 127.7, 127.0, 126.9, 126.7, 121.9, 107.3, 92.6, 83.4, 77.5, -0.8; HRMS (ESI, positive) calcd. for $C_{48}H_{38}NaSi_2$ $[M+Na]^+$: 693.2404; found: 693.2399.

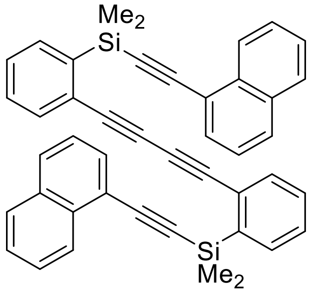

1,4-Bis(2-(dimethyl(naphthalen-1-ylethynyl)silyl)phenyl)buta-1,3-diyne (1g). Isolated by preparative TLC (hexane/ethyl acetate = 5/1). The title compound was obtained as brown oil (42%). ^{1}H NMR δ 8.41-8.47 (m, 2H), 7.96-8.02 (m, 2H), 7.75-7.80 (m, 6H), 7.50-7.56 (m, 4H), 7.43-7.49 (m, 2H), 7.30-7.40 (m, 6H), 0.74 (s, 12H); ^{13}C NMR δ 141.4, 136.0, 134.1, 134.1, 133.7, 131.9, 130.1, 129.9, 129.3, 128.9, 127.7, 127.4, 127.1, 127.0, 125.8, 121.3, 106.2, 97.7, 84.2, 78.3, 0.1; HRMS (ESI, positive) calcd. for $C_{44}H_{34}Si_2$ $[M]^+$: 618.2194; found: 618.2182.

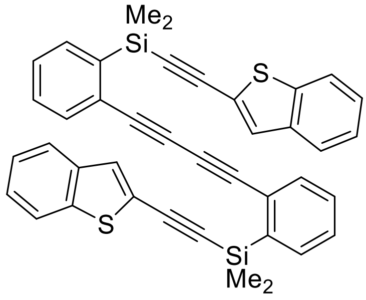

1,4-Bis(2-((benzo[*b*]thiophen-2-ylethynyl)dimethylsilyl)phenyl)buta-1,3-diyne (1h). Isolated by column chromatography on silica gel (hexane/ethyl acetate = 10/1). The title compound was obtained as a yellow solid (16%). Mp 142-143 °C; ^{1}H NMR δ 7.86-7.89 (m, 2H), 7.73-7.77 (m, 2H), 7.69-7.72 (m, 2H), 7.54-7.58 (m, 4H), 7.32-7.44 (m, 8H), 0.67 (s, 12H); ^{13}C NMR δ 140.3, 140.2, 138.8, 135.2, 133.4, 130.0, 129.5, 128.6, 126.7, 125.6, 124.7, 123.9, 122.9, 122.0, 100.0, 98.6, 83.3, 77.6, -0.9; HRMS (ESI, positive) calcd. for $C_{40}H_{30}NaS_2Si_2$ $[M+Na]^+$: 653.1220; found: 653.1207.

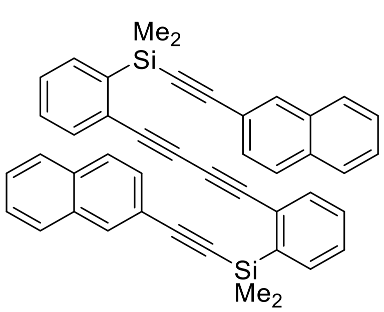

1,4-Bis(2-(dimethyl(naphthalen-2-ylethynyl)silyl)phenyl)buta-1,3-diyne (1i). Isolated by column chromatography on silica gel (hexane/toluene = 30/1 to 5/1). The title compound was obtained as a brown

solid (54%). Mp 135-136 °C; ^{1}H NMR δ 8.09 (s, 2H), 7.92-7.96 (m, 2H), 7.72-7.82 (m, 6H), 7.54-7.60 (m, 4H), 7.44-7.51 (m, 4H), 7.34-7.44 (m, 4H), 0.67 (s, 12H); ^{13}C NMR δ 140.4, 134.9, 133.1, 132.7, 132.5, 132.0, 129.1, 128.3, 128.2, 127.6, 127.5, 127.4, 126.5, 126.4, 126.2, 120.0, 107.4, 91.9, 83.1, 77.3, -1.0; HRMS (ESI, positive) calcd. for $C_{44}H_{34}NaSi_2$ $[M+Na]^+$: 641.2091; found: 641.2090.

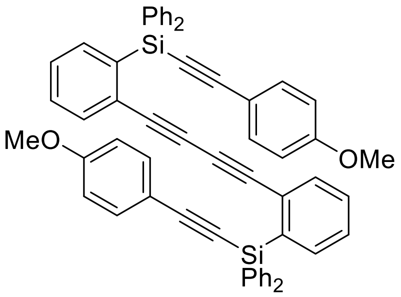

1,4-Bis(2-(((4-methoxyphenyl)ethynyl)diphenylsilyl)phenyl)buta-1,3-diyne (1j). Isolated by column chromatography on silica gel (hexane/toluene = 3/1 to 1/3). The title compound was obtained as a pale yellow solid (53%). Mp 159-160 °C; ^{1}H NMR δ 7.67-7.73 (m, 10H), 7.48-7.53 (m, 4H), 7.28-7.43 (m, 18H), 6.73-6.78 (m, 4H), 3.76 (s, 6H); ^{13}C NMR δ 160.2, 137.5, 137.0, 135.9, 134.1, 133.3, 129.9, 129.9, 128.4, 128.3, 128.0, 115.2, 113.9, 110.1, 87.6, 83.4, 79.2, 55.4; HRMS (ESI, positive) calcd. for $C_{58}H_{43}O_2Si_2$ $[M+H]^+$: 827.2796; found: 827.2789.

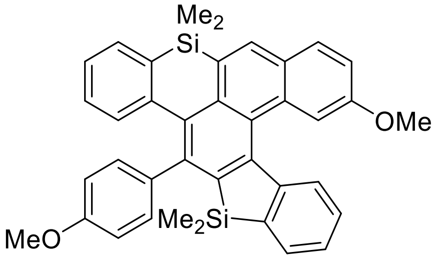

6-Methoxy-15-(4-methoxyphenyl)-10,10,16,16-tetramethyl-10,16-dihydrobenzo[*e*]benzo[4',5']silolo[2',3':3,4]phenanthro[10,1-*bc*]siline (2a). Isolated by preparative TLC (hexane/toluene = 1/2). The title compound was obtained as a yellow solid (81%). Mp 242-243 °C; ^{1}H NMR δ 8.16 (d, J = 2.5 Hz, 1H), 8.03-8.09 (m, 1H), 7.88 (s, 1H), 7.73-7.76 (m, 1H), 7.59-7.62 (m, 1H), 7.54-7.58 (m, 1H), 7.21-7.29 (m, 2H), 7.10-7.18 (m, 4H), 7.03-7.10 (m, 1H), 6.85-6.94 (m, 3H), 3.85 (s, 3H), 3.71 (s, 3H), 0.72 (s, 3H), 0.68 (s, 3H), 0.31 (s, 3H), -0.27 (s, 3H); ^{13}C NMR δ 158.4, 156.0, 149.4, 144.2, 144.1, 143.1, 142.6, 141.2, 138.3, 137.0, 135.5, 133.6, 132.7, 132.4, 132.1, 132.1, 131.9, 131.6, 129.7, 128.3, 128.2, 127.9, 126.8, 126.6, 126.0, 125.1, 117.6, 113.5, 111.3, 55.3, 55.2, 0.3, -0.2, -4.2, -4.6 (a pair of aromatic peaks is overlapped); HRMS (ESI, positive) calcd. for $C_{38}H_{34}NaO_2Si_2$ $[M+Na]^+$: 601.1990; found: 601.1989.

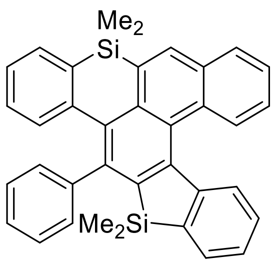

15-Phenyl-10,10,16,16-tetramethyl-10,16-dihydrobenzo[*e*]benzo[4',5']silolo[2',3':3,4]phenanthro[10,1-*bc*]siline (2b). Isolated by preparative TLC (hexane/toluene = 3/1). The title compound was obtained as a yellow solid (55%). Mp 195-196 °C; ^{1}H NMR δ 8.77-8.81 (m, 1H), 8.00-8.04 (m, 1H), 7.93 (s, 1H), 7.81-7.85 (m, 1H), 7.60-7.63 (m, 1H), 7.55-7.58 (m, 1H), 7.50-7.55 (m, 1H), 7.28-7.39 (m, 6H), 7.05-7.19 (m, 4H), 6.85-6.91 (m, 1H), 0.76 (s, 3H), 0.70 (s, 3H), 0.32 (s, 3H), -0.37 (s, 3H) ; ^{13}C NMR δ 149.7, 146.0, 144.7, 144.0, 142.9, 141.2, 136.4, 135.5, 133.8, 132.8, 132.7, 132.5, 132.4, 132.3, 131.1, 131.0, 130.5, 129.8, 128.7, 128.6, 128.4, 128.3, 128.1, 127.1, 126.9, 126.9, 126.7, 126.2, 125.4, 124.4, 0.7, 0.2, -0.6, -4.7; HRMS (ESI, positive) calcd. for $C_{36}H_{30}NaSi_2$ [M+Na]$^+$: 541.1778; found: 541.1780.

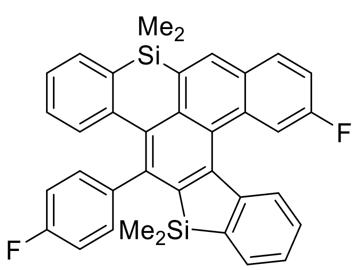

6-Fluoro-15-(4-fluorophenyl)-10,10,16,16-tetramethyl-10,16-dihydrobenzo[*e*]benzo[4',5']silolo[2',3':3,4]phenanthro[10,1-*bc*]siline (2c). Isolated by preparative TLC (hexane/toluene = 5/1). The title compound was obtained as a yellow solid (51%). Mp 256-257 °C; ^{1}H NMR δ 8.37-8.45 (m, 1H), 7.95-8.01 (m, 1H), 7.90 (s, 1H), 7.77-7.84 (m, 1H), 7.55-7.64 (m, 2H), 7.23-7.34 (m, 3H), 7.14-7.22 (m, 2H), 7.00-7.12 (m, 4H), 6.88-6.95 (m, 1H), 0.73 (s, 3H), 0.67 (s, 3H), 0.30 (s, 3H), -0.29 (s, 3H); ^{13}C NMR δ 163.0, 160.5, 158.1, 148.8, 144.2, 143.8, 142.8, 142.4, 141.7, 141.7, 140.9, 136.7, 135.4, 133.6, 132.7, 132.4, 132.3, 132.2, 132.0, 131.9, 131.9, 131.8, 131.7, 128.9, 128.8, 128.8, 128.1, 127.9, 127.9, 127.8, 127.8, 127.1, 126.0, 125.4, 116.0, 115.8, 115.2, 115.0, 114.8, 114.5, 0.3, -0.1, -4.4, -4.8; HRMS (ESI, positive) calcd. for $C_{36}H_{28}F_2NaSi_2$ [M+Na]$^+$: 577.1590; found: 577.1590.

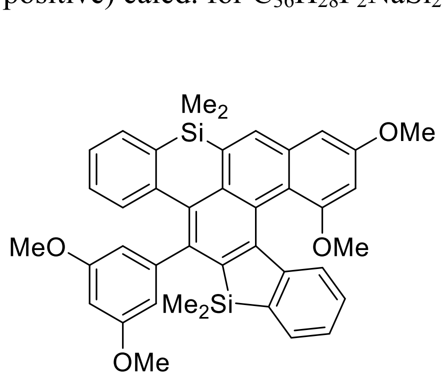

15-(3,5-Dimethoxyphenyl)-5,7-dimethoxy-10,10,16,16-tetramethyl-10,16-dihydrobenzo[*e*]benzo[4',5']silolo[2',3':3,4]phenanthro[10,1-*bc*]siline (2d). Isolated by preparative TLC

(hexane/toluene = 1/2). The title compound was obtained as a yellow solid (56%). Mp 239-240 °C; ^{1}H NMR δ 7.80 (s, 1H), 7.55-7.60 (m, 2H), 7.49-7.53 (m, 1H), 7.17-7.21 (m, 1H), 7.04-7.13 (m, 3H), 6.91-6.96 (m, 1H), 6.87-6.90 (m, 1H), 6.34-6.64 (m, 3H), 3.99 (s, 3H), 3.73 (brs, 6H), 3.44 (s, 3H), 0.75 (s, 3H), 0.72 (s, 3H), 0.24 (s, 3H), -0.30 (s, 3H) (a proton peak at the aromatic region is missing probably because it is broad); ^{13}C NMR δ 160.4, 159.2, 158.4, 151.4, 147.8, 145.7, 143.2, 143.0, 139.2, 138.6, 135.4, 134.3, 134.3, 133.5, 133.0, 132.1, 131.8, 131.3, 131.3, 128.5, 128.2, 126.1, 125.0, 124.1, 122.5, 116.4, 109.6, 98.9, 98.4, 98.3, 55.4, 55.3, 54.4, 0.7, 0.3, -4.8, -5.0; HRMS (ESI, positive) calcd. for $C_{40}H_{38}NaO_4Si_2$ $[M+Na]^+$: 661.2201; found: 661.2198.

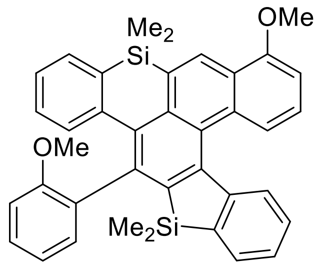

8-Methoxy-15-(2-methoxyphenyl)-10,10,16,16-tetramethyl-10,16-dihydrobenzo[*e*]benzo[4',5']silolo[2',3':3,4]phenanthro[10,1-*bc*]siline (2e). Isolated by preparative TLC (hexane/toluene = 1/1). The title compound was obtained as a yellow solid (52%). Mp 257-258 °C; ^{1}H NMR showed the presence of two conformers in a ratio of 1/2, δ 8.31-8.39 (m, 2H (both)), 8.00-8.05 (m, 1H (both)), 7.57-7.63 (m, 2H (major) + 1H (minor)), 7.52-7.55 (m, 1H (both)), 7.29-7.35 (m, 1H (both)), 6.79-7.26 (m, 8H (major) + 10H (minor)), 6.68-6.72 (m, 1H (major)), 4.06 (s, 3H (both)), 3.88 (s, 3H (minor)), 3.22 (s, 3H (major)), 0.78 (s, 3H (major)), 0.77 (s, 3H (minor)), 0.68 (s, 3H (major)), 0.43 (s, 3H (minor)), 0.26 (s, 3H (both)), -0.36 (s, 3H (minor)), -0.38 (s, 3H (major)); ^{13}C NMR both conformers shown δ 157.1, 156.4, 155.0, 149.8, 144.5, 143.5, 143.0, 142.9, 142.4, 141.0, 139.4, 135.7, 135.2, 134.6, 134.3, 134.0, 132.8, 131.9, 131.9, 131.7, 131.6, 131.4, 131.0, 128.7, 128.4, 128.2, 127.4, 126.5, 126.4, 126.1, 125.7, 125.5, 125.4, 125.2, 124.1, 124.0, 122.7, 122.5, 122.1, 122.0, 120.4, 120.2, 116.2, 111.5, 109.7, 106.1, 106.0, 106.0, 55.6, 55.1, 54.7, 0.6, 0.2, -2.1, -3.8, -4.7, -5.1, -5.4; HRMS (ESI, positive) calcd. for $C_{38}H_{34}NaO_2Si_2$ $[M+Na]^+$: 601.1990; found: 601.1978.

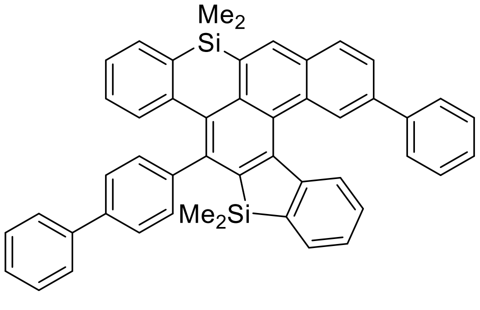

15-([1,1'-Biphenyl]-4-yl)-10,10,16,16-tetramethyl-6-phenyl-10,16-dihydrobenzo[*e*]benzo[4',5']silolo[2',3':3,4]phenanthro[10,1-*bc*]siline (2f). Isolated by preparative TLC (hexane/toluene = 2/1). The title compound was obtained as a yellow solid (43%). Mp 259-260 °C; ^{1}H NMR δ 9.00 (s, 1H), 8.15-8.21 (m, 1H), 7.96 (s, 1H), 7.88-7.92 (m, 1H), 7.75-7.80 (m, 1H), 7.67-7.72 (m, 2H), 7.54-7.66 (m, 6H), 7.27-7.49 (m, 8H), 7.15-7.22 (m, 3H), 7.05-7.10 (m, 1H), 6.86-6.92 (m, 1H), 0.76 (s, 3H), 0.71

(s, 3H), 0.35 (s, 3H), -0.30 (s, 3H); ^{13}C NMR δ 149.6, 145.0, 144.3, 144.2, 142.8, 142.8, 141.2, 141.1, 140.6, 139.1, 136.7, 136.6, 135.3, 133.8, 132.9, 132.6, 132.3, 132.3, 132.3, 131.3, 131.3, 130.6, 130.2, 128.8, 128.7, 128.6, 128.6, 128.1, 128.1, 127.4, 127.3, 127.1, 127.0, 126.9, 126.6, 126.2, 125.9, 125.3, 0.6, 0.0, -4.4, -4.7; HRMS (ESI, positive) calcd. for $C_{48}H_{38}NaSi_2$ [M+Na]$^+$: 693.2404; found: 693.2399.

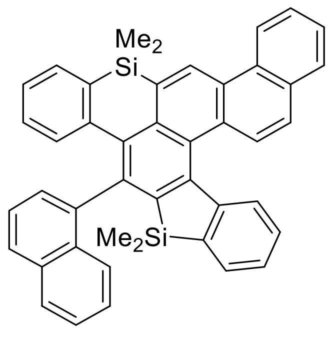

11-(Naphthalen-1-yl)-6,6,12,12-tetramethyl-6,12-dihydrobenzo[*e*]benzo[4',5']silolo[2',3':3,4]chryseno[12,1-*bc*]siline (2g). Isolated by preparative TLC (hexane/ethylacetate = 5/1). The title compound was obtained as a yellow solid. Mp 200-201 °C ; ^{1}H NMR showed the presence of two conformers in a ratio of 1/2, δ 8.80-8.91 (m, 2H (major) + 3H (minor)), 8.70-8.76 (m, 1H (both)), 7.26-8.00 (m, 13H (both)), 6.98-7.18 (m, 3H (both)), 6.87-6.93 (m, 1H (major)), 6.60-6.68 (m, 2H (both)), 0.88 (s, 3H (major)), 0.83 (s, 3H (minor)), 0.67 (s, 3H (major)), 0.42 (s, 3H (major)), 0.40 (s, 3H (minor)), -0.33 (s, 3H (minor)), -0.42 (s, 3H (minor)), -0.92 (s, 3H (major)) ; ^{13}C NMR both conformers shown δ 150.0, 149.8, 145.3, 144.4, 143.5, 143.3, 141.5, 136.5, 135.1, 134.0, 133.9, 133.8, 133.2, 133.1, 132.9, 132.6, 132.2, 132.2, 131.7, 130.1, 129.9, 129.6, 129.4, 129.2, 129.1, 129.1, 129.0, 129.0, 128.7, 128.5, 128.5, 128.4, 128.3, 128.3, 127.8, 127.3, 127.3, 127.1, 127.0, 127.0, 126.7, 126.5, 126.2, 126.2, 126.1, 125.2, 124.8, 123.8, 1.4, 0.9, 0.7, -1.0, -2.9, -3.3, -4.2, -5.0; HRMS (ESI, positive) calcd. for $C_{44}H_{34}Si_2$ (M): 618.2194 ; found: 618.2182.

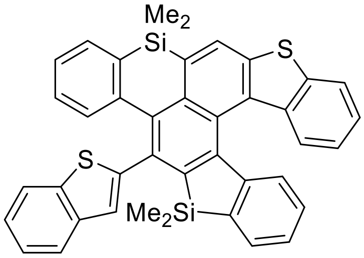

6-(Benzo[*b*]thiophen-2-yl)-5,5,11,11-tetramethyl-5,11-dihydrobenzo[*b*]benzo[4',5']silolo[2',3':7,8]benzo[5',6']silino[2',3',4':4,5]naphtho[1,2-*d*]thiophene (2h). Isolated by preparative TLC (hexane/toluene = 3/1). The title compound was obtained as a yellow solid (51%). Mp 206-207 °C; ^{1}H NMR δ 8.14 (s, 1H), 7.90-7.94 (m, 1H), 7.82-7.86 (m, 1H), 7.70-7.77 (m, 2H), 7.65-7.69 (m, 1H), 7.55-7.61 (m, 2H), 7.45 (s, 1H), 7.37-7.42 (m, 1H), 7.29-7.36 (m, 2H), 7.06-7.20 (m, 4H), 6.91-6.96 (m, 1H), 6.84-6.89 (m, 1H), 0.87 (s, 3H), 0.79 (s, 3H), 0.34 (s, 3H), -0.21 (s, 3H); ^{13}C NMR δ 148.9, 148.4, 143.7, 143.5, 142.8, 140.9, 139.9, 139.7, 138.5, 137.8, 136.7, 136.0, 134.6, 134.6, 134.4, 134.3, 132.5, 132.3, 131.8, 130.5, 128.8, 128.5, 127.2, 126.6, 126.4, 125.8, 125.2, 124.3, 124.0, 123.5, 122.5, 122.3, 0.5, 0.3, -4.7,

-5.0 (four pairs of aromatic peaks are overlapped); HRMS (ESI, positive) calcd. for $C_{40}H_{30}S_2Si_2$ $[M]^+$: 630.1322; found: 630.1307.

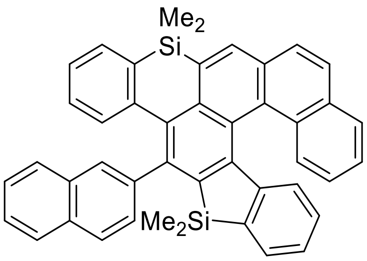

17-(Naphthalen-2-yl)-12,12,18,18-tetramethyl-12,18-dihydrobenzo[*e*]benzo[5,6]benzo[4',5']silolo[2',3':3,4]phenanthro[10,1-*bc*]siline (2i). Isolated by preparative TLC (hexane/toluene = 3/1). The title compound was obtained as a yellow solid (42%). Mp 286-287 °C; ^{1}H NMR δ 8.56 (d, J = 8.4 Hz, 1H), 8.09-8.12 (m, 1H), 8.07 (s, 1H), 7.99 (d, J = 8.4 Hz, 1H), 7.91-7.96 (m, 3H), 7.89 (d, J = 8.1 Hz, 1H), 7.78 (d, J = 8.1 Hz, 1H), 7.64-7.67 (m, 1H), 7.51-7.58 (m, 2H), 7.35-7.46 (m, 3H), 7.24-7.28 (m, 1H), 7.20-7.22 (m, 1H), 7.03-7.06 (m, 1H), 6.90-6.93 (m, 1H), 6.77-6.78 (m, 1H), 6.63 (d, J = 8.0 Hz, 1H), 6.55-6.59 (m, 1H), 0.84 (s, 3H), 0.83 (s, 3H), 0.40 (s, 3H), -0.53 (s, 3H); ^{13}C NMR δ 148.5, 146.1, 143.8, 142.8, 141.1, 139.9, 137.6, 134.9, 133.8, 133.4, 133.2, 132.4, 132.3, 132.1, 132.0, 131.8, 131.5, 130.6, 130.2, 129.9, 129.4, 129.0, 128.3, 128.1, 128.0, 127.9, 127.9, 127.8, 127.7, 127.0, 126.3, 126.0, 125.9, 125.8, 125.8, 125.7, 125.5, 125.4, 125.2, 0.7, 0.6, -4.6, -5.0 (a pair of aromatic peaks is overlapped); HRMS (ESI, positive) calcd. for $C_{44}H_{34}NaSi_2$ $[M+Na]^+$: 641.2091; found: 641.2080.

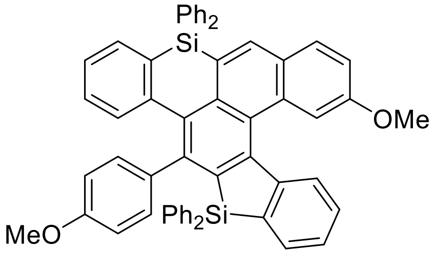

6-Methoxy-15-(4-methoxyphenyl)-10,10,16,16-tetraphenyl-10,16-dihydrobenzo[*e*]benzo[4',5']silolo[2',3':3,4]phenanthro[10,1-*bc*]saline (2j). Isolated by pipet column (hexane/toluene = 1/1). The title compound was obtained as a yellow solid (47%). Mp 176-177 °C; ^{1}H NMR δ 8.15 (d, J = 2.4 Hz, 1H), 7.99-8.02 (m, 1H), 7.97 (s, 1H), 7.69-7.72 (m, 1H), 7.07-7.65 (m, 26H), 6.96-7.01 (m, 1H), 6.85-6.90 (m, 1H), 6.76-6.81 (m, 2H), 6.45-6.49 (m, 2H), 3.72 (s, 3H), 3.65 (s, 3H); ^{13}C NMR δ 158.5, 156.5, 151.2, 147.0, 145.0, 144.9, 140.7, 138.6, 138.4, 137.6, 136.9, 136.4, 136.0, 135.9, 135.9, 134.7, 134.5, 134.4, 134.1, 133.9, 133.5, 133.0, 132.9, 132.7, 132.1, 129.9, 129.9, 129.8, 129.2, 128.8, 128.5, 128.1, 128.0, 128.0, 127.7, 127.4, 126.9, 126.6, 125.4, 125.2, 117.7, 113.5, 111.8, 55.4, 55.3; HRMS (ESI, positive) calcd. for $C_{58}H_{42}O_2NaSi_2$ $[M+Na]^+$: 849.2616; found: 849.2620.

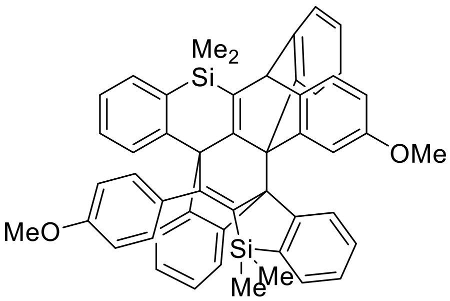

6-Methoxy-15-(4-methoxyphenyl)-10,10,16,16-tetramethyl-9*H*,10*H*,16*H*-4b,14b:4c,9-bis([1,2]benzeno)benzo[*e*]benzo[4',5']silolo[2',3':3,4]phenanthro[10,1-*bc*]siline (4). Isolated by preparative TLC (hexane/toluene = 1/1). The title compound was obtained as a yellow solid (58%). Mp 148-149 °C; ^{1}H NMR δ 8.51 (d, *J* = 7.4 Hz, 1H), 7.83-7.87 (m, 1H), 7.45-7.54 (m, 3H), 7.34-7.42 (m, 2H), 7.04-7.20 (m, 5H), 6.88-7.02 (m, 3H), 6.71-6.77 (m, 1H), 6.62-6.70 (m, 2H), 6.34-6.51 (m, 5H), 5.15 (s, 1H), 3.61 (s, 3H), 3.24 (s, 3H), 0.35 (s, 3H), 0.28 (s, 3H), 0.21 (s, 3H), -0.08 (s, 3H); ^{13}C NMR δ 163.5, 159.2, 157.6, 155.8, 150.9, 150.3, 148.5, 147.2, 146.0, 145.9, 144.4, 143.7, 143.3, 138.9, 134.9, 134.7, 133.5, 132.8, 130.7, 130.2, 129.5, 129.0, 128.2, 127.3, 127.1, 125.8, 125.3, 125.2, 124.9, 124.7, 123.3, 122.8, 122.2, 121.6, 121.4, 112.4, 112.2, 109.5, 68.0, 65.0, 61.5, 55.3, 54.9, 53.3, 2.4, -1.5, -2.3, -3.2; HRMS (ESI, positive) calcd. for $C_{50}H_{42}O_2Si_2$ $[M]^+$: 730.2723; found: 730.3035.

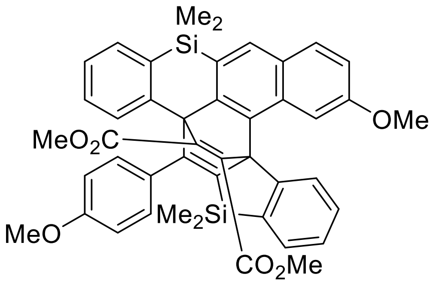

6-Methoxy-15-(4-methoxyphenyl)-10,10,16,16-tetramethyl-17,18-bis(methoxycarbonyl)-10*H*,16*H*-4b,14b-ethenobenzo[*e*]benzo[4',5']silolo[2',3':3,4]phenanthro[10,1-*bc*]siline (5). Isolated by preparative TLC (hexane/ethylacetate = 5/1). The title compound was obtained as a yellow solid (38%). Mp 82-83 °C ;^{1}H NMR δ 8.05-8.09 (m, 1H), 8.00 (s, 1H), 7.68-7.78 (m, 2H), 7.36-7.54 (m, 7H), 7.04-7.11 (m, 3H), 6.65-6.70 (m, 2H), 3.76 (s, 3H), 3.61 (s, 3H), 3.47 (s, 3H), 3.24 (s, 3H), 0.61 (s, 3H), 0.47 (s, 3H), 0.01 (s, 3H), -0.43 (s, 3H); ^{13}C NMR δ 171.0, 169.5, 160.1, 158.5, 146.9, 144.4, 139.0, 138.0, 136.9, 136.2, 135.6, 134.0, 133.8, 133.8, 133.3, 133.1, 131.3, 130.7, 130.3, 130.1, 129.9, 129.8, 129.7, 128.3, 128.0, 127.9, 127.7, 119.1, 115.5, 114.1, 114.1, 110.0, 106.7, 92.1, 55.7, 55.3, 53.1, 52.5, 0.1, -1.1, -1.9, -2.8 ; HRMS (ESI, positive) calcd. for $C_{44}H_{40}NaO_6Si_2$ $[M+Na]^+$: 743.2256; found: 743.2250.

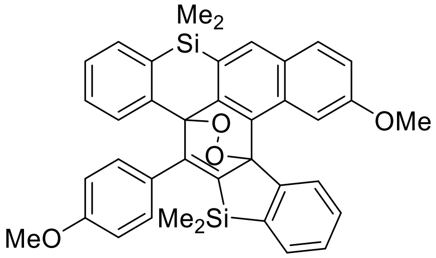

6-Methoxy-15-(4-methoxyphenyl)-10,10,16,16-tetramethyl-10*H*,16*H*-4b,14b-epidioxybenzo[*e*]benzo[4',5']silolo[2',3':3,4]phenanthro[10,1-*bc*]siline (6). Isolated by preparative TLC (toluene). The title compound was obtained as a yellow solid (48%). Mp 222-223 °C ;^{1}H NMR δ 8.02 (s, 1H), 7.85 (d, *J* = 7.7 Hz, 2H), 7.77-7.81 (m, 1H), 7.73 (d, *J* = 8.9 Hz, 1H), 7.59-7.63 (m, 2H), 7.53-7.57 (m, 1H), 7.37 (d, *J* = 7.8 Hz, 1H), 7.24-7.28 (m, 1H), 7.10-7.14 (m, 1H), 6.97 (dd, *J* =9.0 Hz, 2.4 Hz, 1H), 6.79 (d, *J* = 8.9, 2H), 6.58 (d, *J* = 8.9, 2H), 3.70 (s, 3H), 3.20 (s, 3H), 0.66 (s, 3H), 0.62 (s, 3H), 0.56 (s, 3H), -0.11 (s, 3H) ; ^{13}C NMR δ 162.9, 158.5, 157.4, 147.5, 145.9, 143.9, 142.3, 139.4, 138.7, 135.2, 133.3, 132.9, 132.9, 132.8, 132.1, 130.6, 130.2, 130.2, 129.7, 129.6, 129.0, 128.9, 128.1, 127.9, 124.8, 118.9, 112.9, 103.1, 91.4, 84.8, 55.1, 54.4, 1.5, 0.5, -0.7, -1.9; HRMS (ESI, positive) calcd. for $C_{38}H_{34}NaO_4Si_2$ (M+Na): 633.1888 ; found: 633.1884.

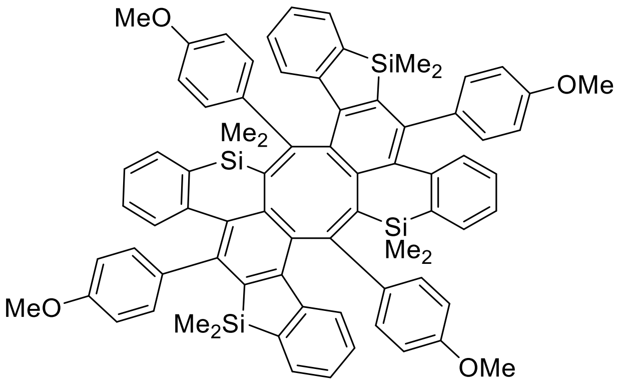

(8a*E*, 17a*Z*)-Bis((1, 1-dimethyl-1*H*-benzo[*b*]siloleno)[*2,3-h:2',3'-h'*])-3,9,12,18-tetrakis(4-methoxyphenyl)-8,8,17,17-tetramethyl-8,17-dihydro-8,17-disilacycloocta[1,2,3-*jk*:5,6,7-*j'k'*]diphenanthrene (3a). Isolated by preparative TLC (hexane/toluene = 1/2). The title compound was obtained as an orange solid (32%). Mp > 280 °C; ^{1}H NMR δ 7.49-7.56 (m, 4H), 7.45 (d, *J* = 7.3 Hz, 2H), 7.35 (d, *J* = 7.3 Hz, 2H), 7.11 (d, *J* = 8.7 Hz, 4H), 7.03-7.08 (m, 2H), 6.92 (dd, *J* = 2.6, 8.4 Hz, 2H), 6.79-6.89 (m, 4H), 6.73-6.78 (m, 2H), 6.70 (d, *J* = 8.7 Hz, 4H), 6.54 (dd, *J* = 2.6, 8.4 Hz, 2H), 6.43 (dd, *J* = 2.0, 8.6 Hz, 2H), 6.06-6.12 (m, 2H), 3.77 (s, 6H), 3.76 (s, 6H), 0.38 (s, 6H), 0.10 (s, 6H), -0.33 (s, 6H), -0.43 (s, 6H); ^{13}C NMR δ 159.1, 158.0, 152.7, 149.1, 145.2, 144.6, 144.1, 143.9, 141.6, 141.1, 140.1, 138.5, 137.1, 135.4, 134.4, 132.4, 132.2, 132.0, 131.8, 131.6, 131.6, 129.4, 128.4, 126.1, 126.0, 125.4, 113.1, 112.6, 55.2, 55.1, 0.6, -1.9, -3.8, -4.5(two pair of aromatic peaks are overlapped); HRMS (ESI, positive) calcd. for $C_{76}H_{68}NaO_4Si_4$ $[M+Na]^+$: 1179.4087; found: 1179.4088.

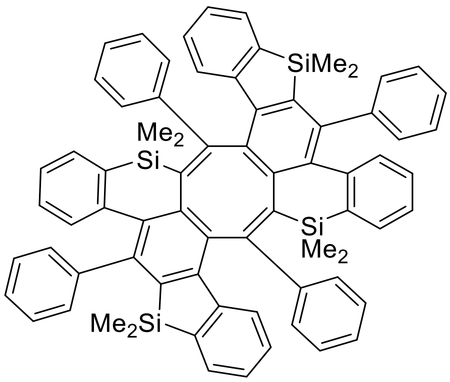

(8a*E*, 17a*Z*)-Bis((1, 1-dimethyl-1*H*-benzo[*b*]siloleno)[*2,3-h:2',3'-h'*])-3,9,12,18-

tetraphenyl-8,8,17,17-tetramethyl-8,17-dihydro-8,17-disilacycloocta[1,2,3-*jk*:5,6,7-*j'k'*]diphenanthrene (3b). Isolated by preparative TLC (hexane/toluene = 2/1). The title compound was obtained as an orange solid (49%). Mp > 280 °C; ^{1}H NMR δ 7.60-7.64 (m, 2H), 7.53-7.57 (m, 2H), 7.42-7.46 (m, 2H), 7.34-7.41 (m, 4H), 7.14-7.26 (m, 12H), 7.03-7.08 (m, 2H), 6.95-7.01 (m, 2H), 6.84-6.89 (m, 2H), 6.75-6.83 (m, 4H), 6.50-6.54 (m, 2H), 6.05-6.11 (m, 2H), 0.38 (s, 6H), 0.07 (s, 6H), -0.38 (s, 6H), -0.45 (s, 6H); ^{13}C NMR δ 153.1, 149.0, 145.8, 144.5, 144.5, 144.0, 144.0, 142.9, 141.6, 140.1, 139.6, 138.3, 135.1, 134.3, 132.3, 132.1, 131.7, 130.9, 130.6, 130.4, 129.4, 128.4, 127.6, 127.5, 127.4, 127.3, 126.3, 126.2, 126.0, 125.7, 0.7, -2.0, -4.0, -4.7; HRMS (ESI, positive) calcd. for $C_{72}H_{60}NaSi_4$ $[M+Na]^+$: 1059.3664; found: 1059.3661.

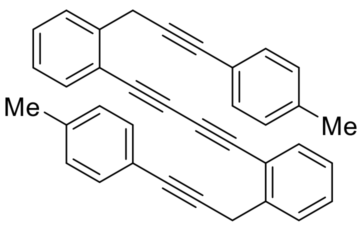

1,4-Bis(2-(3-(*p*-tolyl)prop-2-yn-1-yl)phenyl)buta-1,3-diyne (S1). Isolated by column chromatography on silica gel (hexane/toluene = 5/1). The title compound was obtained as a pale yellow solid (47%). Mp 140-141 °C; ^{1}H NMR δ 7.69 (d, *J* = 7.7 Hz, 2H), 7.55 (dd, *J* = 1.2, 7.7 Hz, 2H), 7.35-7.42 (m, 6H), 7.23-7.27 (m, 2H), 7.10-7.13 (m, 4H), 4.04 (s, 4H), 2.34 (s, 6H); ^{13}C NMR δ 140.5, 138.1, 133.2, 131.7, 129.8, 129.2, 128.4, 126.9, 121.0, 120.7, 85.9, 83.6, 80.7, 78.7, 24.9, 21.6; HRMS (NSI, positive) calcd. for $C_{36}H_{30}N$ $[M+NH_4]^+$: 476.2373; found: 476.2369.

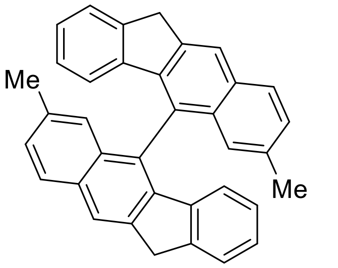

7,7'-Dimethyl-11*H*,11'*H*-5,5'-bibenzo[*b*]fluorene (S2). Isolated by preparative TLC (hexane/toluene = 3/1). The title compound was obtained as a yellow solid (57%). Mp 268-269 °C; ^{1}H NMR δ 8.15 (s, 2H), 7.88-7.92 (m, 2H), 7.40-7.44 (m, 2H), 7.26-7.30 (m, 2H), 7.08 (s, 2H), 7.01-7.06 (m, 2H), 6.66-6.71 (m, 2H), 5.96-6.00 (m, 2H), 4.20 (s, 4H), 2.17 (s, 6H); ^{13}C NMR δ 144.3, 141.4, 140.9, 138.9, 135.4, 132.9, 131.6, 129.2, 128.2, 127.7, 126.9, 126.8, 125.1, 124.7, 123.4, 123.3, 36.7, 22.0; HRMS (NSI, positive) calcd. for $C_{36}H_{30}N$ $[M+NH_4]^+$: 476.2373; found: 476.2374.

vii. DFT calculation

1) Theoretical calculation of NICS(0) values for 2a and 6

NICS(0) values were calculated at the GIAO B3LYP/6-311+G(2d,p)//B3LYP/6-31G(d) level of theory using Gaussian 09 program. [3] The results of X-ray crystallographic analysis for **2a** and **6** were used for calculations.

2) Theoretical calculation of transition states of the modeled reactions

For DFT calculations for energy diagrams were performed at the UB3LYP-D3BJ level of theory, [4] using Gaussian 16 program. [5] Geometry optimizations was carried out with 6-31+G(d,p). Vibration frequency was computed at the same level of theory to confirm whether the structures are minima (no imaginary frequencies) or transition states (only one imaginary frequency). Energies of optimized structures were calculated by single point calculation at the same level of theory with 6-311++G(2d,2p) and solvation effects (toluene, ε = 2.3741) using SMD model.[6] All values in the energy diagrams are relative free energies (kcal/mol) at 423.15 K. The molecular geometries of the transition states were first estimated by the Reaction plus pro software package (Software to optimize reaction paths along the user's expected ones, HPC Systems Inc., http://www.hpc.co.jp/chem/react1.html (written in Japanese)), based on the nudged elastic band (NEB) method[7], and subsequently re-optimized by the Synchronous Transit-guided Quasi-Newton method with the keyword QST2 or QST3.[8] Transition-state structures were confirmed to connect corresponding reactants and products through the use of intrinsic reaction coordination (IRC) calculations.

3) Structures optimized at UB3LYP/6-31G+(d,p)

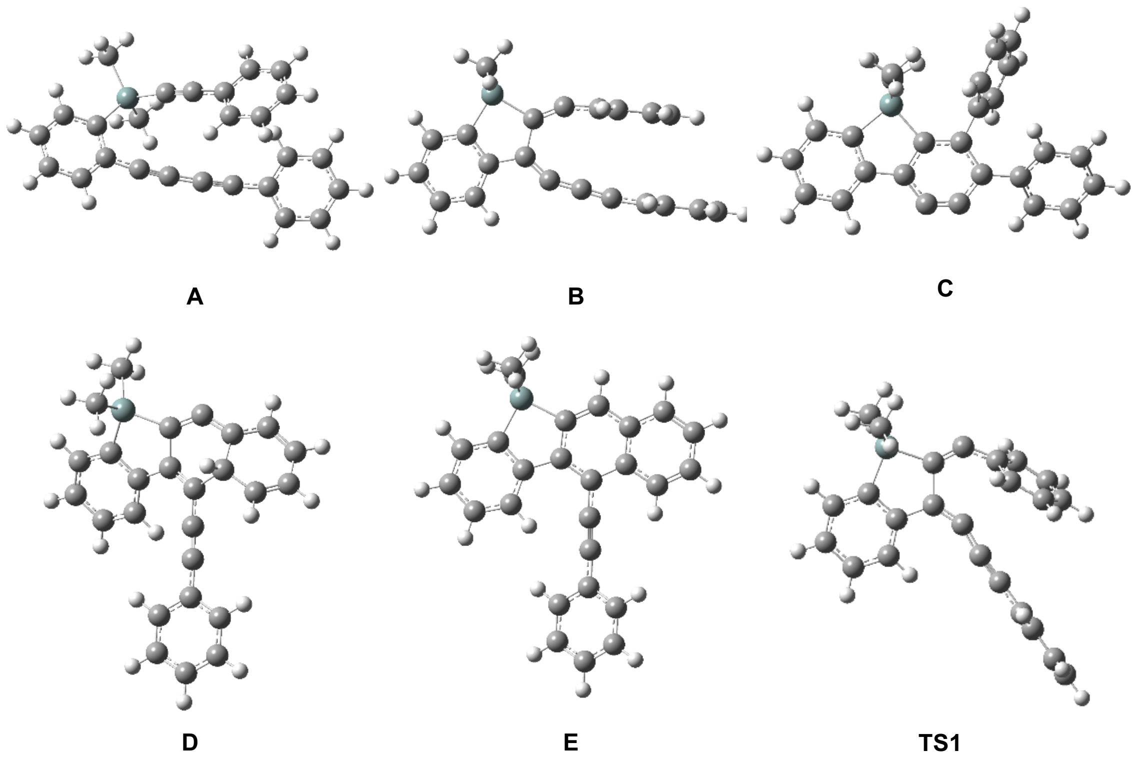

Figure S1. Structure optimized at UB3LYP/6-31G+(d,p). The hydrogen atom colored in white, the carbon atoms in gray, the silicon atom in skyblue.

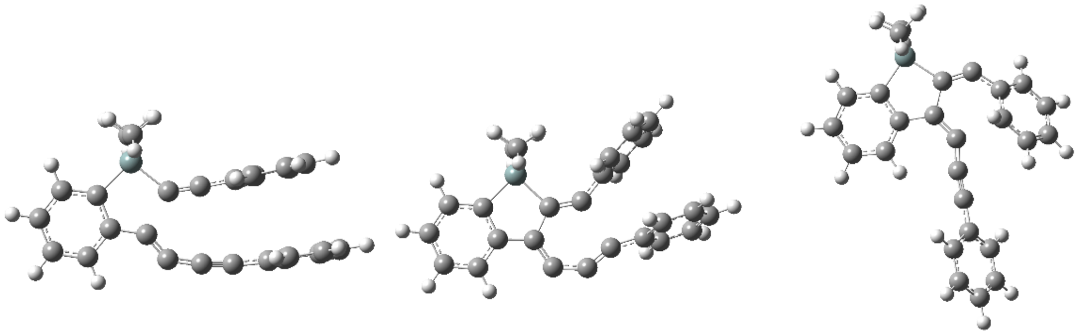

TS2 TS3 TS4

Figure S1 (Cont.) Structure optimized at UB3LYP/6-31G+(d,p). The hydrogen atom colored in white, the carbon atoms in gray, the silicon atom in skyblue.

4) Total electronic energy and free energy with solvation effects (toluene, ε = 2.3741) using SMD model

	E (Hartree)	S (cal/mol)	G (Hartree)
**A**	-1292.649794	178.486	-1292.375207
**B**	-1292.605218	179.47	-1292.334094
**C**	-1292.696129	163.375	-1292.410412
**D**	-1292.618574	169.342	-1292.338451
**E**	-1292.767548	167.467	-1292.483636
**TS1**	-1292.593001	172.772	-1292.317582
**TS2**	-1292.606593	174.357	-1292.332288
**TS3**	-1292.563804	172.629	-1292.28872
**TS4**	-1292.71303	173.313	-1292.297075

5) Cartesian coordinates of optimized structures

A (NIMG = 0)

Center Number	Atomic Number	Atomic Type	Coordinates (Angstroms) X	Y	Z
1	6	0	5.942659	-0.830489	-1.039916
2	6	0	5.353314	-1.980507	-1.568991
3	6	0	4.009657	-2.247035	-1.320554
4	6	0	3.241319	-1.365127	-0.535941
5	6	0	3.828721	-0.192987	0.017908
6	6	0	5.182424	0.044574	-0.258282
7	6	0	1.303001	1.495023	0.363874

8	6	0	0.201169	1.743551	-0.099146
9	6	0	-1.094615	1.973258	-0.649283
10	6	0	-1.936747	2.972264	-0.123433
11	6	0	-3.211695	3.166611	-0.653393
12	6	0	-3.663918	2.371426	-1.710144
13	6	0	-2.833087	1.378684	-2.237806
14	6	0	-1.557878	1.177801	-1.715962
15	6	0	1.863125	-1.636898	-0.318824
16	6	0	0.663108	-1.752892	-0.122755
17	6	0	-0.678450	-1.786964	0.084265
18	6	0	-1.887122	-1.747245	0.257233
19	6	0	-3.288837	-1.615283	0.444715
20	6	0	-3.841761	-0.343485	0.702385
21	6	0	-5.215779	-0.202199	0.874757
22	6	0	-6.056728	-1.316643	0.795913
23	6	0	-5.515495	-2.580969	0.541533
24	6	0	-4.142255	-2.734629	0.366311
25	1	0	6.989064	-0.614443	-1.233543
26	1	0	5.936756	-2.666179	-2.175790
27	1	0	3.537652	-3.133512	-1.731405
28	1	0	5.661608	0.932574	0.142763
29	1	0	-1.581497	3.584492	0.698785
30	1	0	-3.852998	3.939168	-0.239875
31	1	0	-4.659864	2.519402	-2.115822
32	1	0	-3.184554	0.750904	-3.050562
33	1	0	-0.914820	0.399313	-2.109972
34	1	0	-3.187401	0.518621	0.752327
35	1	0	-5.630897	0.782312	1.066979
36	1	0	-7.127802	-1.201268	0.931078
37	1	0	-6.165300	-3.448547	0.479585
38	1	0	-3.716469	-3.712481	0.167715
39	14	0	2.870711	0.975985	1.158470
40	6	0	3.914642	2.505098	1.502398
41	1	0	4.836269	2.251023	2.037454
42	1	0	4.181783	3.027759	0.578361
43	1	0	3.346176	3.201403	2.127952
44	6	0	2.458061	0.086496	2.765332
45	1	0	3.375219	-0.227361	3.276181
46	1	0	1.896201	0.745552	3.435781
47	1	0	1.849923	-0.801142	2.570268

B (NIMG = 0)

Center Number	Atomic Number	Atomic Type	Coordinates (Angstroms) X	Y	Z
1	6	0	0.862716	1.464312	-0.000207
2	6	0	2.108464	1.539122	-0.000262
3	6	0	-0.450504	1.398108	-0.000144
4	6	0	-1.598513	0.720399	-0.000130
5	6	0	-1.610305	-0.793076	-0.000401
6	6	0	-0.524940	-1.529674	-0.000597
7	6	0	-4.036320	0.399072	0.000155
8	6	0	-2.952534	1.311408	0.000124
9	6	0	-5.343926	0.899431	0.000395
10	1	0	-6.189036	0.215090	0.000418
11	6	0	-3.191739	2.694218	0.000335
12	1	0	-2.352212	3.383382	0.000307
13	6	0	-5.580140	2.275847	0.000609
14	1	0	-6.597866	2.654897	0.000792
15	6	0	-4.500241	3.169529	0.000576
16	1	0	-4.684474	4.239894	0.000737
17	6	0	0.844915	-1.765835	-0.000014
18	6	0	1.569004	-1.885763	1.223855
19	6	0	1.569988	-1.886705	-1.223199
20	6	0	2.943402	-2.070173	1.212266
21	1	0	1.029059	-1.798466	2.160666
22	6	0	2.944380	-2.071082	-1.210370
23	1	0	1.030800	-1.800148	-2.160515
24	6	0	3.644237	-2.159923	0.001263
25	1	0	3.482384	-2.130620	2.153055
26	1	0	3.484123	-2.132231	-2.150677
27	1	0	4.722158	-2.281754	0.001744
28	6	0	3.507678	1.432353	-0.000305
29	6	0	4.226399	1.354422	1.218355
30	6	0	4.226352	1.354528	-1.218999
31	6	0	5.607063	1.194779	1.210786
32	1	0	3.679659	1.407826	2.153418
33	6	0	5.607016	1.194879	-1.211495

34	1	0	3.679577	1.408016	-2.154038
35	6	0	6.304873	1.111798	-0.000371
36	1	0	6.144787	1.131965	2.152115
37	1	0	6.144704	1.132139	-2.152850
38	1	0	7.383106	0.986159	-0.000398
39	14	0	-3.414208	-1.372479	-0.000402
40	6	0	-3.870029	-2.339800	1.547751
41	1	0	-4.945138	-2.550425	1.580002
42	1	0	-3.337500	-3.297068	1.574374
43	1	0	-3.605100	-1.775918	2.447449
44	6	0	-3.870639	-2.338649	-1.549100
45	1	0	-3.338403	-3.296054	-1.576568
46	1	0	-4.945823	-2.548934	-1.581165
47	1	0	-3.605837	-1.774197	-2.448478

C (NIMG = 0)

Center Number	Atomic Number	Atomic Type	Coordinates (Angstroms) X	Y	Z
1	6	0	5.813150	-0.848858	0.126276
2	6	0	5.324928	-2.154744	0.011705
3	6	0	3.951374	-2.389488	-0.079163
4	6	0	3.068027	-1.305960	-0.054622
5	6	0	3.547880	0.022316	0.062198
6	6	0	4.924659	0.233862	0.149375
7	6	0	1.605705	-1.418172	-0.130172
8	6	0	0.850008	-0.199308	-0.123971
9	6	0	0.758166	-2.515955	-0.166734
10	6	0	-0.492480	-2.491726	-0.159430
11	6	0	-1.344141	-1.391940	-0.152145
12	6	0	-0.557972	-0.182880	-0.122272
13	6	0	-1.233650	1.144368	0.003083
14	6	0	-2.818835	-1.479836	-0.136075
15	6	0	-1.910337	1.482158	1.183342
16	6	0	-2.513544	2.731980	1.322310
17	6	0	-2.452685	3.661719	0.279964
18	6	0	-1.784853	3.331666	-0.901696
19	6	0	-1.179491	2.080440	-1.037854

20	6	0	-3.647174	-0.563713	-0.805829
21	6	0	-5.034234	-0.704859	-0.772889
22	6	0	-5.622474	-1.760798	-0.072627
23	6	0	-4.808529	-2.686125	0.586802
24	6	0	-3.422282	-2.548445	0.550402
25	1	0	6.882901	-0.675513	0.197261
26	1	0	6.017228	-2.991223	-0.006913
27	1	0	3.566004	-3.401416	-0.168589
28	1	0	5.319817	1.243168	0.237823
29	1	0	-1.960219	0.757821	1.990158
30	1	0	-3.031443	2.980399	2.243777
31	1	0	-2.923473	4.634066	0.387657
32	1	0	-1.737350	4.045210	-1.719069
33	1	0	-0.671427	1.817160	-1.959924
34	1	0	-3.208838	0.254620	-1.362587
35	1	0	-5.656121	0.011469	-1.301359
36	1	0	-6.702837	-1.865671	-0.046509
37	1	0	-5.253012	-3.515193	1.129281
38	1	0	-2.789206	-3.267627	1.061846
39	14	0	2.093534	1.223939	0.029397
40	6	0	2.222110	2.378853	-1.453869
41	1	0	3.180197	2.910712	-1.419613
42	1	0	2.184149	1.818293	-2.393258
43	1	0	1.420764	3.122707	-1.455966
44	6	0	1.860458	2.203618	1.617914
45	1	0	0.920656	2.764473	1.583596
46	1	0	1.829513	1.536313	2.484664
47	1	0	2.680498	2.916055	1.762014

D (NIMG = 0)

Center Number	Atomic Number	Atomic Type	Coordinates (Angstroms) X	Y	Z
1	6	0	1.281031	0.441819	0.137412
2	6	0	-0.044047	0.902370	0.106452
3	6	0	-1.130659	0.116704	-0.158351
4	6	0	-2.492827	0.680384	0.164833
5	6	0	-1.170823	-1.323437	-0.482923

6	6	0	2.449884	0.094151	0.190712
7	6	0	-2.551362	2.005910	0.086034
8	6	0	-2.398226	-1.971012	-0.168766
9	6	0	-0.289283	-3.417908	-1.322219
10	6	0	-0.124331	-2.056971	-1.065085
11	6	0	3.783735	-0.392569	0.231363
12	6	0	-1.530482	2.888799	-0.042345
13	6	0	-2.533558	-3.338414	-0.423904
14	1	0	0.802889	-1.564710	-1.330391
15	1	0	-0.419002	2.232662	1.715189
16	6	0	0.905466	3.303260	0.465762
17	1	0	-2.369736	4.225283	-1.524617
18	6	0	-0.303379	4.652722	-1.170760
19	1	0	4.693543	1.563403	0.250620
20	1	0	7.017405	0.695757	0.319448
21	6	0	0.881437	4.339258	-0.398977
22	6	0	6.181968	0.002576	0.297055
23	1	0	3.187821	-2.466034	0.224456
24	6	0	4.879576	0.494610	0.260998
25	6	0	5.336138	-2.264191	0.271758
26	6	0	-1.486142	-4.065170	-0.997452
27	1	0	-3.463659	-3.847567	-0.182208
28	6	0	-0.269679	2.389149	0.635505
29	1	0	-1.603226	-5.125926	-1.199076
30	6	0	6.416248	-1.376149	0.301641
31	6	0	-1.468536	3.970858	-0.975333
32	6	0	4.030148	-1.782260	0.238763
33	1	0	0.519176	-3.976277	-1.785439
34	1	0	5.512977	-3.335494	0.277680
35	1	0	-0.260177	5.460980	-1.894264
36	1	0	1.775805	3.121238	1.087872
37	1	0	7.432925	-1.755718	0.329145
38	1	0	1.752644	4.980010	-0.501988
39	14	0	-3.635679	-0.755455	0.565436
40	6	0	-3.899328	-1.013391	2.413140
41	1	0	-4.493632	-0.197545	2.839251
42	1	0	-4.427411	-1.954220	2.606255
43	1	0	-2.939565	-1.048523	2.938097
44	6	0	-5.279544	-0.685317	-0.344256
45	1	0	-5.850625	-1.609268	-0.197903

46	1	0	-5.887687	0.148685	0.023209
47	1	0	-5.121501	-0.545046	-1.417786

E (NIMG = 0)

Center Number	Atomic Number	Atomic Type	Coordinates (Angstroms) X	Y	Z
1	6	0	1.188761	-4.323430	-0.222580
2	6	0	-0.046265	-3.679951	-0.322510
3	6	0	-0.135843	-2.287379	-0.271064
4	6	0	1.020889	-1.506066	-0.115473
5	6	0	2.281728	-2.163119	-0.034259
6	6	0	2.349092	-3.556351	-0.084157
7	6	0	1.083582	-0.014733	-0.050898
8	6	0	2.427489	0.501117	0.012495
9	6	0	0.006552	0.883430	-0.041694
10	6	0	0.255525	2.309744	-0.016129
11	6	0	1.598862	2.793123	0.018846
12	6	0	2.663960	1.853068	0.044182
13	6	0	-0.799136	3.258694	-0.020596
14	6	0	-0.537569	4.613712	0.000140
15	6	0	0.793550	5.089529	0.030319
16	6	0	1.838912	4.191216	0.040771
17	6	0	-1.353270	0.469596	-0.031687
18	6	0	-2.538320	0.190855	-0.001223
19	6	0	-3.898917	-0.225490	0.041297
20	6	0	-4.238225	-1.479784	0.589430
21	6	0	-5.567488	-1.894195	0.626264
22	6	0	-6.578151	-1.069315	0.122936
23	6	0	-6.251127	0.177125	-0.419673
24	6	0	-4.924318	0.599316	-0.462647
25	1	0	1.247372	-5.407181	-0.261647
26	1	0	-0.953414	-4.264373	-0.447171
27	1	0	-1.104032	-1.819997	-0.363941
28	1	0	3.313322	-4.055458	-0.020607
29	1	0	3.681962	2.235190	0.091926
30	1	0	-1.822374	2.902761	-0.040418
31	1	0	-1.361810	5.320588	-0.005449

32	1	0	0.987813	6.157514	0.047048
33	1	0	2.867644	4.540490	0.067191
34	1	0	-3.451777	-2.112630	0.987397
35	1	0	-5.816528	-2.861626	1.051680
36	1	0	-7.613242	-1.394954	0.154512
37	1	0	-7.032284	0.821222	-0.811834
38	1	0	-4.666423	1.563443	-0.888128
39	14	0	3.664766	-0.907584	0.076989
40	6	0	4.839338	-0.951899	-1.395147
41	1	0	5.532805	-0.103544	-1.370704
42	1	0	5.436157	-1.871127	-1.392675
43	1	0	4.281477	-0.910056	-2.335682
44	6	0	4.631754	-0.963676	1.692888
45	1	0	5.232399	-1.877927	1.759870
46	1	0	5.313606	-0.109201	1.771358
47	1	0	3.952411	-0.937784	2.550454

TS1 (NIMG = 1, 104.50*i* cm^{-1})

Center Number	Atomic Number	Atomic Type	Coordinates (Angstroms) X	Y	Z
1	6	0	-3.281850	-3.807490	-0.563702
2	6	0	-1.885900	-3.777206	-0.674731
3	6	0	-1.188757	-2.585009	-0.494172
4	6	0	-1.891756	-1.403889	-0.209068
5	6	0	-3.301956	-1.422125	-0.093066
6	6	0	-3.980094	-2.634238	-0.270915
7	6	0	-2.281861	1.065337	0.116524
8	6	0	-1.849426	2.289612	-0.021803
9	6	0	-0.555179	2.783486	-0.088274
10	6	0	0.002852	3.426276	-1.232155
11	6	0	1.356215	3.675445	-1.291840
12	6	0	2.205662	3.337383	-0.205911
13	6	0	1.689313	2.729040	0.920962
14	6	0	0.323689	2.383264	0.969479
15	6	0	-1.213288	-0.099527	0.001985
16	6	0	0.074677	0.083980	0.142813
17	6	0	1.351843	-0.319463	0.131615

18	6	0	2.560361	-0.566730	0.121911
19	6	0	3.939897	-0.872416	0.117155
20	6	0	4.677455	-0.857811	-1.089203
21	6	0	6.037008	-1.154225	-1.086304
22	6	0	6.691635	-1.467685	0.109995
23	6	0	5.972338	-1.484755	1.309644
24	6	0	4.611461	-1.192857	1.319548
25	1	0	-3.817981	-4.741479	-0.704151
26	1	0	-1.340598	-4.688375	-0.902695
27	1	0	-0.106918	-2.563242	-0.576310
28	1	0	-5.063364	-2.668598	-0.181990
29	1	0	-0.654433	3.698473	-2.051155
30	1	0	1.780455	4.143389	-2.175065
31	1	0	3.263324	3.576133	-0.260654
32	1	0	2.335152	2.487650	1.758700
33	1	0	-0.101518	1.982732	1.881391
34	1	0	4.167144	-0.611352	-2.014208
35	1	0	6.590256	-1.140291	-2.020554
36	1	0	7.752486	-1.697596	0.106981
37	1	0	6.474654	-1.728963	2.240838
38	1	0	4.049849	-1.209570	2.247599
39	14	0	-3.975211	0.280557	0.313613
40	6	0	-4.644047	0.400963	2.069644
41	1	0	-5.560384	-0.189899	2.182088
42	1	0	-3.908391	0.028810	2.789531
43	1	0	-4.877255	1.440719	2.324205
44	6	0	-5.230638	0.929742	-0.925743
45	1	0	-6.168896	0.366113	-0.868273
46	1	0	-5.455862	1.983256	-0.727522
47	1	0	-4.842690	0.849685	-1.945663

TS2 (NIMG = 1, 369.24*i* cm^{-1})

Center Number	Atomic Number	Atomic Type	Coordinates (Angstroms) X	Y	Z
1	6	0	6.101236	-1.081379	-0.156838
2	6	0	5.440072	-2.308834	-0.295540
3	6	0	4.050512	-2.368930	-0.274544

4	6	0	3.297151	-1.189270	-0.114359
5	6	0	3.956006	0.051771	0.026826
6	6	0	5.356257	0.087370	0.003634
7	6	0	1.195592	0.524733	0.054726
8	6	0	-0.008657	0.869566	0.006676
9	6	0	-1.345316	1.298414	-0.073464
10	6	0	-2.163697	1.360381	1.080724
11	6	0	-3.465703	1.836785	0.993720
12	6	0	-3.986899	2.262212	-0.232625
13	6	0	-3.192736	2.196000	-1.382446
14	6	0	-1.890015	1.715681	-1.313293
15	6	0	1.844218	-1.255498	-0.087739
16	6	0	0.912930	-2.133615	-0.125717
17	6	0	-0.430752	-2.168926	-0.092831
18	6	0	-1.655323	-2.050850	-0.048925
19	6	0	-3.034760	-1.761659	0.007647
20	6	0	-3.732046	-1.802723	1.236475
21	6	0	-5.075116	-1.445768	1.294640
22	6	0	-5.746516	-1.034490	0.138396
23	6	0	-5.067576	-0.995952	-1.083488
24	6	0	-3.726673	-1.355884	-1.155195
25	1	0	7.186225	-1.041058	-0.174466
26	1	0	6.013581	-3.222549	-0.421070
27	1	0	3.530970	-3.315241	-0.380791
28	1	0	5.875411	1.036926	0.111475
29	1	0	-1.760865	1.021115	2.028744
30	1	0	-4.084927	1.864300	1.884580
31	1	0	-5.005888	2.630193	-0.293148
32	1	0	-3.595516	2.513447	-2.339614
33	1	0	-1.272609	1.659136	-2.203524
34	1	0	-3.201806	-2.111236	2.131140
35	1	0	-5.599541	-1.480088	2.244874
36	1	0	-6.793188	-0.750448	0.188678
37	1	0	-5.585053	-0.673302	-1.981718
38	1	0	-3.191982	-1.315301	-2.097368
39	14	0	2.785436	1.489883	0.236458
40	6	0	2.945024	2.778695	-1.122761
41	1	0	3.923232	3.271462	-1.077562
42	1	0	2.843180	2.314050	-2.108197
43	1	0	2.171978	3.547993	-1.022018

44	6	0	2.890827	2.281837	1.938488
45	1	0	3.867879	2.756810	2.084926
46	1	0	2.117534	3.047933	2.059802
47	1	0	2.757057	1.529493	2.721712

TS3 (NIMG = 1, 613.91*i* cm^{-1})

Center Number	Atomic Number	Atomic Type	Coordinates (Angstroms) X	Y	Z
1	6	0	-0.212415	-2.702063	-0.313922
2	6	0	-1.267281	-2.021012	-0.224593
3	6	0	1.174016	-2.489085	-0.313012
4	6	0	-2.684011	-1.796815	-0.133558
5	6	0	1.795494	-1.296602	-0.171692
6	6	0	-3.378407	-2.181104	1.028138
7	6	0	-3.383079	-1.153335	-1.169725
8	6	0	3.256799	-1.080005	-0.105245
9	6	0	-4.744359	-1.929835	1.144463
10	1	0	-2.834636	-2.670905	1.828928
11	6	0	-4.745424	-0.895894	-1.043076
12	1	0	-2.844694	-0.847411	-2.059194
13	6	0	3.677534	0.259661	0.092822
14	6	0	4.203560	-2.107542	-0.222366
15	6	0	-5.429751	-1.281353	0.113344
16	1	0	-5.272598	-2.233528	2.043173
17	1	0	-5.273248	-0.389655	-1.845186
18	6	0	5.046835	0.536544	0.172118
19	14	0	2.190421	1.403754	0.200611
20	1	0	3.871220	-3.129709	-0.378028
21	6	0	5.562057	-1.809087	-0.139817
22	1	0	-6.491525	-1.076498	0.210656
23	1	0	5.388935	1.557668	0.323427
24	6	0	5.988502	-0.489366	0.057688
25	6	0	1.003220	-0.062286	-0.036070
26	6	0	1.966574	2.243631	1.869034
27	6	0	2.099725	2.666664	-1.192463
28	1	0	6.294632	-2.605801	-0.230460
29	1	0	7.049384	-0.265502	0.119754

30	6	0	-0.328556	0.005958	-0.032997
31	1	0	2.729066	3.013651	2.032721
32	1	0	0.983284	2.724157	1.922322
33	1	0	2.035783	1.514155	2.681766
34	1	0	1.148964	3.209292	-1.158462
35	1	0	2.910862	3.399345	-1.109485
36	1	0	2.183196	2.176301	-2.167355
37	6	0	-1.337446	1.005961	-0.013221
38	6	0	-2.161662	1.192053	1.124190
39	6	0	-1.604857	1.778201	-1.172048
40	6	0	-3.194862	2.119964	1.103912
41	1	0	-1.979387	0.587568	2.006046
42	6	0	-2.639252	2.707438	-1.180170
43	1	0	-0.989199	1.627559	-2.052894
44	6	0	-3.442295	2.881260	-0.046256
45	1	0	-3.818269	2.248585	1.983510
46	1	0	-2.825762	3.296742	-2.073227
47	1	0	-4.255851	3.599736	-0.059422

TS4 (NIMG = 1, 473.58*i* cm^{-1})

Center Number	Atomic Number	Atomic Type	Coordinates (Angstroms) X	Y	Z
1	6	0	1.398993	-0.093130	0.141269
2	6	0	0.131854	0.374985	0.207883
3	6	0	-1.140160	-0.095114	0.147931
4	6	0	-2.327166	0.770253	0.353250
5	6	0	-1.501534	-1.495716	-0.210615
6	6	0	2.608301	-0.345864	0.138855
7	6	0	-2.224619	2.102565	0.319559
8	6	0	-2.887185	-1.800359	-0.178208
9	6	0	-1.020751	-3.779463	-0.855043
10	6	0	-0.575340	-2.493810	-0.550326
11	6	0	3.979763	-0.686150	0.122964
12	6	0	-1.077123	2.934804	0.189018
13	6	0	-3.310385	-3.097928	-0.482349
14	1	0	0.483876	-2.266692	-0.581075
15	1	0	0.032512	1.930240	1.751736

16	6	0	1.362712	3.072685	0.483480
17	1	0	-1.945185	4.444167	-1.070226
18	6	0	0.195450	4.684077	-0.909839
19	1	0	4.510511	0.824756	-1.325554
20	1	0	7.767254	-1.616942	0.076728
21	1	0	2.281323	2.688601	0.916021
22	1	0	0.229684	5.552001	-1.560381
23	1	0	6.191936	-2.872962	1.535031
24	1	0	-0.298744	-4.546118	-1.120764
25	6	0	4.474039	-1.736522	0.931124
26	6	0	-1.025587	4.072526	-0.629004
27	6	0	6.713233	-1.357742	0.089654
28	1	0	-2.721959	-5.092999	-1.059805
29	6	0	0.132003	2.417384	0.784856
30	1	0	-4.369451	-3.344079	-0.459291
31	6	0	-2.385139	-4.088344	-0.821689
32	6	0	5.826318	-2.064054	0.909723
33	6	0	4.885956	0.020314	-0.701986
34	1	0	6.920184	0.233671	-1.353121
35	6	0	1.389072	4.163722	-0.366805
36	6	0	6.236046	-0.316443	-0.714092
37	1	0	3.783309	-2.280520	1.566650
38	1	0	2.336048	4.641676	-0.600086
39	14	0	-3.888055	-0.285050	0.294029
40	6	0	-4.733128	-0.438121	1.969898
41	1	0	-5.168298	0.520282	2.274170
42	1	0	-5.539290	-1.179983	1.935397
43	1	0	-4.017252	-0.748054	2.737465
44	6	0	-5.115095	0.267515	-1.020494
45	1	0	-5.946078	-0.441674	-1.107726
46	1	0	-5.534435	1.248106	-0.769216
47	1	0	-4.626357	0.344817	-1.996450

----

viii. References

[1] N. Sugimura, A. Furuya, T. Yatsu, T. Shibue, *Eur. J. Mass Spectrom.,* **2015**, *21*, 91-96.

[2] T. Shibata, T. Uchiyama, Y. Yoshinami, S. Takayasu, K. Tsuchikamaa and K. Endo, *Chem. Commun.*, **2012**, 48, 1311–1313.

[3] Gaussian 09, revision B.01; M. J. Frisch, G. W. Trucks, H. B. Schlegel, G. E. Scuseria, M. A. Robb, J. R. Cheeseman, G. Scalmani, V. Barone, B. Mennucci, G. A. Petersson, H. Nakatsuji, M. Caricato, X. Li, H.

P. Hratchian, A. F. Izmaylov, J. Bloino, G. Zheng, J. L. Sonnenberg, M. Hada, M. Ehara, K. Toyota, R. Fukuda, J. Hasegawa, M. Ishida, T. Nakajima, Y. Honda, O. Kitao, H. Nakai, T. Vreven, J. A. Montgomery, Jr., J. E. Peralta, F. Ogliaro, M. Bearpark, J. J. Heyd, E. Brothers, K. N. Kudin, V. N. Staroverov, T. Keith, R. Kobayashi, J. Normand, K. Raghavachari, A. Rendell, J. C. Burant, S. S. Iyengar, J. Tomasi, M. Cossi, N. Rega, J. M. Millam, M. Klene, J. E. Knox, J. B. Cross, V. Bakken, C. Adamo, J. Jaramillo, R. Gomperts, R. E. Stratmann, O. Yazyev, A. J. Austin, R. Cammi, C. Pomelli, J. W. Ochterski, R. L. Martin, K. Morokuma, V. G. Zakrzewski, G. A. Voth, P. Salvador, J. J. Dannenberg, S. Dapprich, A. D. Daniels, O. Farkas, J. B. Foresman, J. V. Ortiz, J. Cioslowski, and D. J. Fox, Gaussian, Inc., Wallingford CT, 2010.

[4] a) S. Grimme, J. Antony, S. Ehrlich, H. Krieg, *J. Chem. Phys.* **2010**, *132*, 154104 b) E. R. Johnson, A. D. Becke, *J. Chem. Phys.* **2006**, *124*, 174104

[5] Gaussian 16, Revision A.03, M. J. Frisch, G. W. Trucks, H. B. Schlegel, G. E. Scuseria, M. A. Robb, J. R. Cheeseman, G. Scalmani, V. Barone, G. A. Petersson, H. Nakatsuji, X. Li, M. Caricato, A. V. Marenich, J. Bloino, B. G. Janesko, R. Gomperts, B. Mennucci, H. P. Hratchian, J. V. Ortiz, A. F. Izmaylov, J. L. Sonnenberg, D. Williams-Young, F. Ding, F. Lipparini, F. Egidi, J. Goings, B. Peng, A. Petrone, T. Henderson, D. Ranasinghe, V. G. Zakrzewski, J. Gao, N. Rega, G. Zheng, W. Liang, M. Hada, M. Ehara, K. Toyota, R. Fukuda, J. Hasegawa, M. Ishida, T. Nakajima, Y. Honda, O. Kitao, H. Nakai, T. Vreven, K. Throssell, J. A. Montgomery, Jr., J. E. Peralta, F. Ogliaro, M. J. Bearpark, J. J. Heyd, E. N. Brothers, K. N. Kudin, V. N. Staroverov, T. A. Keith, R. Kobayashi, J. Normand, K. Raghavachari, A. P. Rendell, J. C. Burant, S. S. Iyengar, J. Tomasi, M. Cossi, J. M. Millam, M. Klene, C. Adamo, R. Cammi, J. W. Ochterski, R. L. Martin, K. Morokuma, O. Farkas, J. B. Foresman, and D. J. Fox, Gaussian, Inc., Wallingford CT, 2016.

[6] A. V. Marenich,; C. J. Cramer, D. G. Truhlar, *J. Phys. Chem. B* **2009**, *113*, 6378−6396.

[7] H. Jonsson, G. Mills, K. W. Jacobsen, Nudged Elastic Band´ Method for Finding Minimum Energy Paths of Transitions, in Classical and Quantum Dynamics in Condensed Phase Simulations, Ed. B. J. Berne, G. Ciccotti, D. F. Coker, 385 (World Scientific, 1998); G. Henkelman, H. Jonsson, Improved tangent estimate in the´ nudged elastic band method for finding minimum energy paths and saddle points. *J. Chem. Phys.* **2000**, *113*, 9978−9985.

[8] (a) C. Peng, H. B. Schlegel, *Isr. J. Chem*. **1993**, *33*, 449−454. (b) C. Peng, P. Y. Ayala, H. B. Schlegel, M. J. Frisch, *J. Comput. Chem.* **1996**, *17*, 49−56.

viii. Copies of ^{1}H and ^{13}C NMR spectra for new compounds

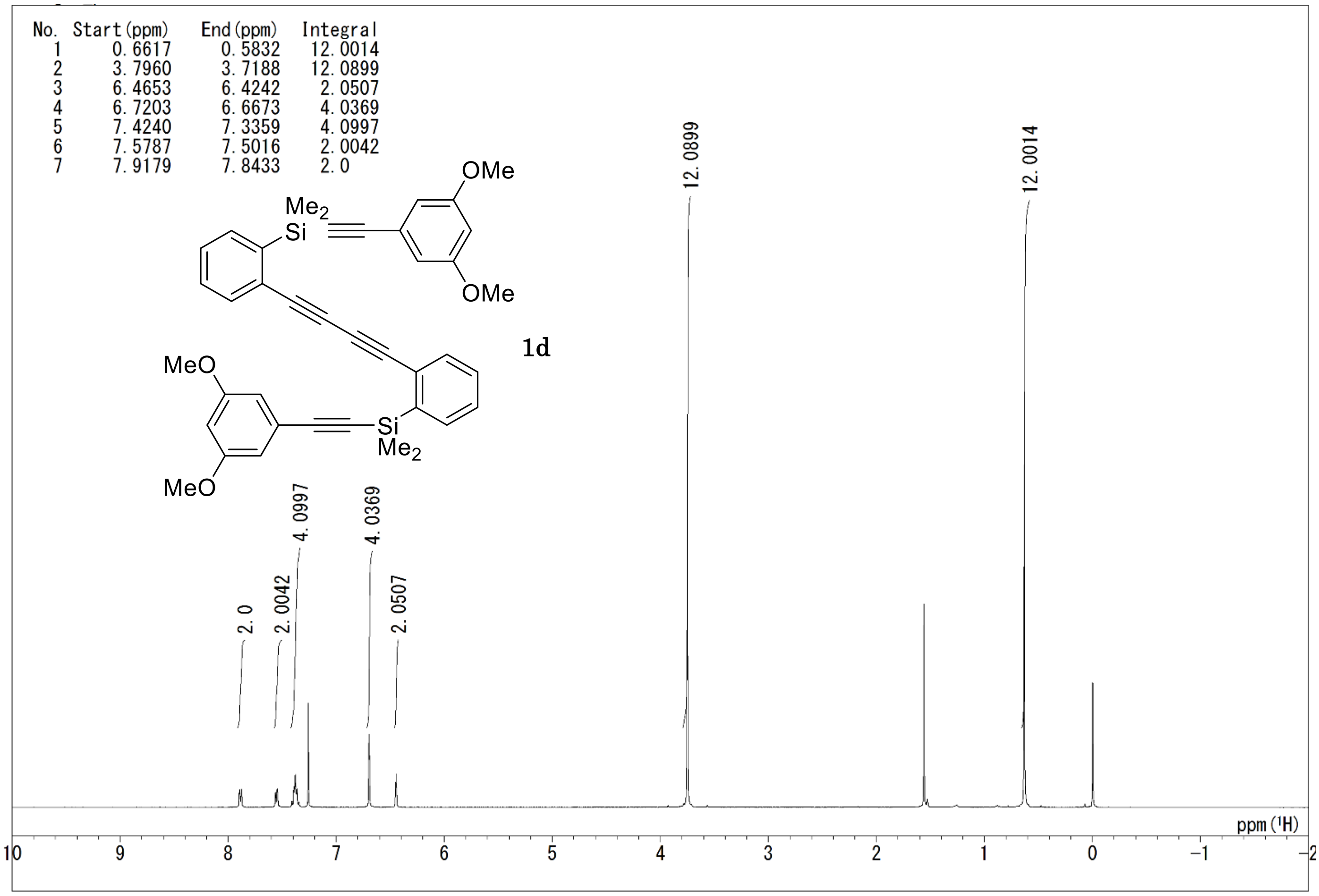

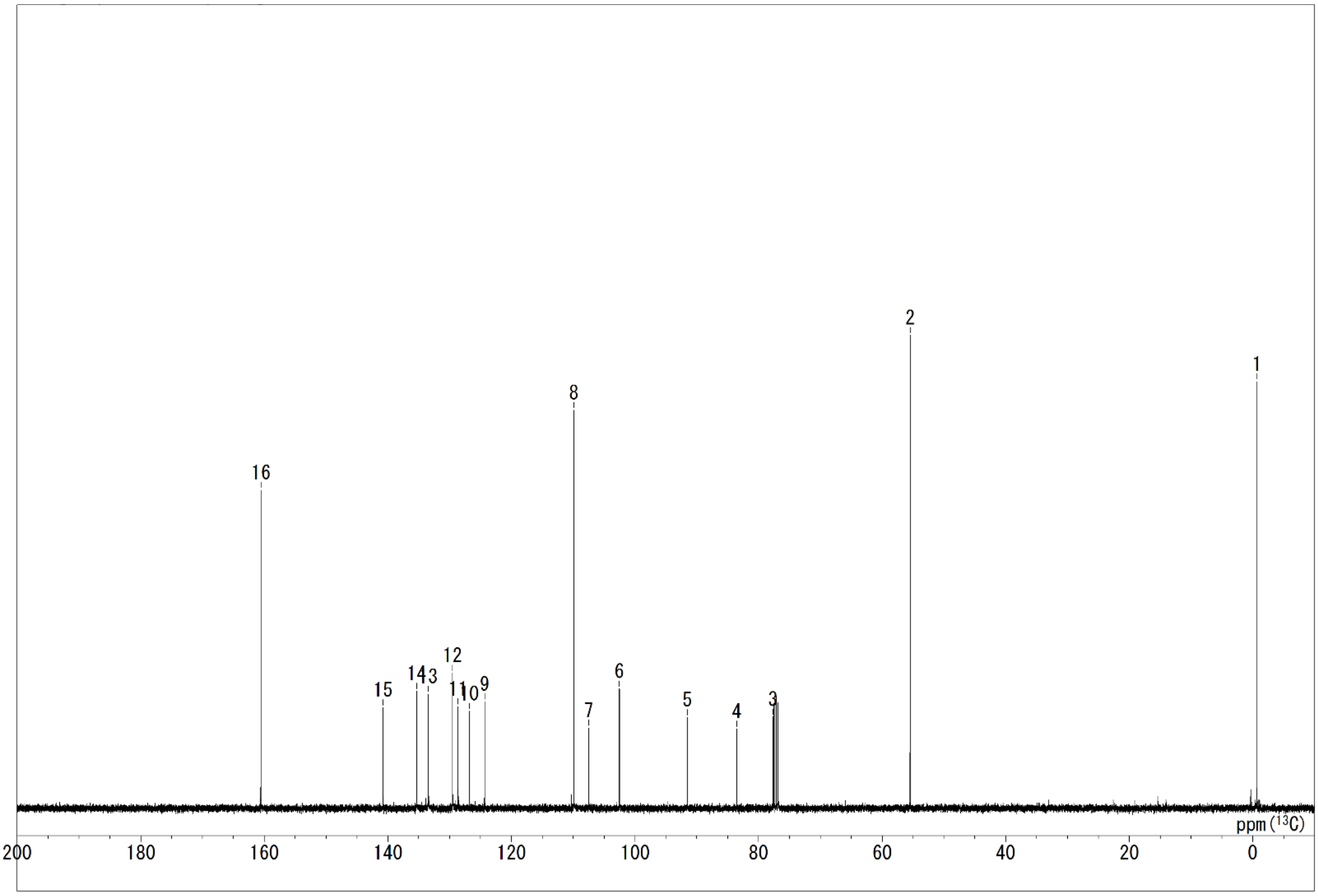

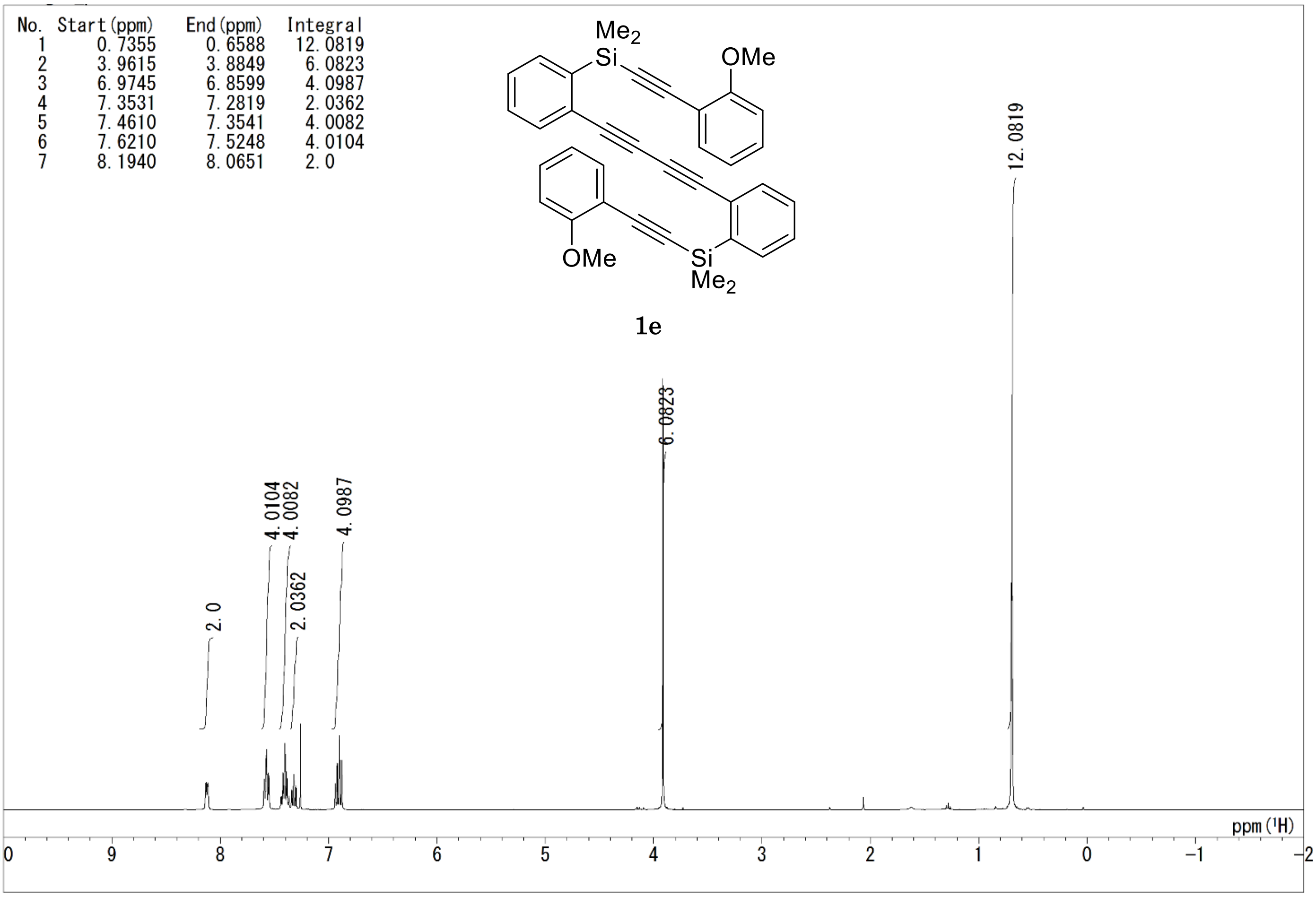

No.	Start (ppm)	End (ppm)	Integral
1	0.7355	0.6588	12.0819
2	3.9615	3.8849	6.0823
3	6.9745	6.8599	4.0987
4	7.3531	7.2819	2.0362
5	7.4610	7.3541	4.0082
6	7.6210	7.5248	4.0104
7	8.1940	8.0651	2.0

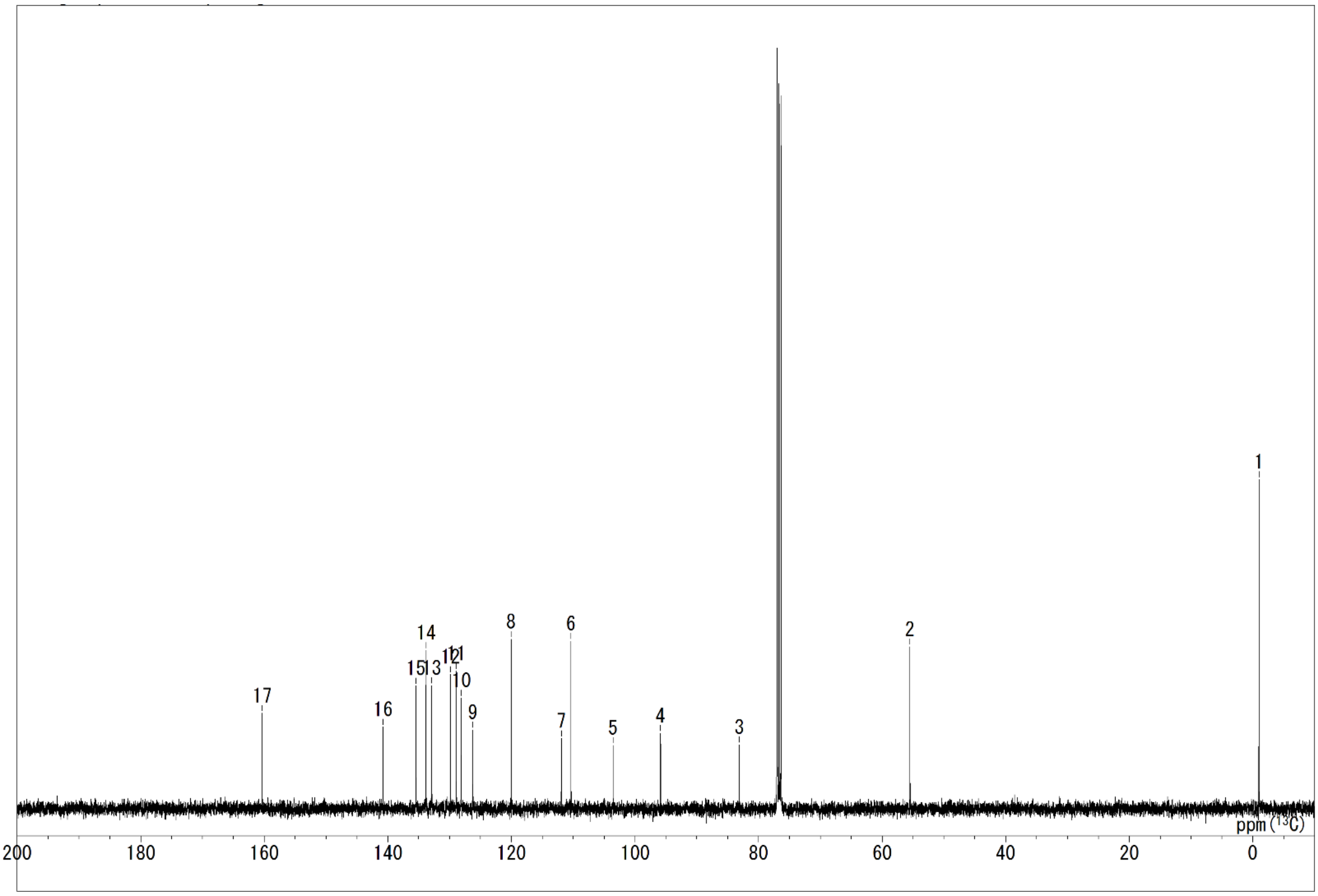

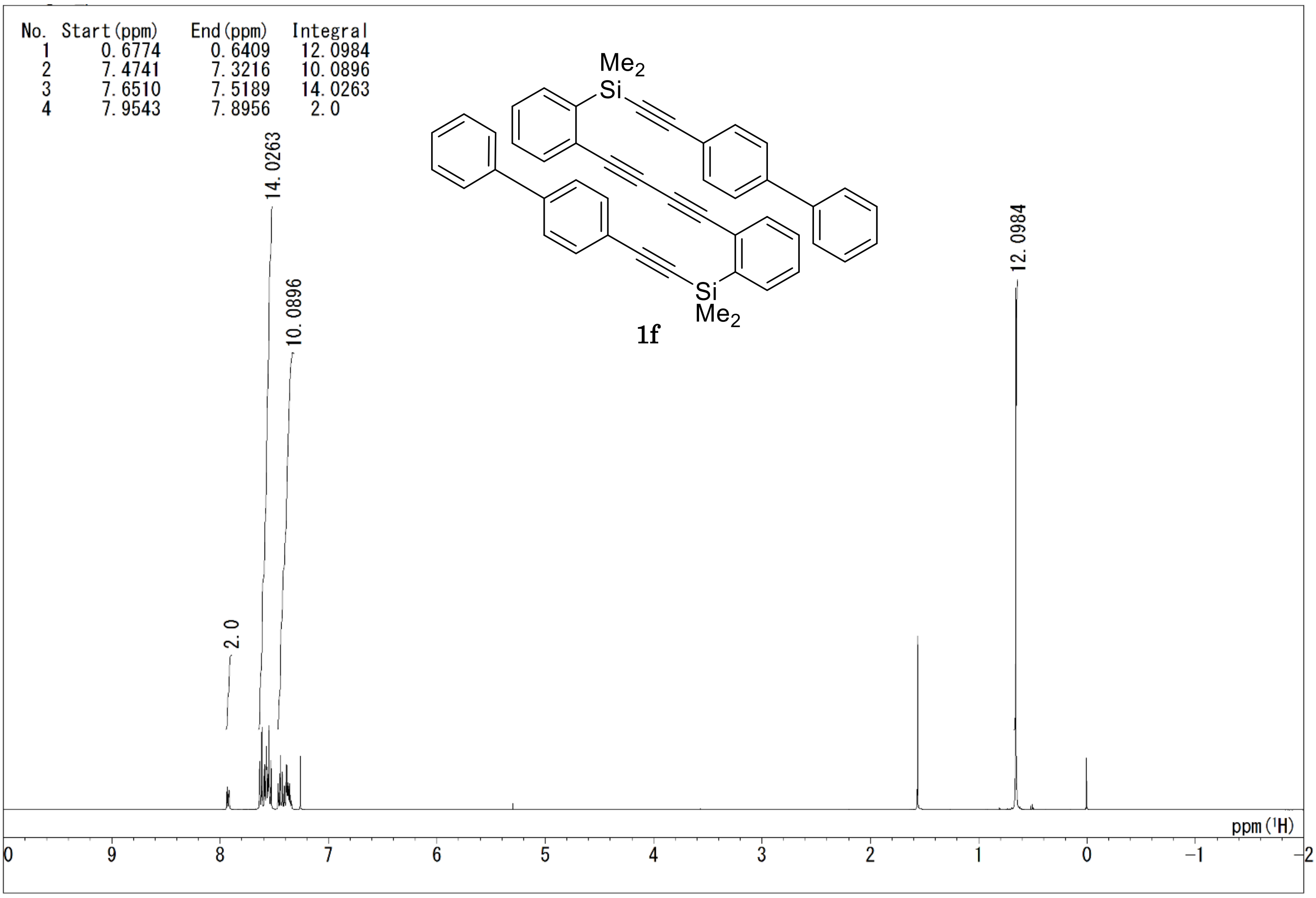

No. Start(ppm) End(ppm) Integral
1 0.6774 0.6409 12.0984
2 7.4741 7.3216 10.0896
3 7.6510 7.5189 14.0263
4 7.9543 7.8956 2.0
Me2
Si
Si
Me2
1f
14.0263
10.0896
2.0
12.0984
ppm (1H)

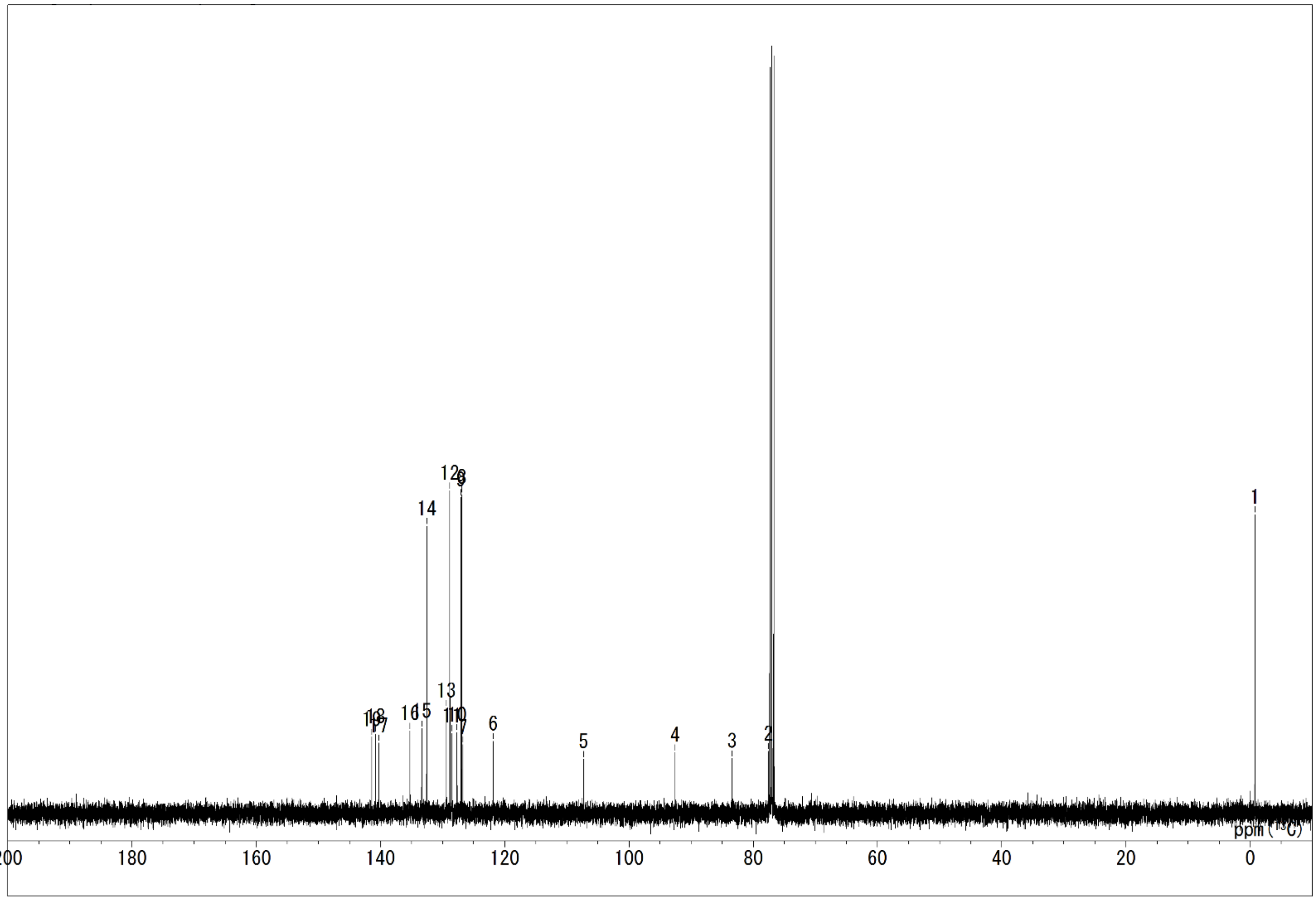

ppm (13C)

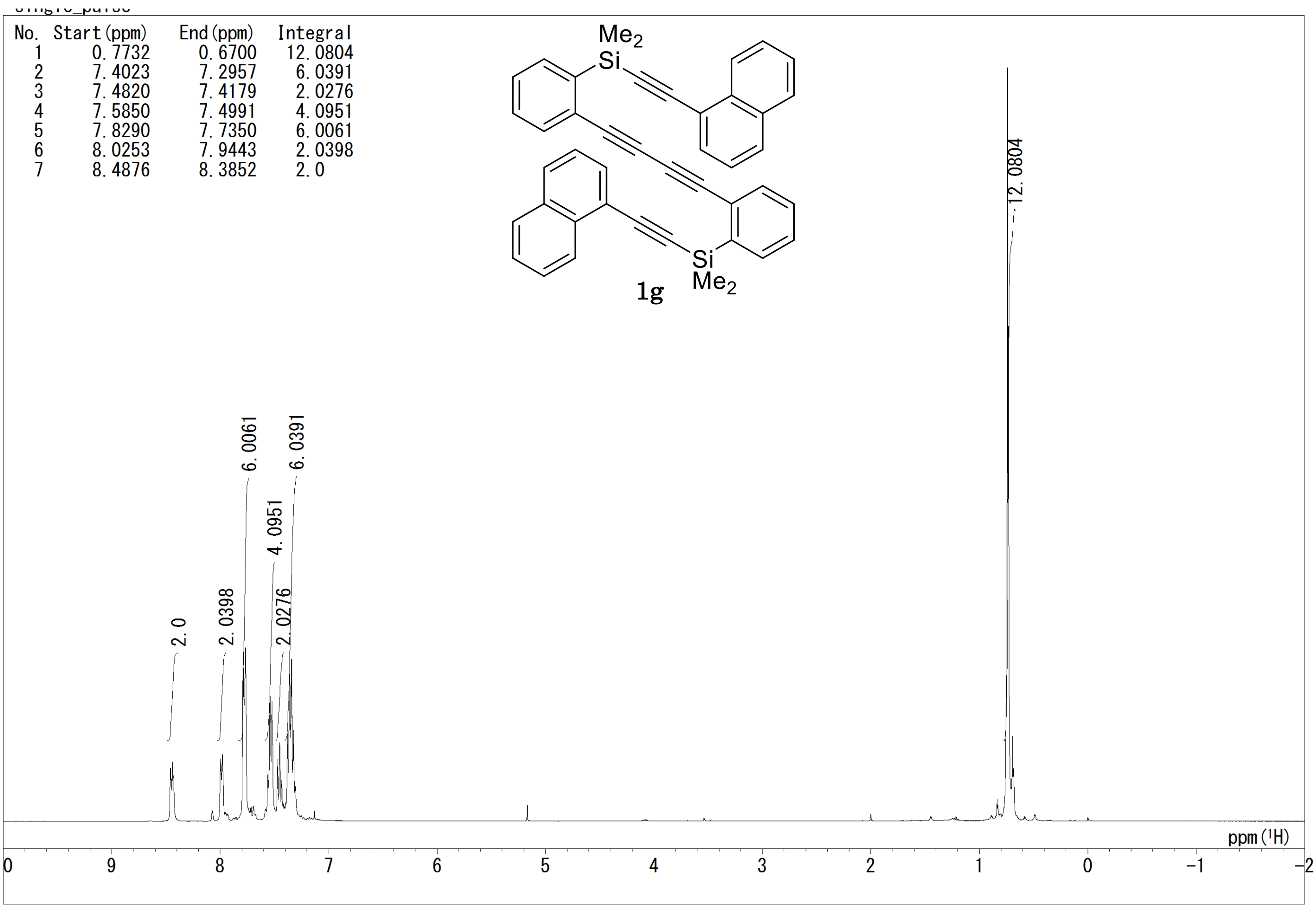

No.	Start (ppm)	End (ppm)	Integral
1	0.7732	0.6700	12.0804
2	7.4023	7.2957	6.0391
3	7.4820	7.4179	2.0276
4	7.5850	7.4991	4.0951
5	7.8290	7.7350	6.0061
6	8.0253	7.9443	2.0398
7	8.4876	8.3852	2.0

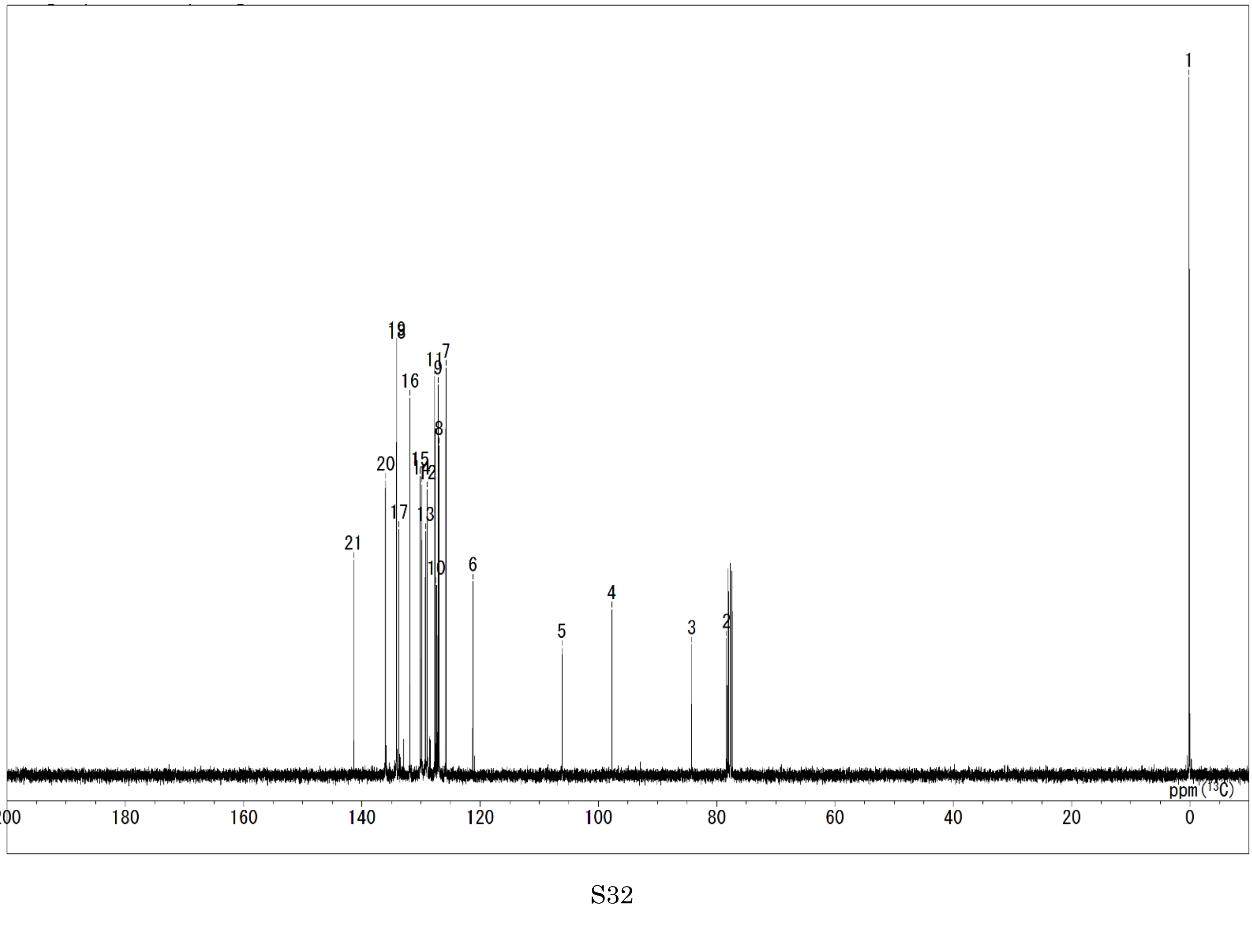

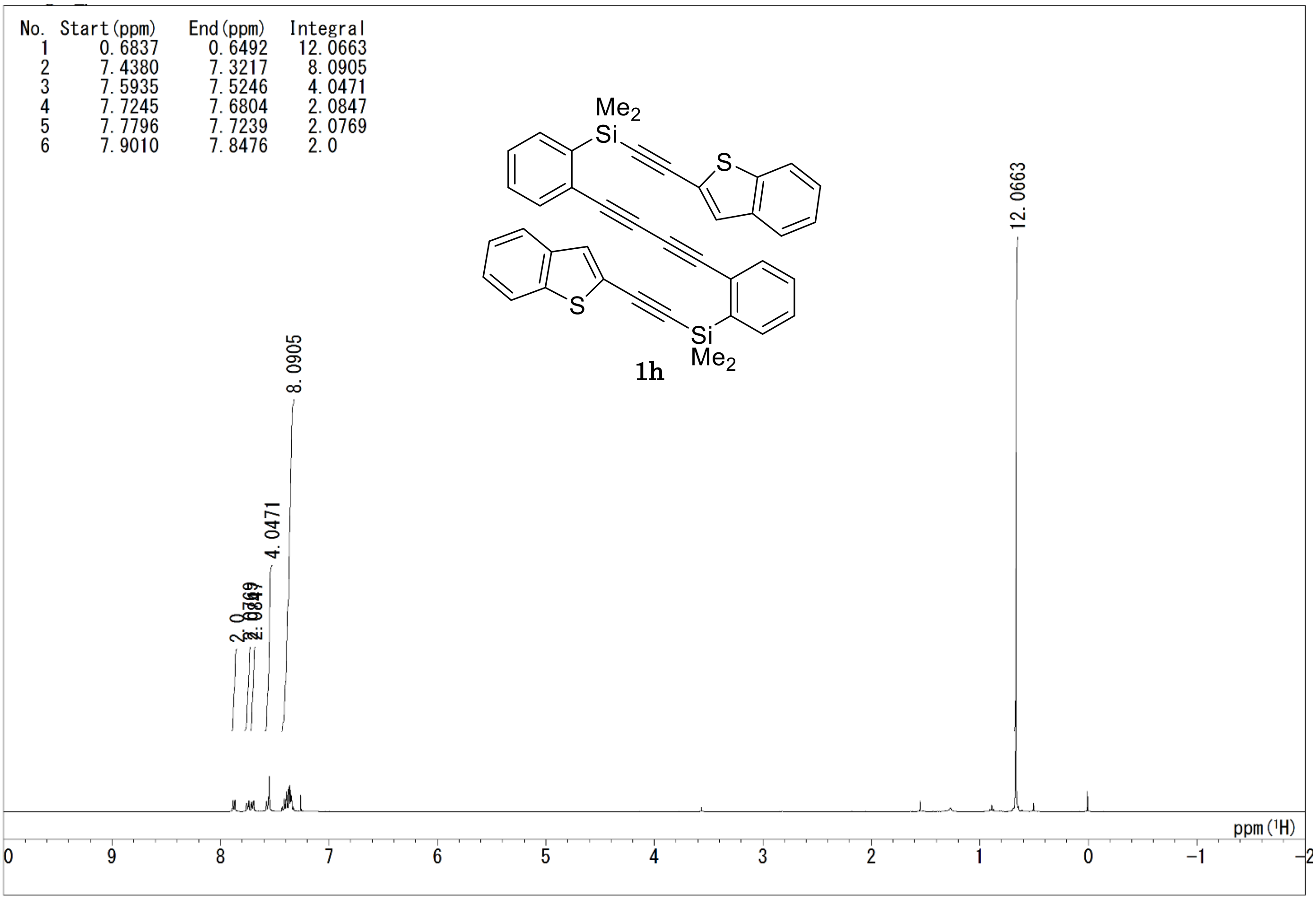

No.	Start(ppm)	End(ppm)	Integral
1	0.6837	0.6492	12.0663
2	7.4380	7.3217	8.0905
3	7.5935	7.5246	4.0471
4	7.7245	7.6804	2.0847
5	7.7796	7.7239	2.0769
6	7.9010	7.8476	2.0

Me2
Si
S
1h
12.0663
8.0905
4.0471
ppm (1H)
10
9
8
7
6
5
4
3
2
1
0
−1
−2

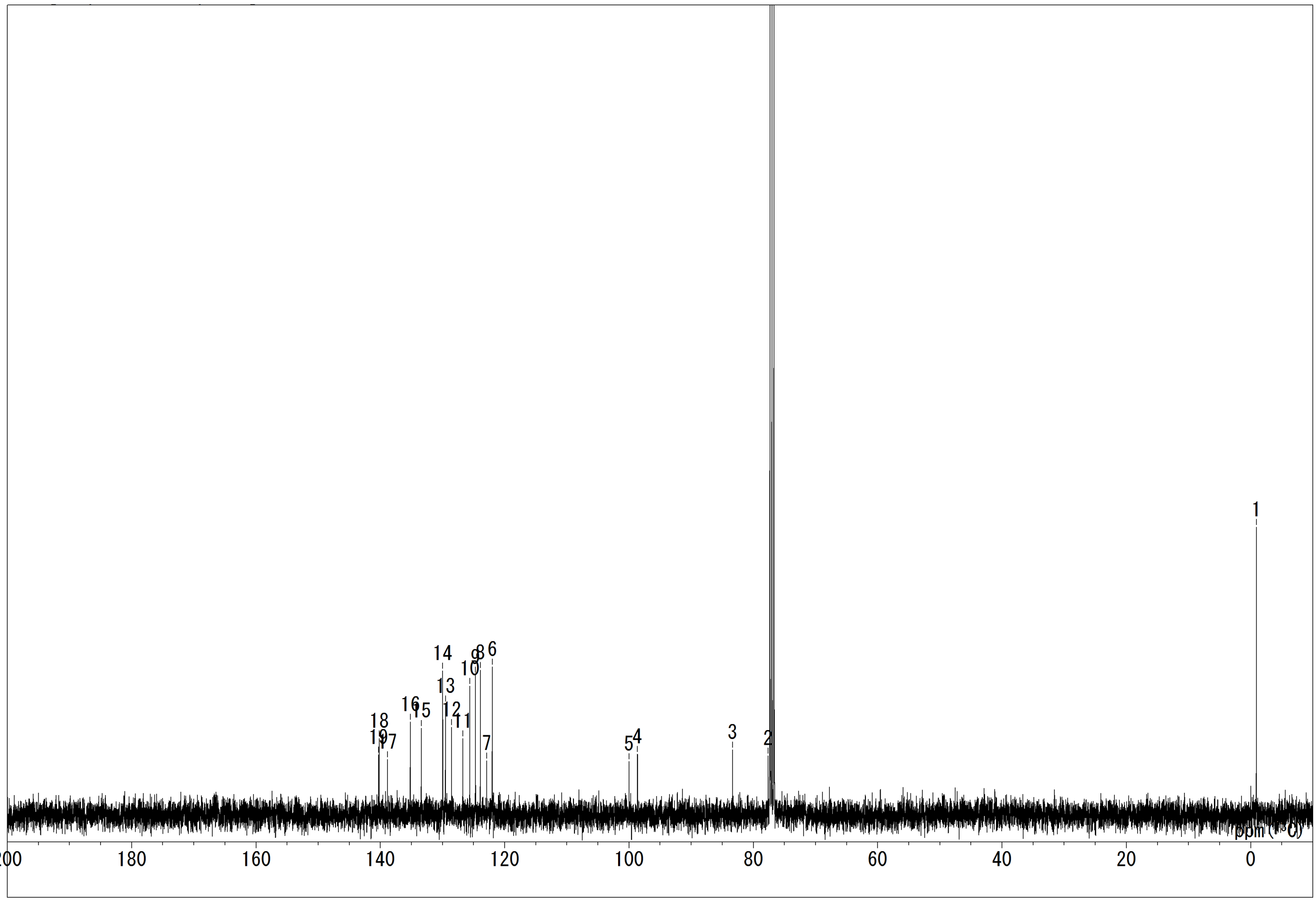

200
180
160
140
120
100
80
60
40
20
0

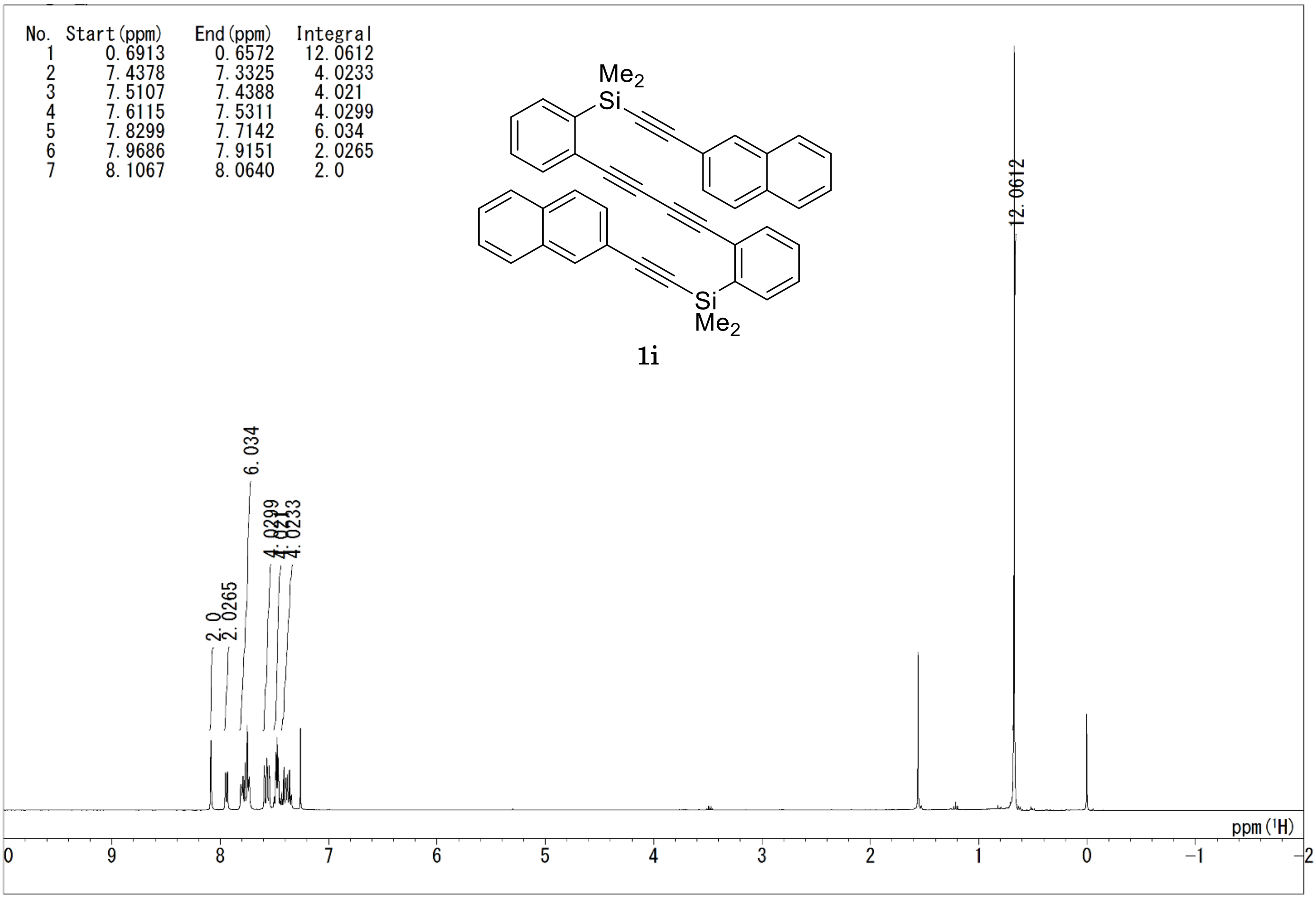
No. Start(ppm) End(ppm) Integral
1 0.6913 0.6572 12.0612
2 7.4378 7.3325 4.0233
3 7.5107 7.4388 4.021
4 7.6115 7.5311 4.0299
5 7.8299 7.7142 6.034
6 7.9686 7.9151 2.0265
7 8.1067 8.0640 2.0
Me2
Si
Si
Me2
1i
12.0612
6.034
4.0299
4.021
4.0233
2.0
2.0265
ppm (¹H)
10
9
8
7
6
5
4
3
2
1
0
−1
−2

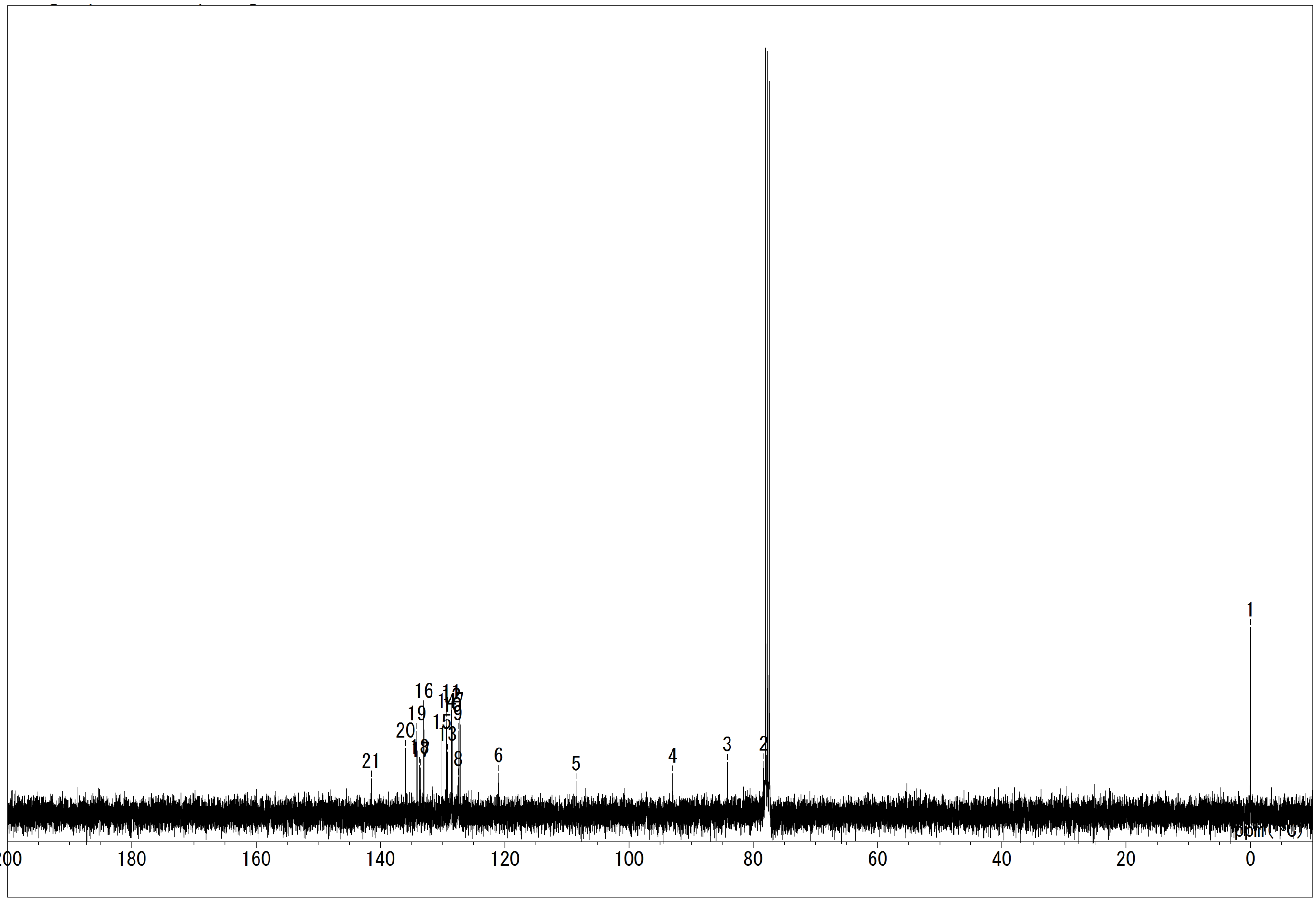
1
2
3
4
5
6
8
13
15
19
20
21
16
ppm (¹³C)
200
180
160
140
120
100
80
60
40
20
0

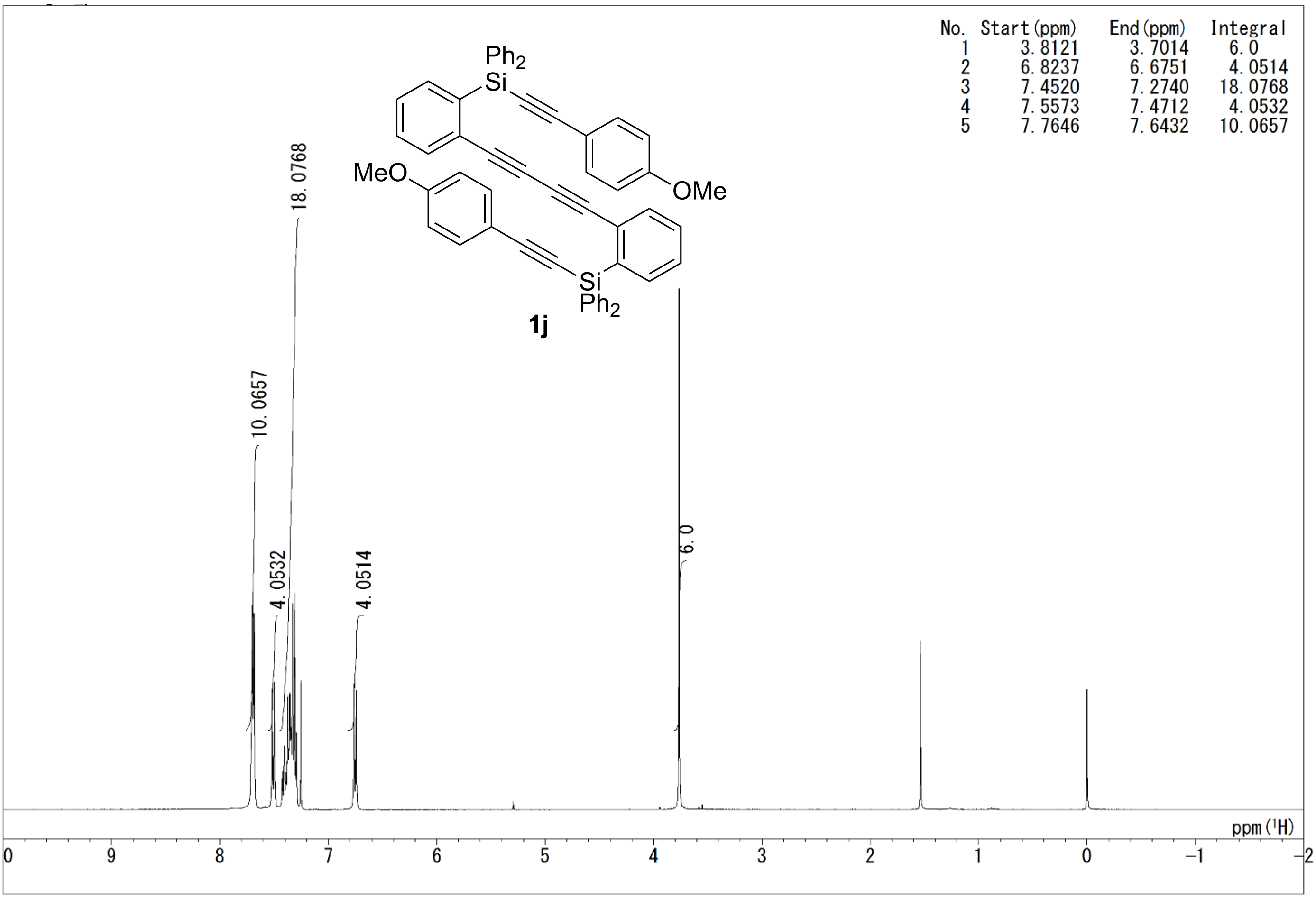
No. Start (ppm) End (ppm) Integral
1 3.8121 3.7014 6.0
2 6.8237 6.6751 4.0514
3 7.4520 7.2740 18.0768
4 7.5573 7.4712 4.0532
5 7.7646 7.6432 10.0657
Ph2
Si
MeO
OMe
Si
Ph2
1j
18.0768
10.0657
4.0532
4.0514
6.0
ppm (1H)
10 9 8 7 6 5 4 3 2 1 0 −1 −2

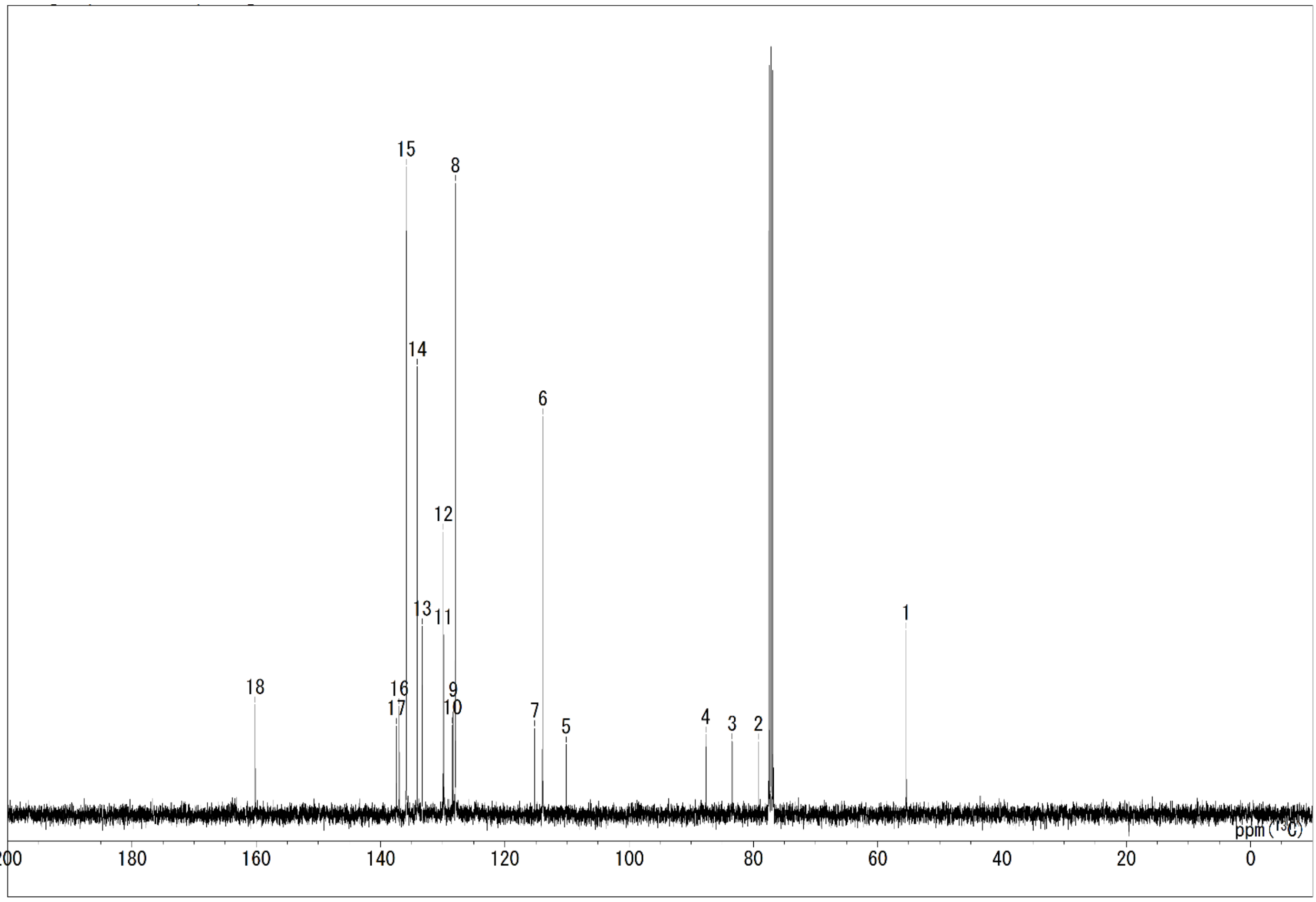
15
8
14
6
12
13
11
1
18
16
17
9
10
7
5
4
3
2
ppm (13C)
200 180 160 140 120 100 80 60 40 20 0

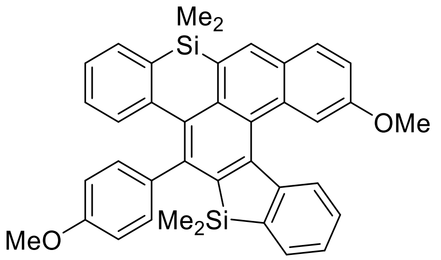

2a

No.	Start(ppm)	End(ppm)	Integral
1	-0.1613	-0.3692	3.0507
2	0.4046	0.2166	3.0074
3	0.6974	0.5994	3.0328
4	0.8101	0.6974	3.0068
5	3.7440	3.6890	3.0224
6	3.9121	3.8239	3.0791
7	6.9524	6.8344	3.0004
8	7.1002	7.0204	1.0142
9	7.1903	7.1002	4.0233
10	7.2964	7.1978	2.0467
11	7.5816	7.5137	1.0689
12	7.6470	7.5816	1.0289
13	7.7727	7.7039	1.0041
14	7.9141	7.8287	1.0
15	8.0959	8.0133	1.0113
16	8.1990	8.1208	1.0614

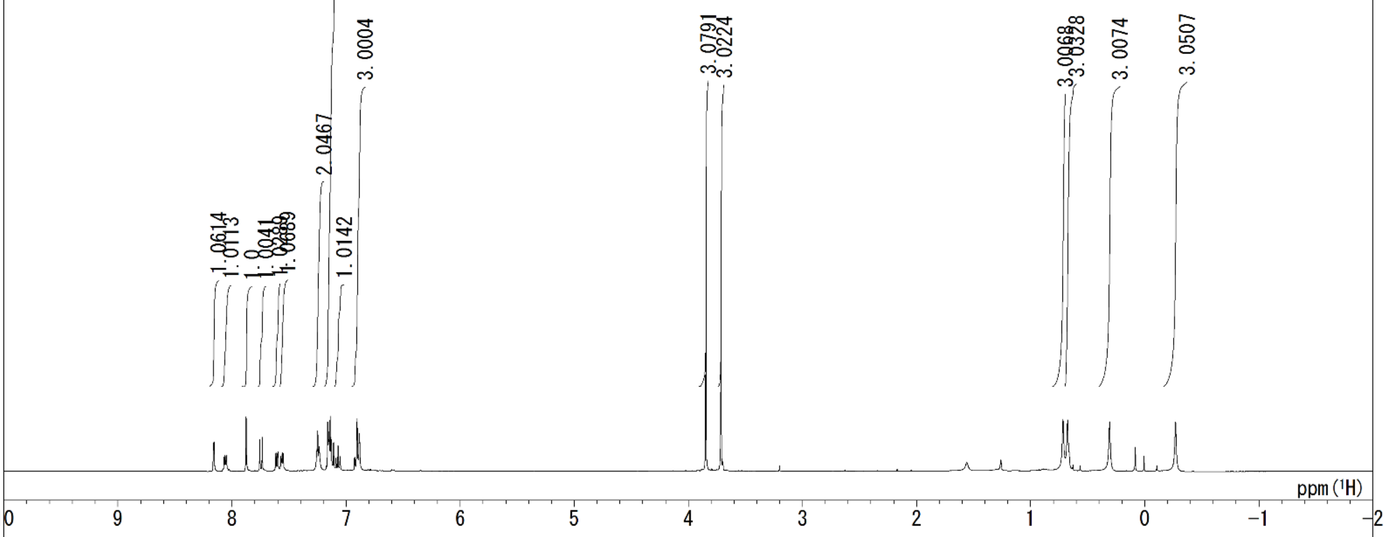

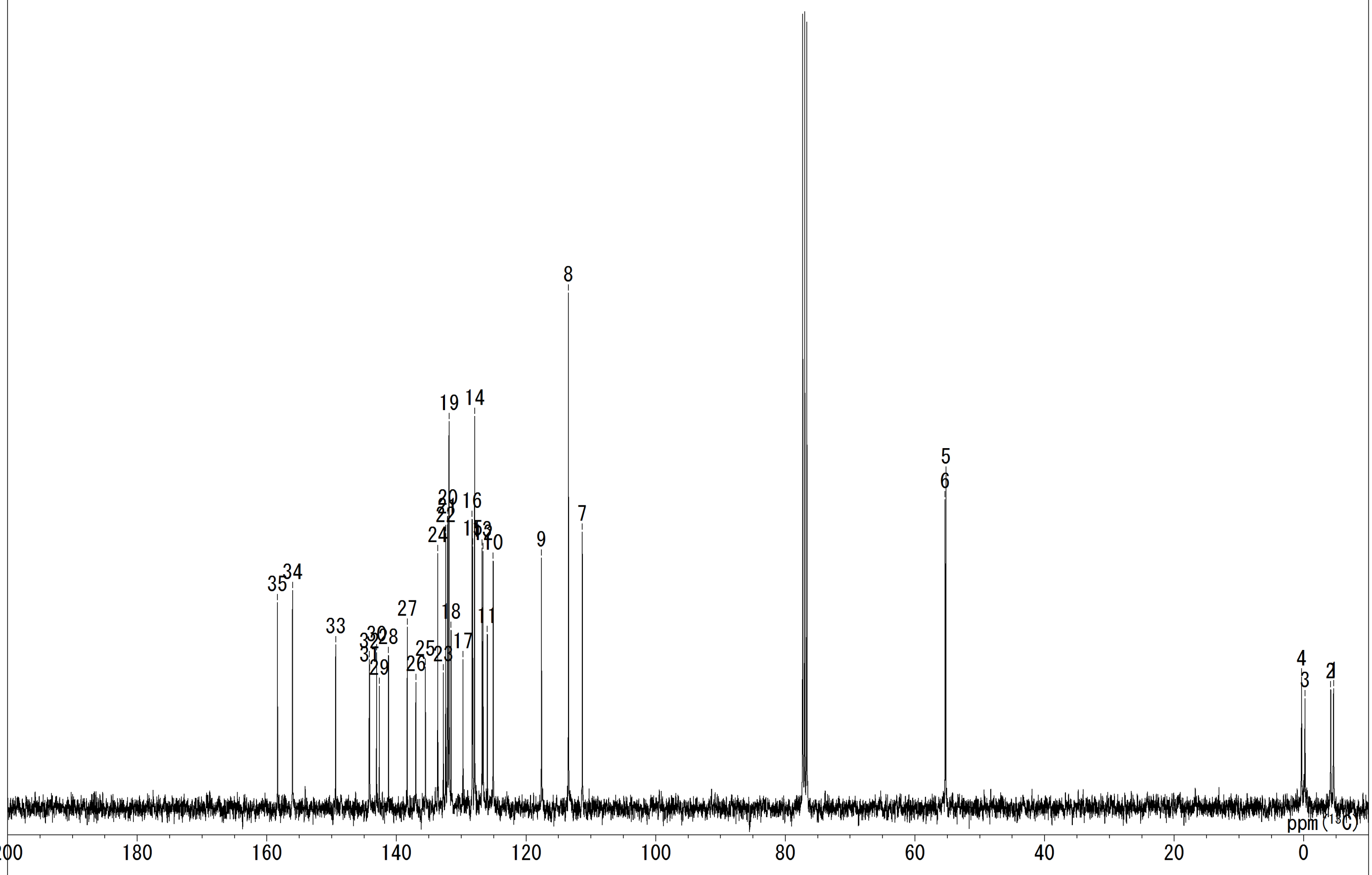

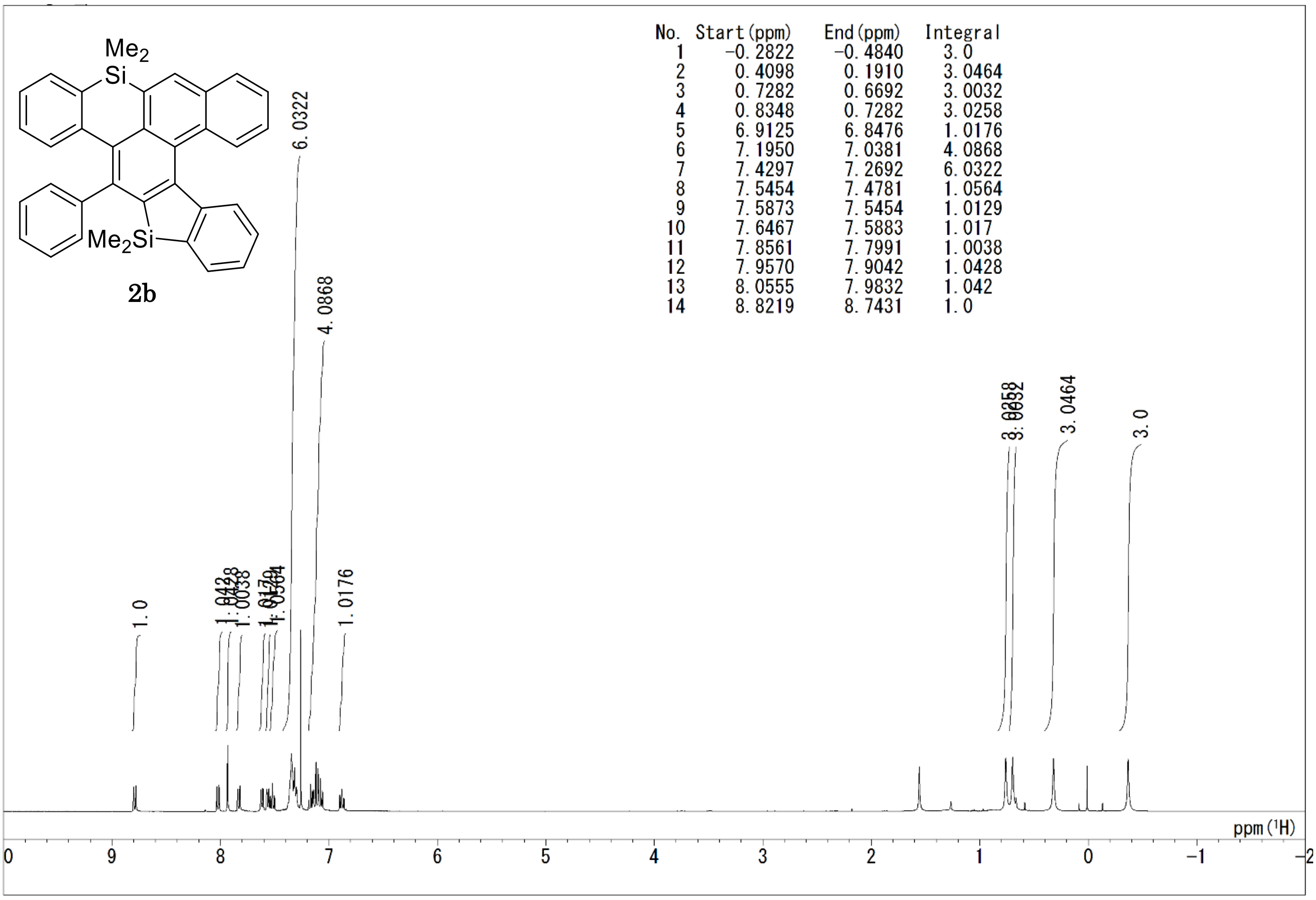

2b

No.	Start (ppm)	End (ppm)	Integral
1	-0.2822	-0.4840	3.0
2	0.4098	0.1910	3.0464
3	0.7282	0.6692	3.0032
4	0.8348	0.7282	3.0258
5	6.9125	6.8476	1.0176
6	7.1950	7.0381	4.0868
7	7.4297	7.2692	6.0322
8	7.5454	7.4781	1.0564
9	7.5873	7.5454	1.0129
10	7.6467	7.5883	1.017
11	7.8561	7.7991	1.0038
12	7.9570	7.9042	1.0428
13	8.0555	7.9832	1.042
14	8.8219	8.7431	1.0

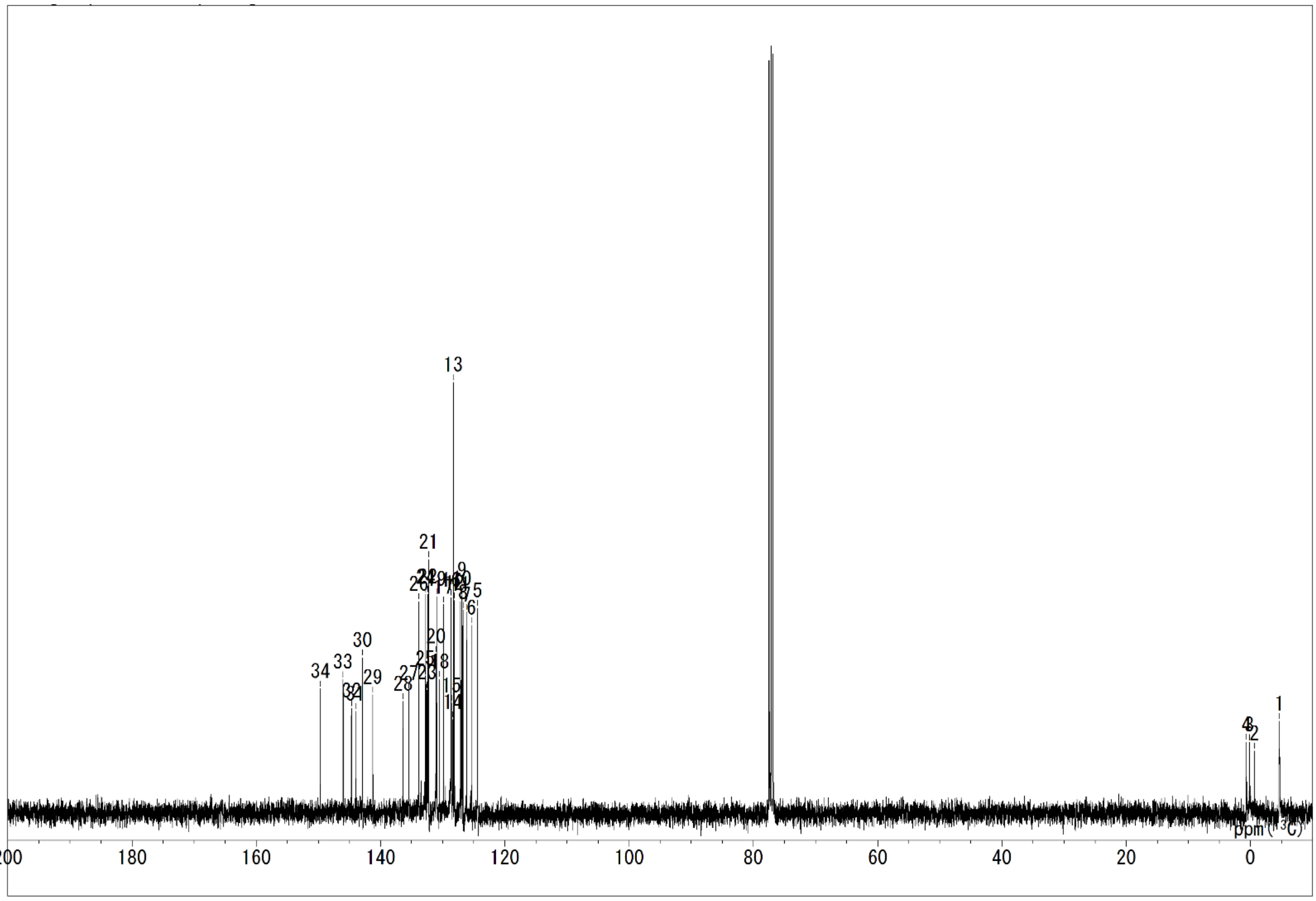

No.	Start (ppm)	End (ppm)	Integral
1	-0.1959	-0.3975	3.0314
2	0.4015	0.2149	3.0113
3	0.7028	0.5875	3.0039
4	0.8282	0.7028	3.0519
5	6.9532	6.8618	1.0153
6	7.1256	6.9766	4.0995
7	7.2196	7.1347	2.0999
8	7.3436	7.2248	3.3556
9	7.6641	7.5262	2.0761
10	7.8488	7.7542	1.0228
11	7.9329	7.8593	1.0072
12	8.0265	7.9442	1.0004
13	8.4624	8.3634	1.0

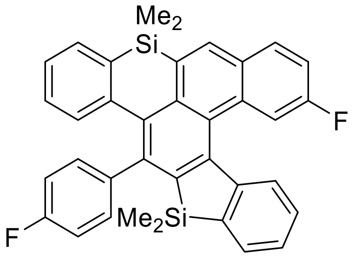

2c

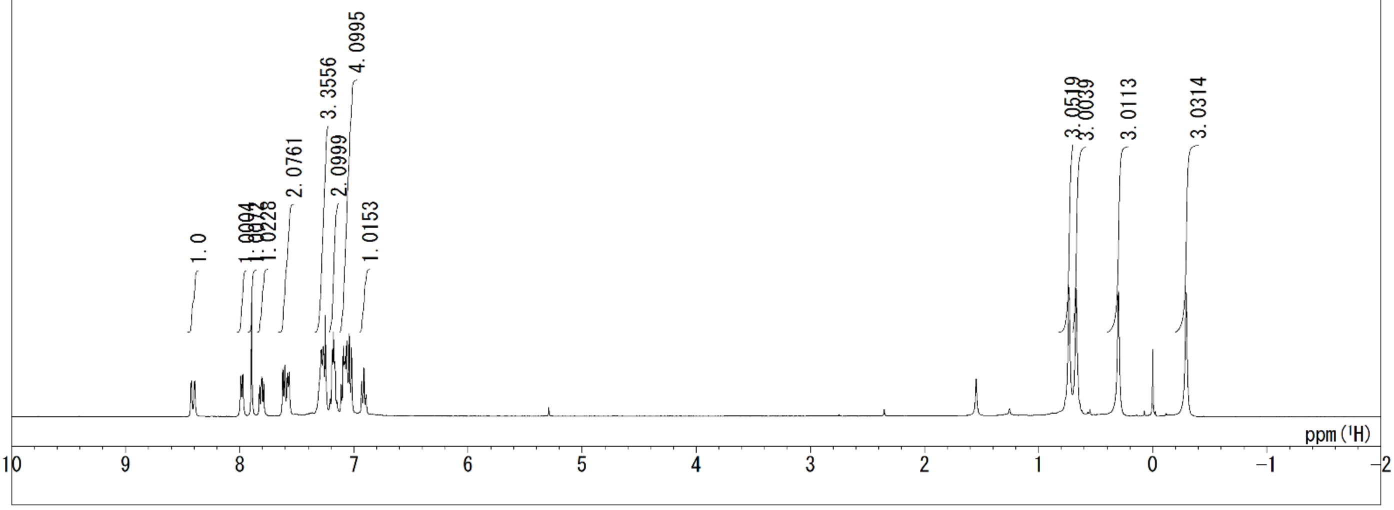

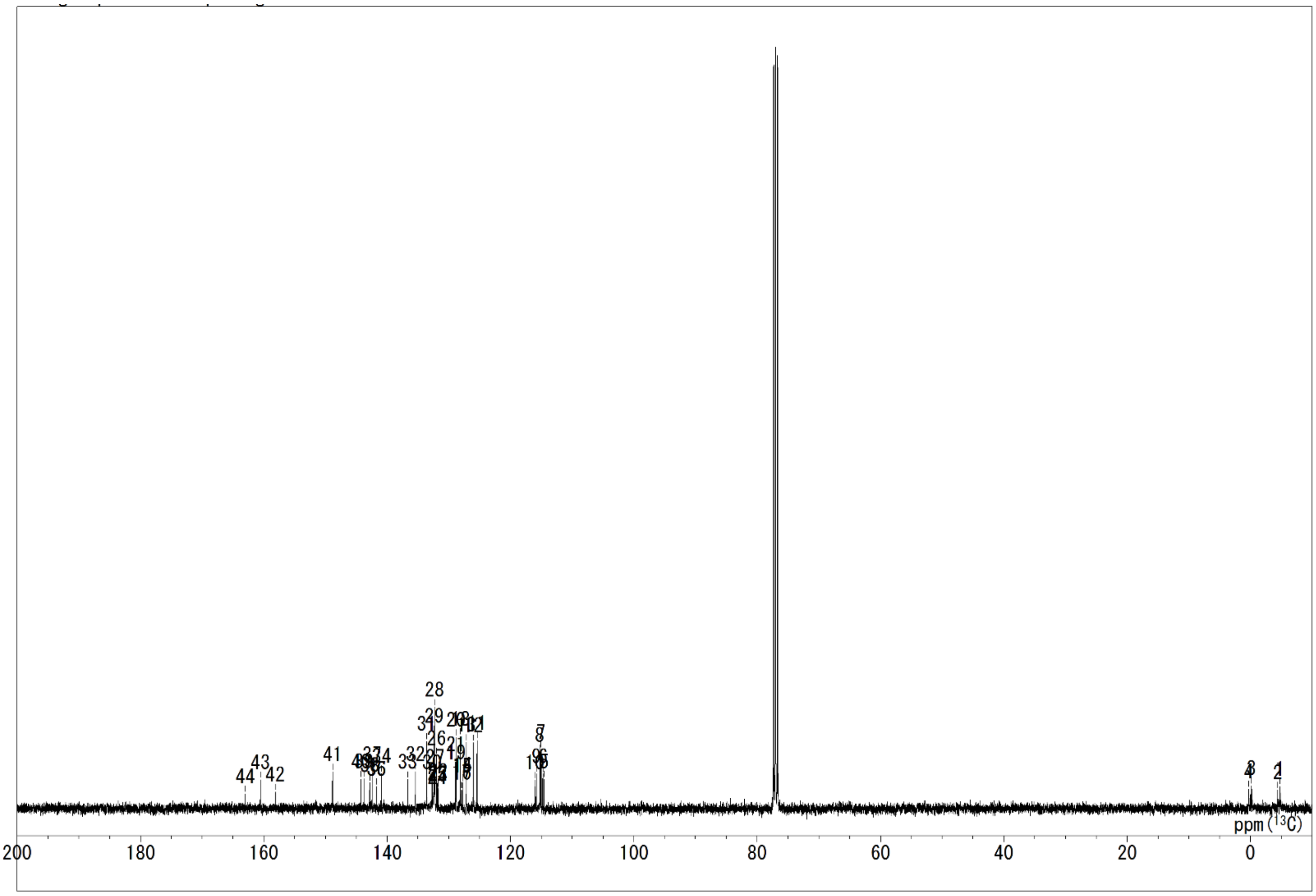

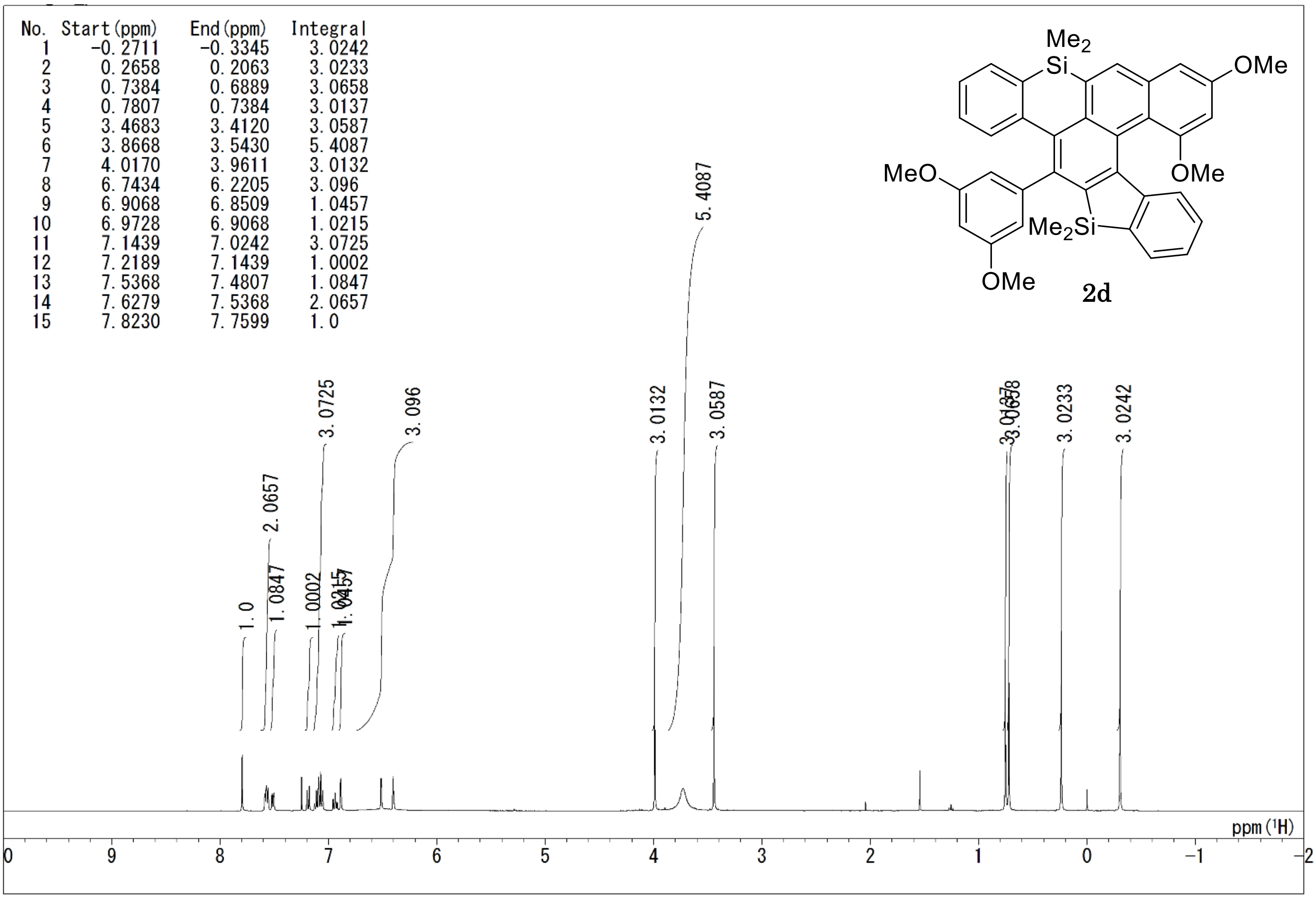

No.	Start (ppm)	End (ppm)	Integral
1	−0.2711	−0.3345	3.0242
2	0.2658	0.2063	3.0233
3	0.7384	0.6889	3.0658
4	0.7807	0.7384	3.0137
5	3.4683	3.4120	3.0587
6	3.8668	3.5430	5.4087
7	4.0170	3.9611	3.0132
8	6.7434	6.2205	3.096
9	6.9068	6.8509	1.0457
10	6.9728	6.9068	1.0215
11	7.1439	7.0242	3.0725
12	7.2189	7.1439	1.0002
13	7.5368	7.4807	1.0847
14	7.6279	7.5368	2.0657
15	7.8230	7.7599	1.0

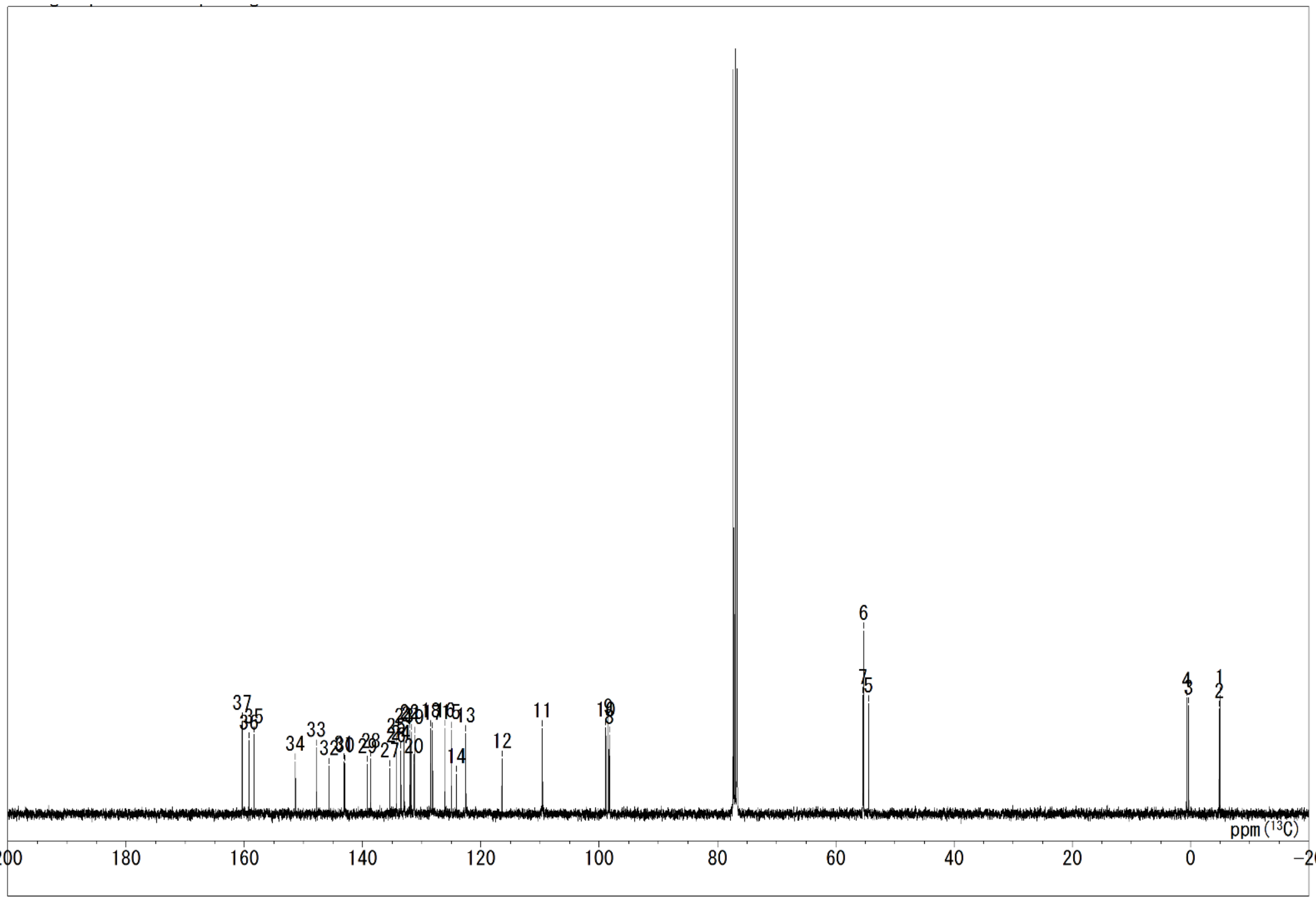

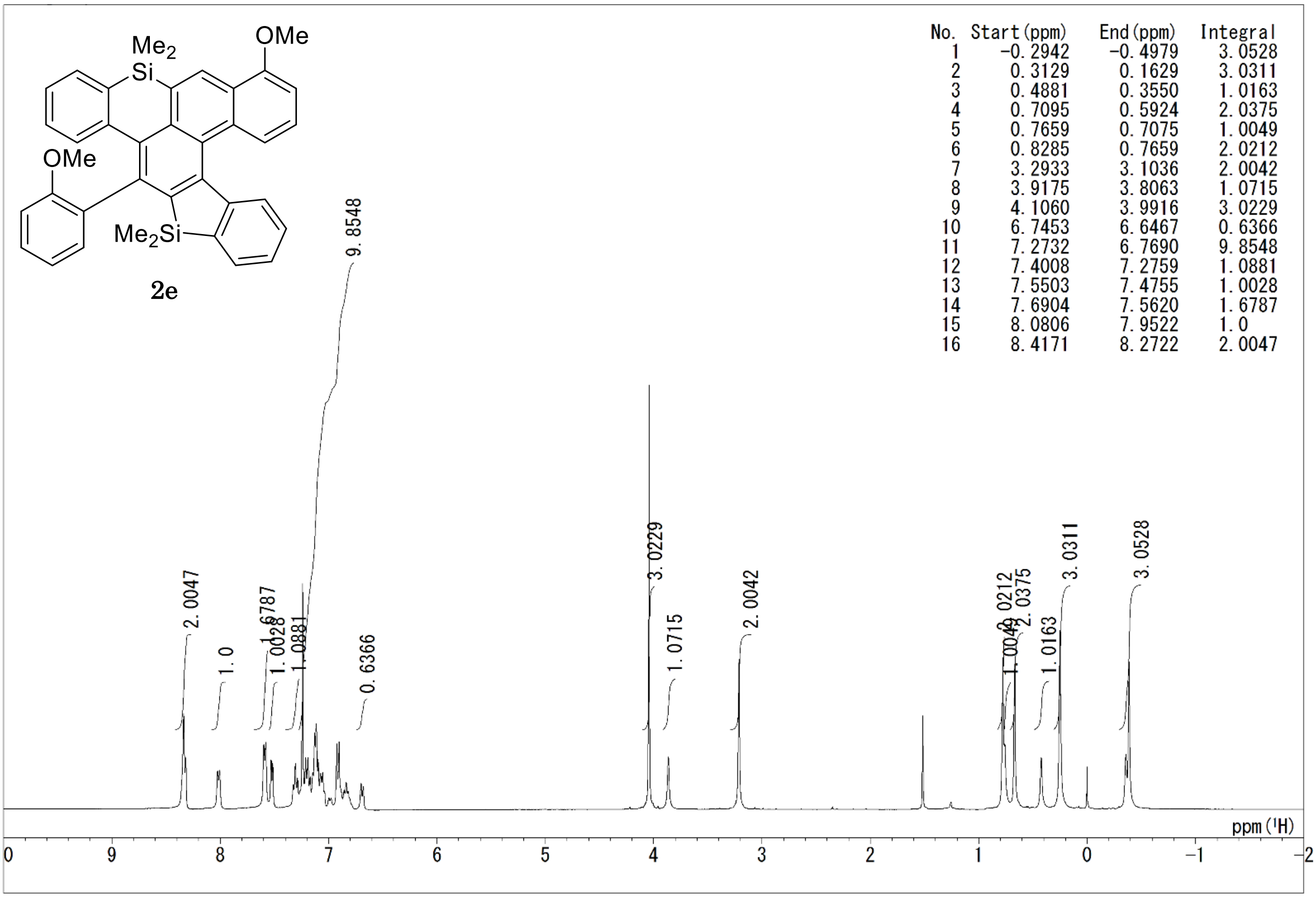

No.	Start (ppm)	End (ppm)	Integral
1	−0.2942	−0.4979	3.0528
2	0.3129	0.1629	3.0311
3	0.4881	0.3550	1.0163
4	0.7095	0.5924	2.0375
5	0.7659	0.7075	1.0049
6	0.8285	0.7659	2.0212
7	3.2933	3.1036	2.0042
8	3.9175	3.8063	1.0715
9	4.1060	3.9916	3.0229
10	6.7453	6.6467	0.6366
11	7.2732	6.7690	9.8548
12	7.4008	7.2759	1.0881
13	7.5503	7.4755	1.0028
14	7.6904	7.5620	1.6787
15	8.0806	7.9522	1.0
16	8.4171	8.2722	2.0047

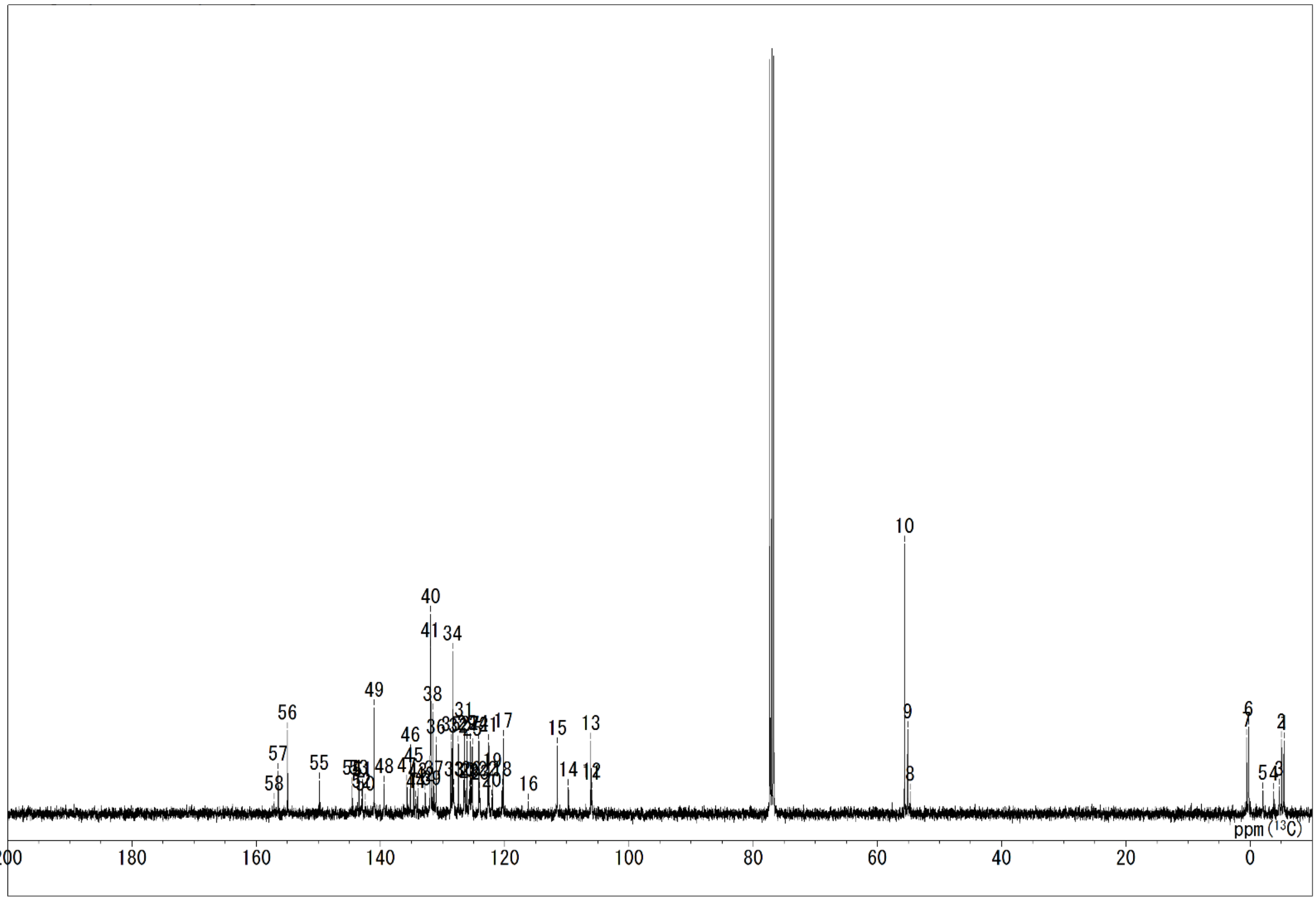

No.	Start(ppm)	End(ppm)	Integral
1	-0.2198	-0.4085	3.0572
2	0.4148	0.2764	3.0501
3	0.7327	0.6284	3.0546
4	0.8230	0.7327	3.0226
5	6.9323	6.8345	1.03
6	7.1218	7.0117	1.0066
7	7.2224	7.1248	3.0869
8	7.5226	7.2606	8.0645
9	7.6605	7.5262	6.0879
10	7.7347	7.6605	2.0483
11	7.8186	7.7418	1.007
12	7.9310	7.8448	1.0631
13	8.0172	7.9310	1.0184
14	8.2423	8.1369	1.0142
15	9.0395	8.9462	1.0

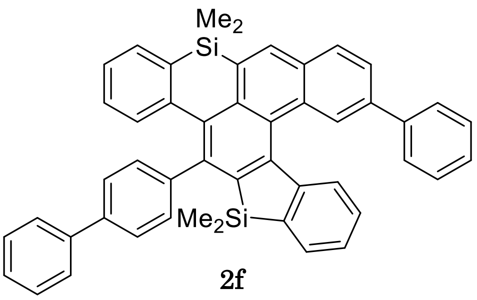

2f

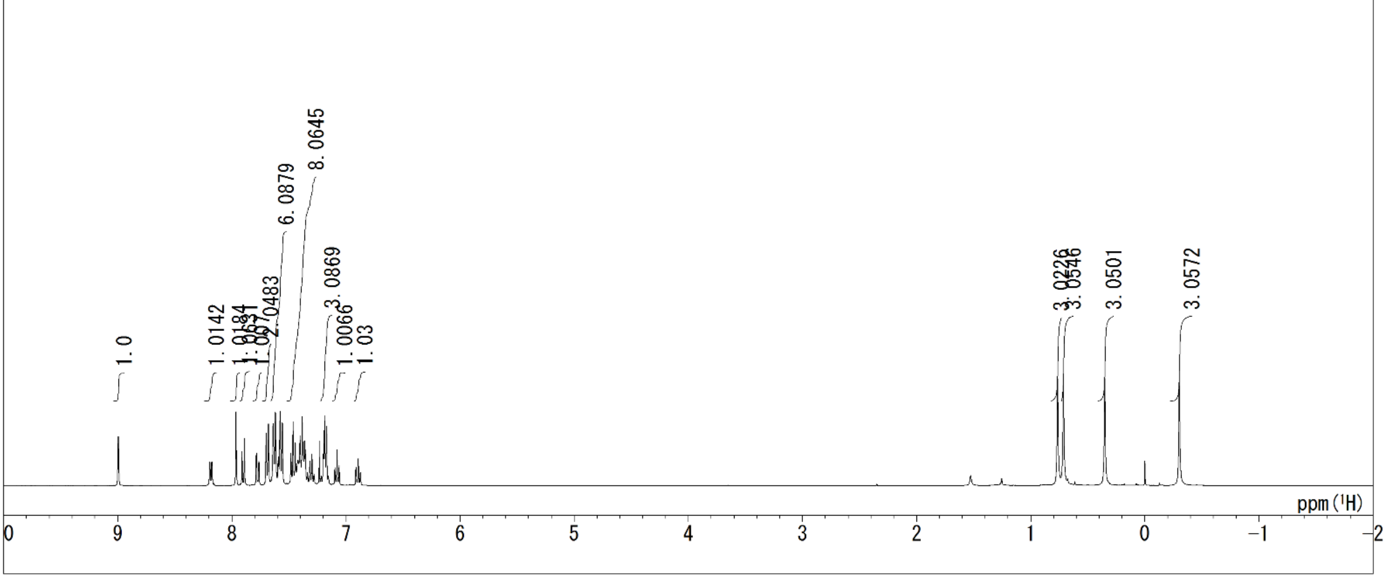

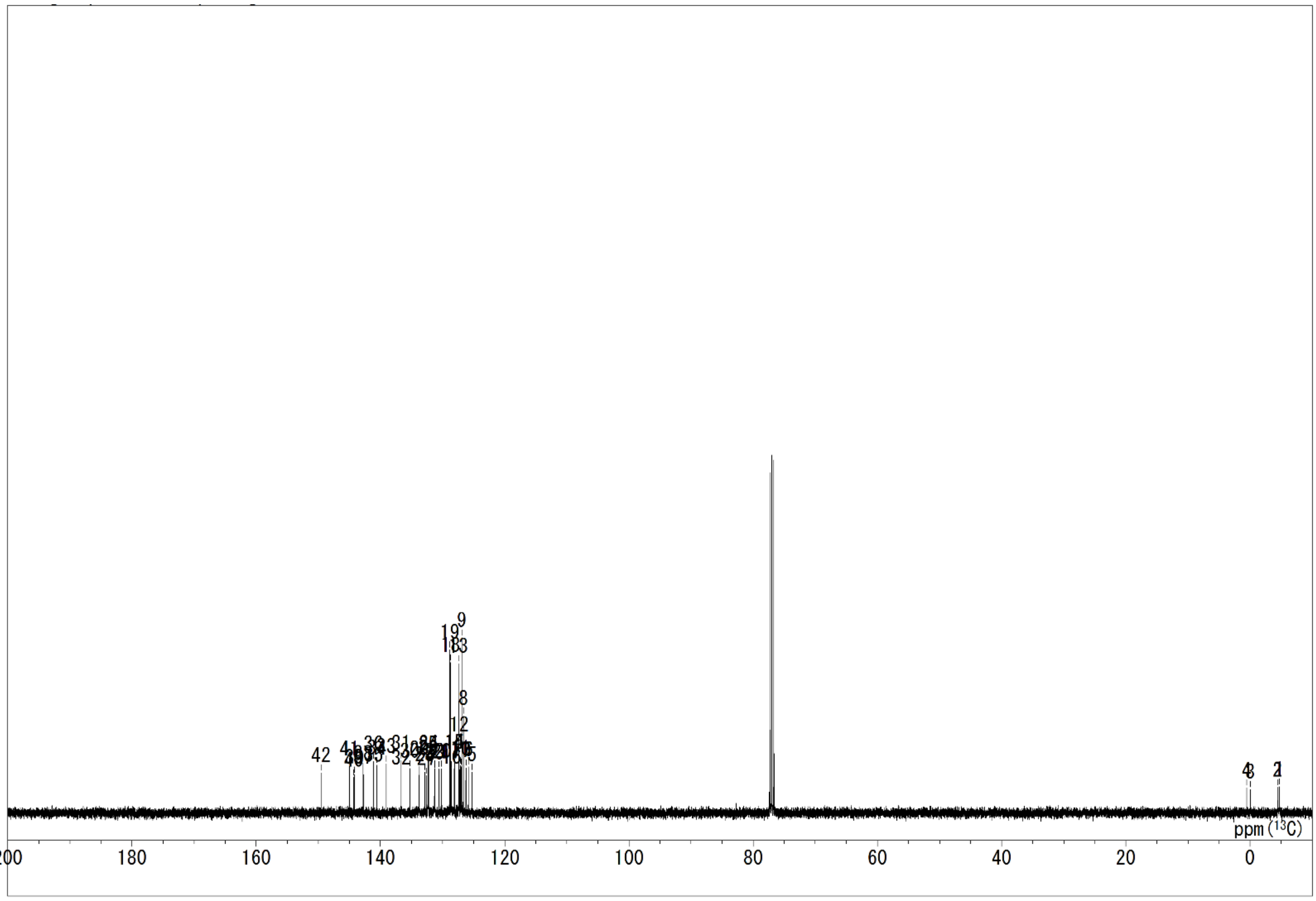

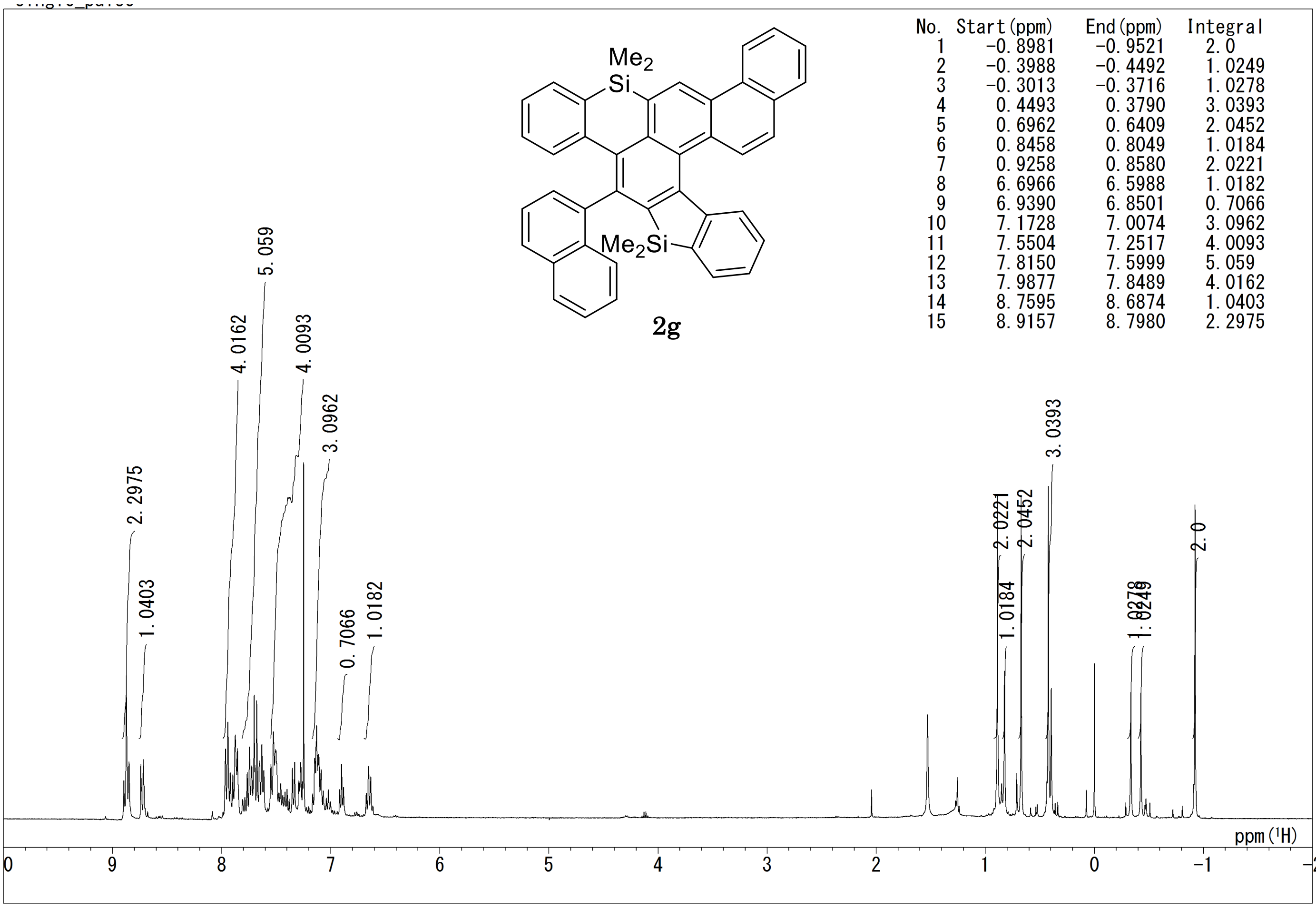

No.	Start (ppm)	End (ppm)	Integral
1	−0.8981	−0.9521	2.0
2	−0.3988	−0.4492	1.0249
3	−0.3013	−0.3716	1.0278
4	0.4493	0.3790	3.0393
5	0.6962	0.6409	2.0452
6	0.8458	0.8049	1.0184
7	0.9258	0.8580	2.0221
8	6.6966	6.5988	1.0182
9	6.9390	6.8501	0.7066
10	7.1728	7.0074	3.0962
11	7.5504	7.2517	4.0093
12	7.8150	7.5999	5.059
13	7.9877	7.8489	4.0162
14	8.7595	8.6874	1.0403
15	8.9157	8.7980	2.2975

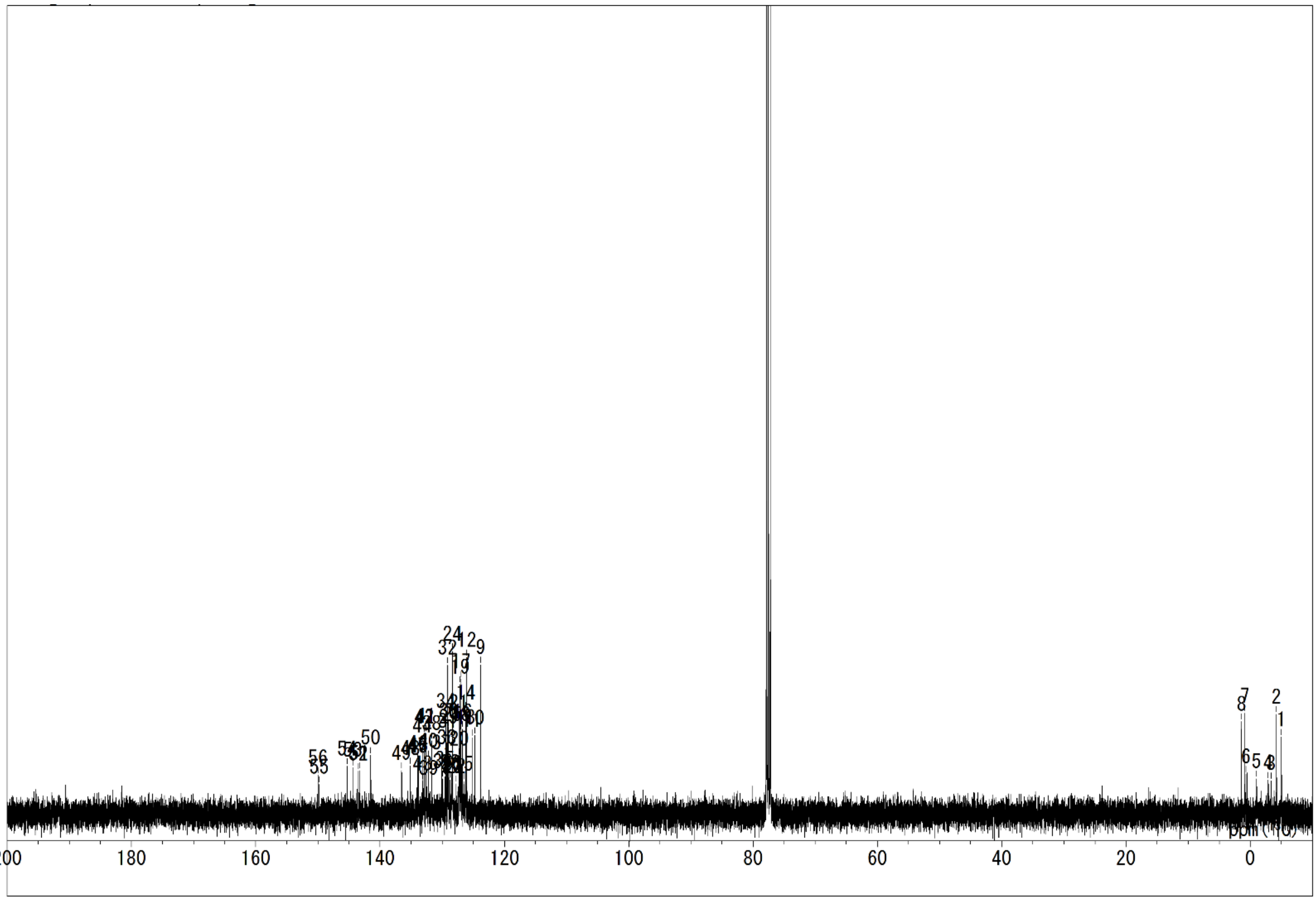

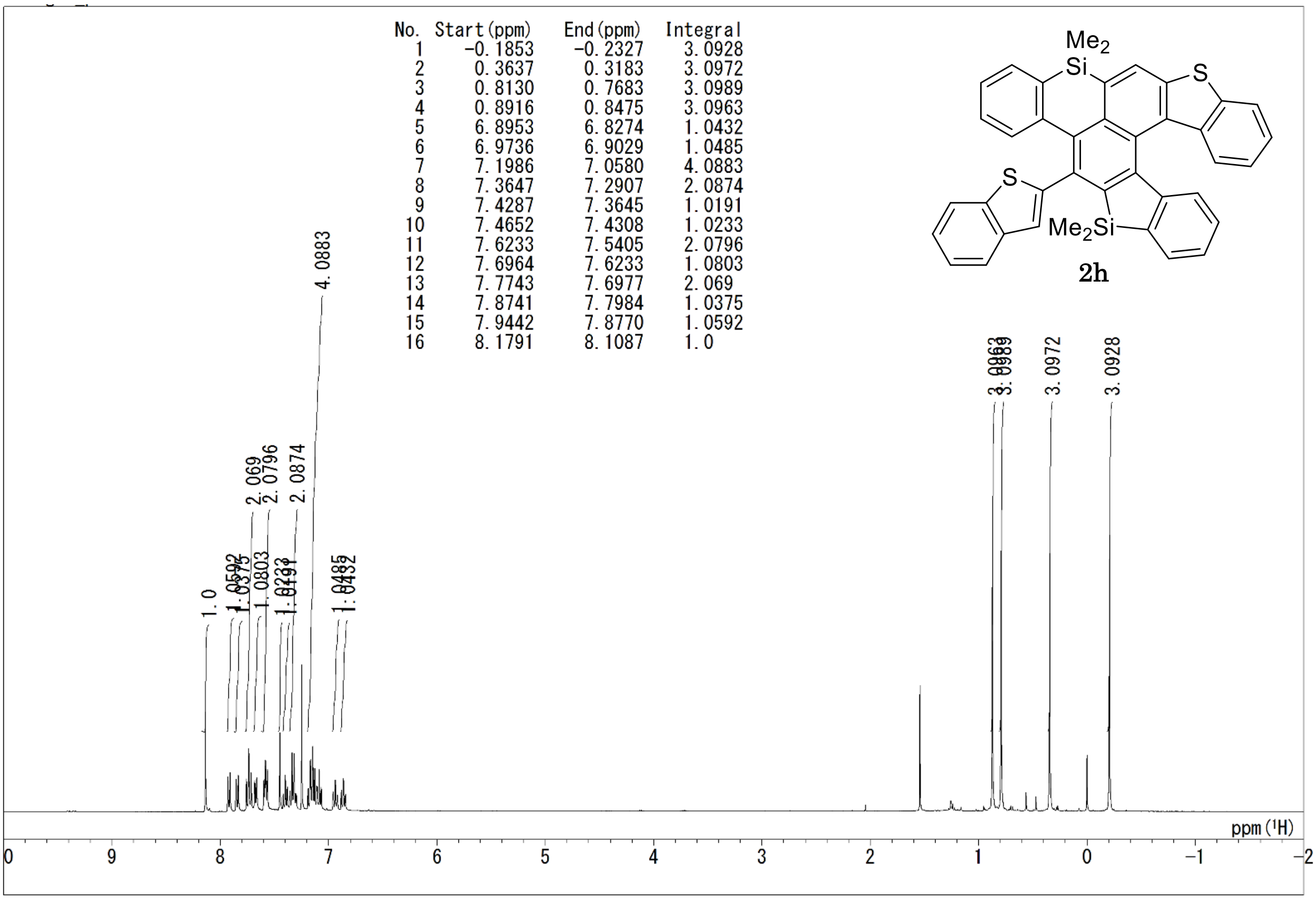

No.	Start (ppm)	End (ppm)	Integral
1	−0.1853	−0.2327	3.0928
2	0.3637	0.3183	3.0972
3	0.8130	0.7683	3.0989
4	0.8916	0.8475	3.0963
5	6.8953	6.8274	1.0432
6	6.9736	6.9029	1.0485
7	7.1986	7.0580	4.0883
8	7.3647	7.2907	2.0874
9	7.4287	7.3645	1.0191
10	7.4652	7.4308	1.0233
11	7.6233	7.5405	2.0796
12	7.6964	7.6233	1.0803
13	7.7743	7.6977	2.069
14	7.8741	7.7984	1.0375
15	7.9442	7.8770	1.0592
16	8.1791	8.1087	1.0

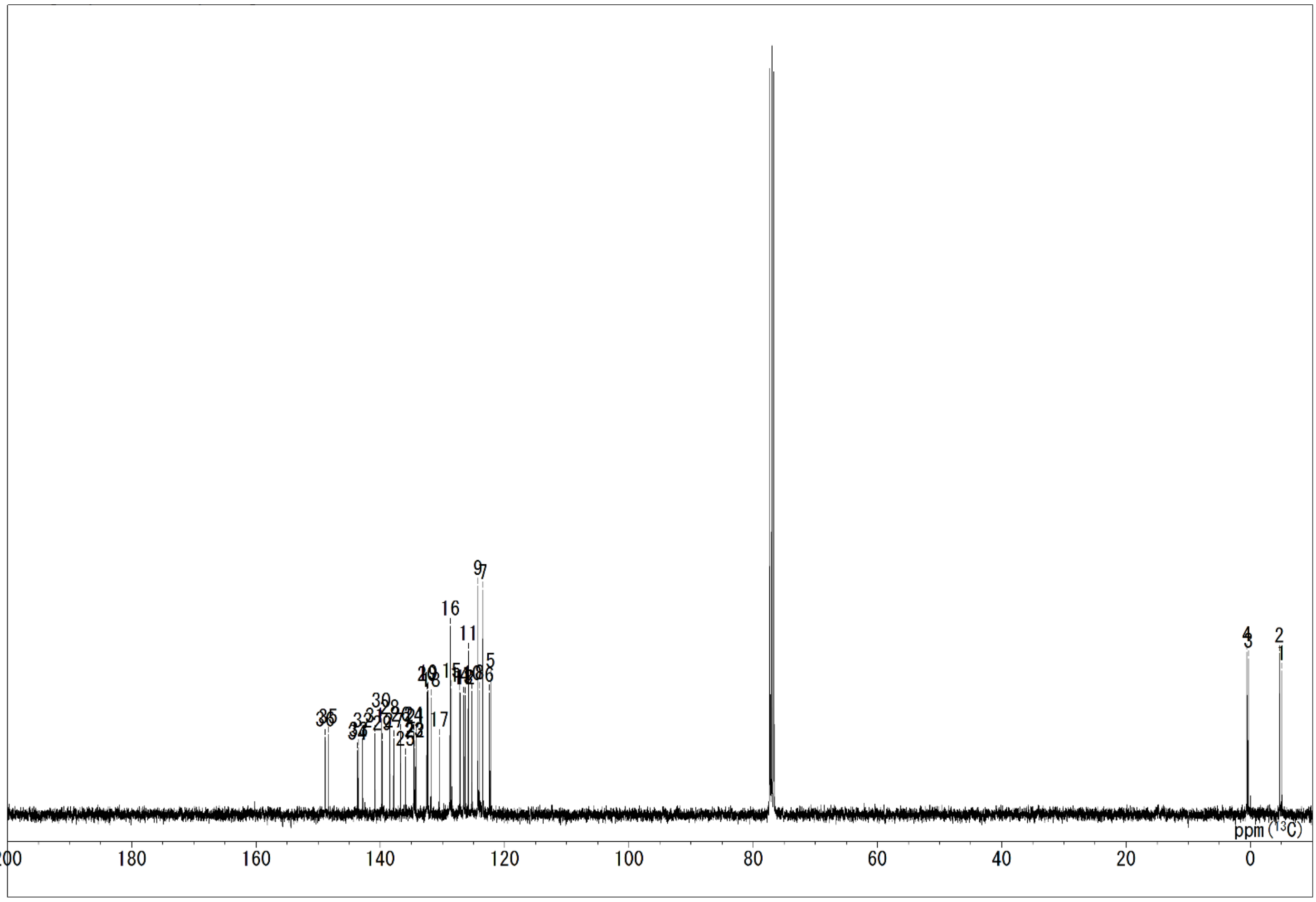

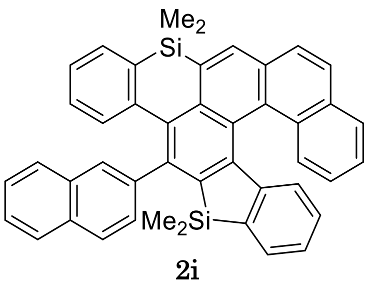

2i

No.	Start (ppm)	End (ppm)	Integral	No.	Start (ppm)	End (ppm)
1	-0.5085	-0.5686	3.0191	20	8.1237	8.0809
2	0.4165	0.3734	3.001	21	8.5944	8.5368
3	0.8377	0.8090	3.0279			
4	0.8636	0.8377	3.0105			
5	6.5960	6.5370	1.0097			
6	6.6638	6.6033	1.0			
7	6.8152	6.7561	1.0718			
8	6.9421	6.8902	1.0119			
9	7.0690	7.0128	1.0182			
10	7.2290	7.1802	1.0273			
11	7.2867	7.2320	2.3387			
12	7.4742	7.3367	3.0114			
13	7.5866	7.4986	2.0581			
14	7.6788	7.6284	1.0321			
15	7.8149	7.7537	1.068			
16	7.9038	7.8611	1.0089			
17	7.9657	7.9038	3.0015			
18	8.0134	7.9657	1.0573			
19	8.0803	8.0610	1.0112			

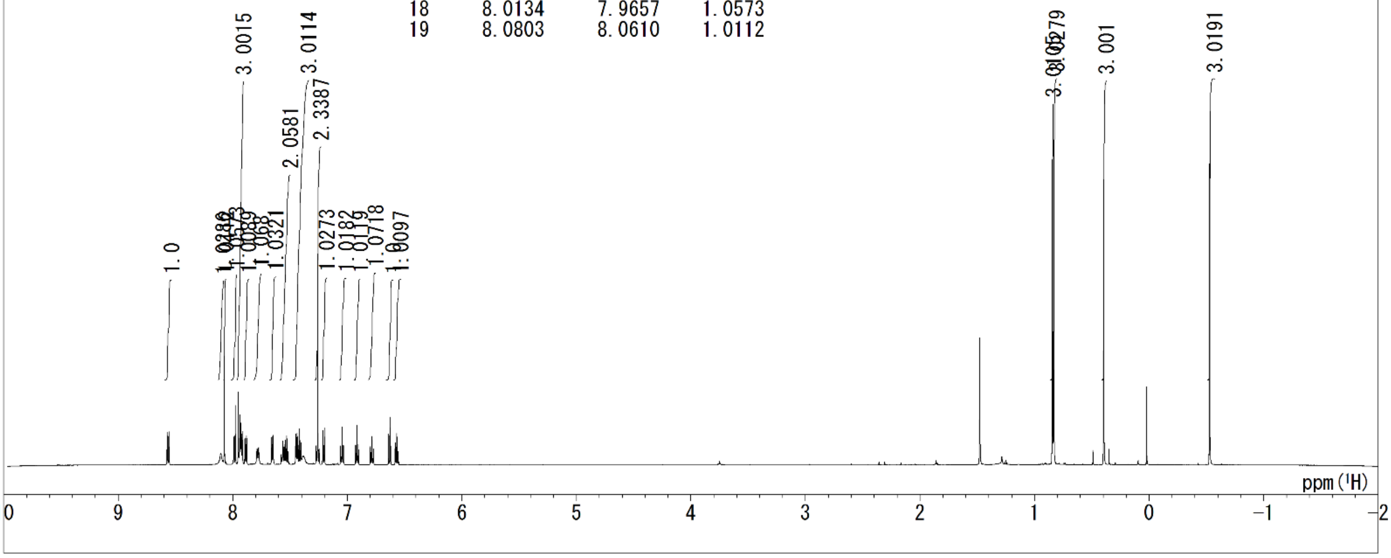

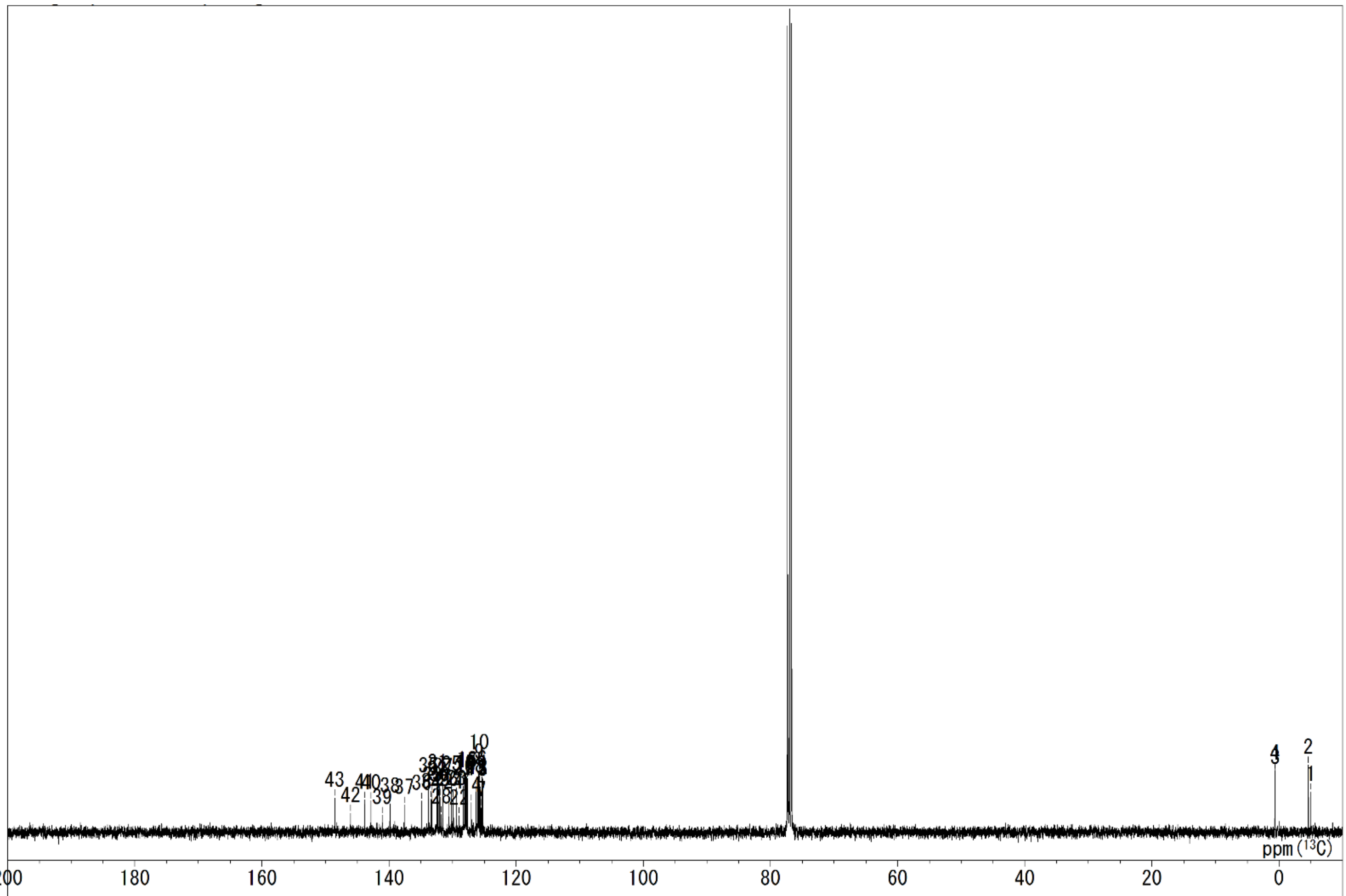

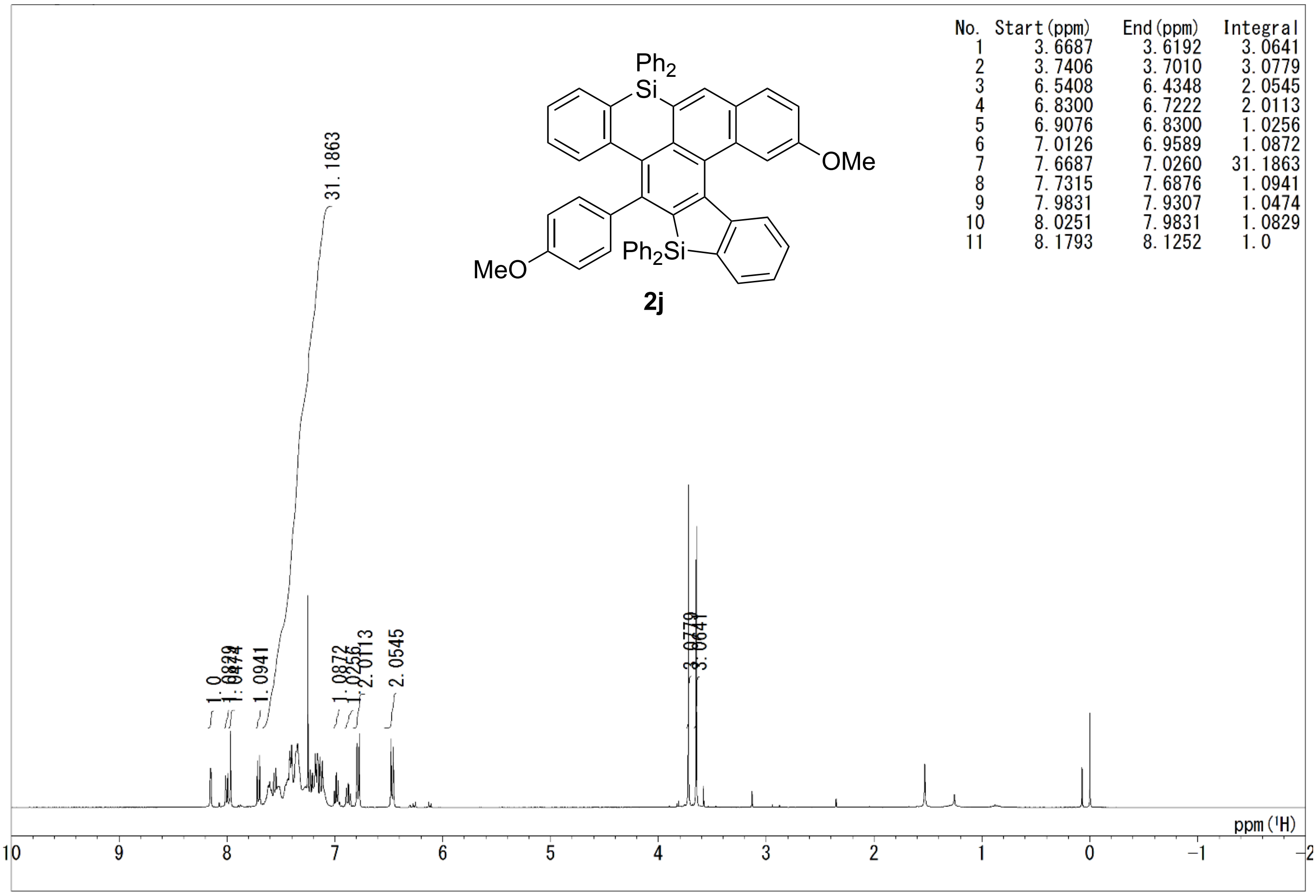
No. Start(ppm) End(ppm) Integral
1 3.6687 3.6192 3.0641
2 3.7406 3.7010 3.0779
3 6.5408 6.4348 2.0545
4 6.8300 6.7222 2.0113
5 6.9076 6.8300 1.0256
6 7.0126 6.9589 1.0872
7 7.6687 7.0260 31.1863
8 7.7315 7.6876 1.0941
9 7.9831 7.9307 1.0474
10 8.0251 7.9831 1.0829
11 8.1793 8.1252 1.0
Ph2
Si
OMe
MeO
Ph2Si
2j
ppm (1H)

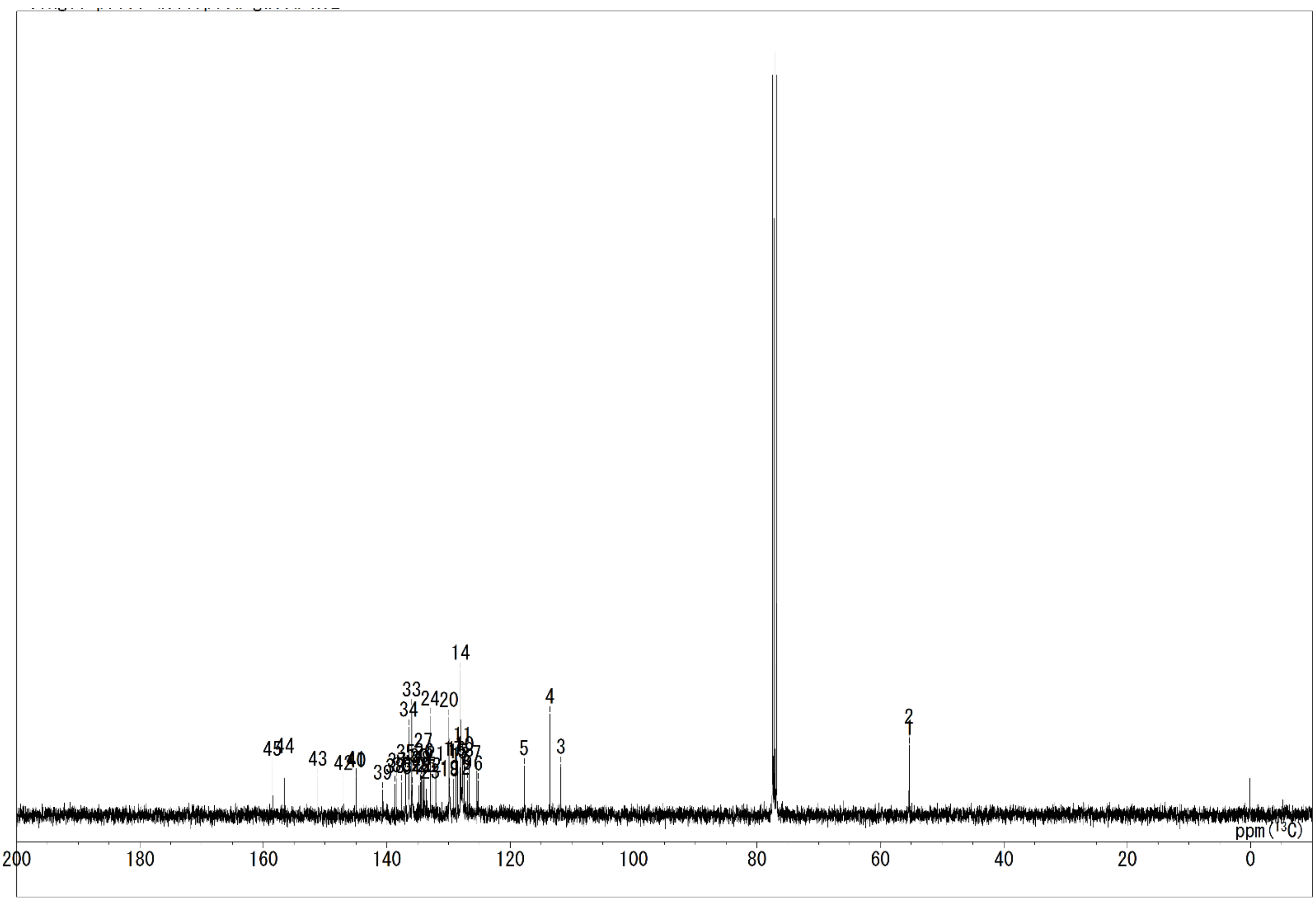
ppm (13C)

No.	Start (ppm)	End (ppm)	Integral
1	−0.3881	−0.4637	6.0358
2	−0.2689	−0.3883	6.0367
3	0.1373	0.0777	6.0972
4	0.4338	0.3197	6.0772
5	3.7664	3.7197	6.0209
6	3.8032	3.7664	6.0891
7	6.1367	6.0335	2.0
8	6.4666	6.3904	2.0271
9	6.5645	6.4988	2.0829
10	6.7268	6.6641	4.0124
11	6.7812	6.7268	2.0914
12	6.8901	6.7913	4.0874
13	6.9466	6.8901	2.0663
14	7.0842	7.0226	2.0595
15	7.1477	7.0842	4.0706
16	7.3767	7.3246	2.0049
17	7.4729	7.4189	2.091
18	7.5611	7.4839	4.0508

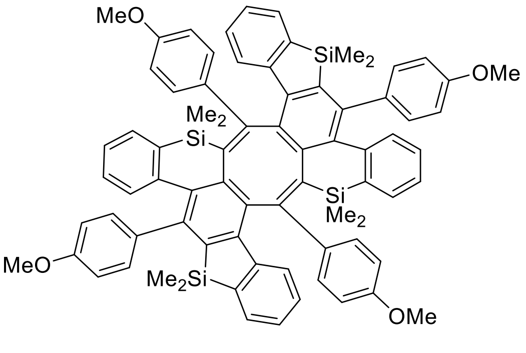

3a

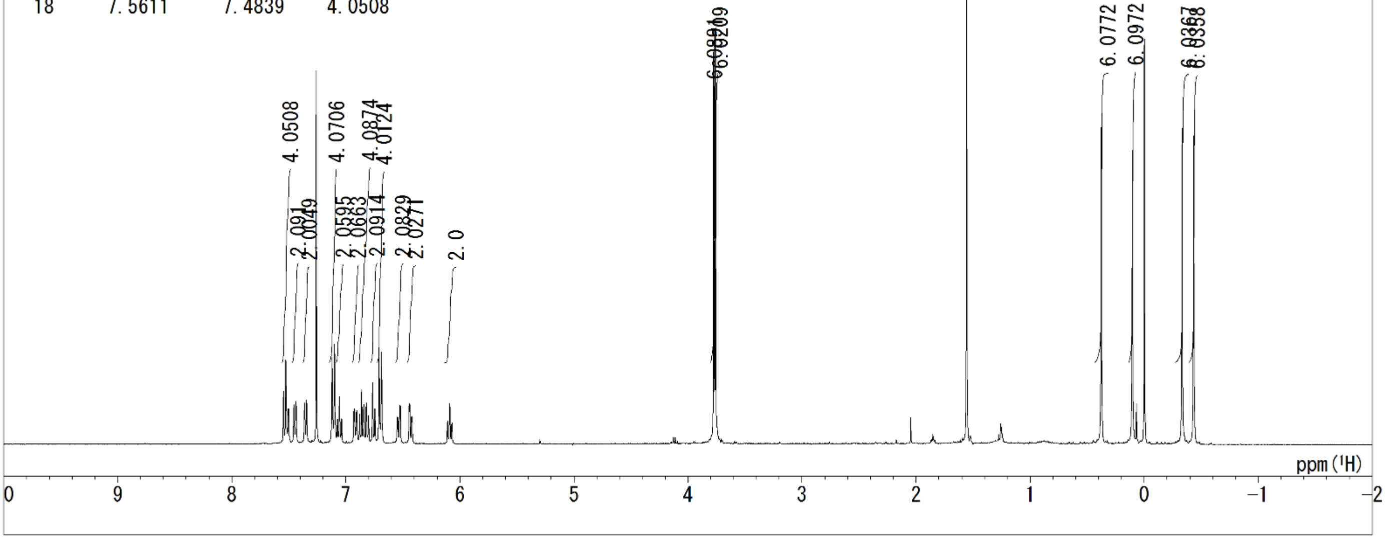

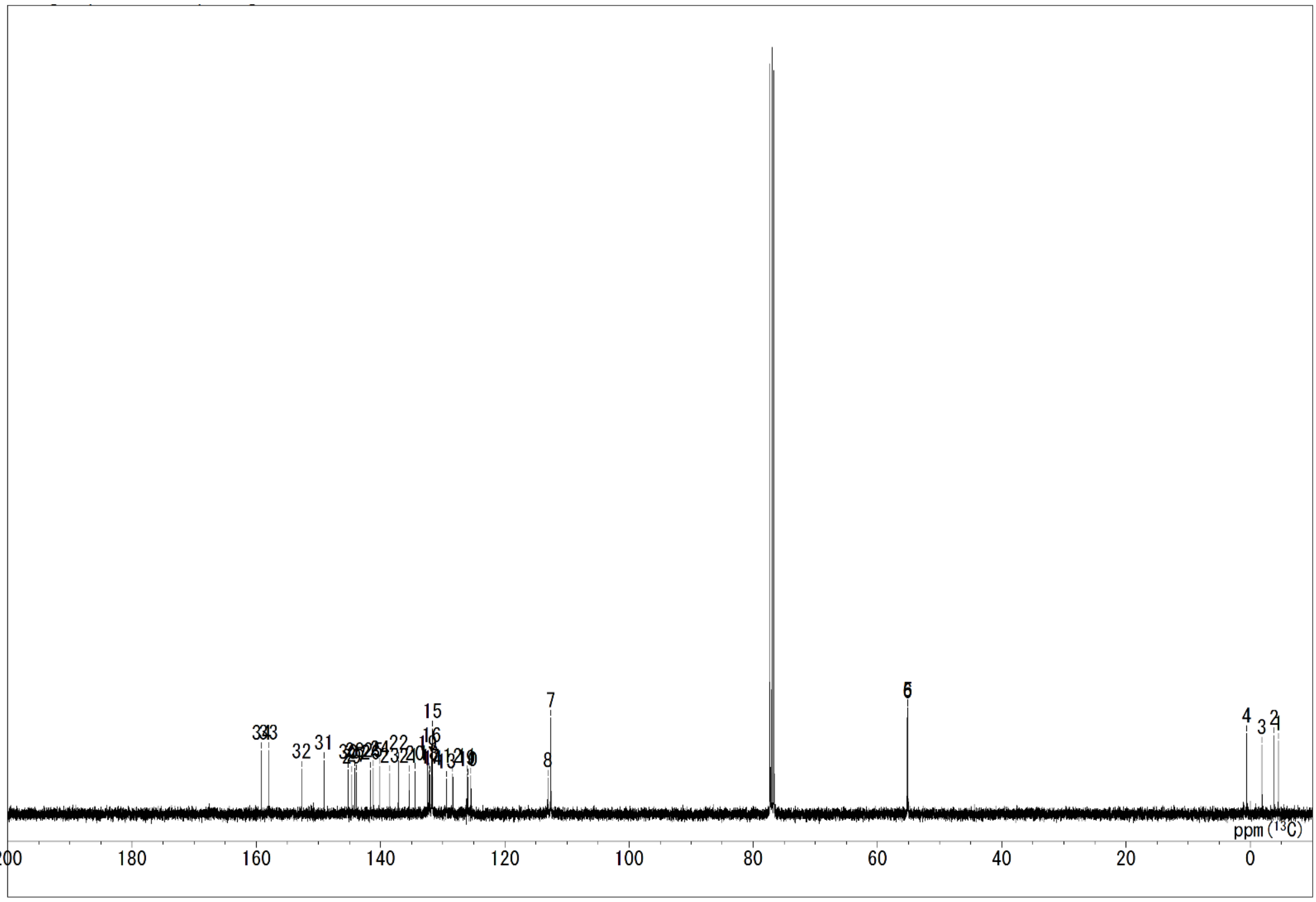

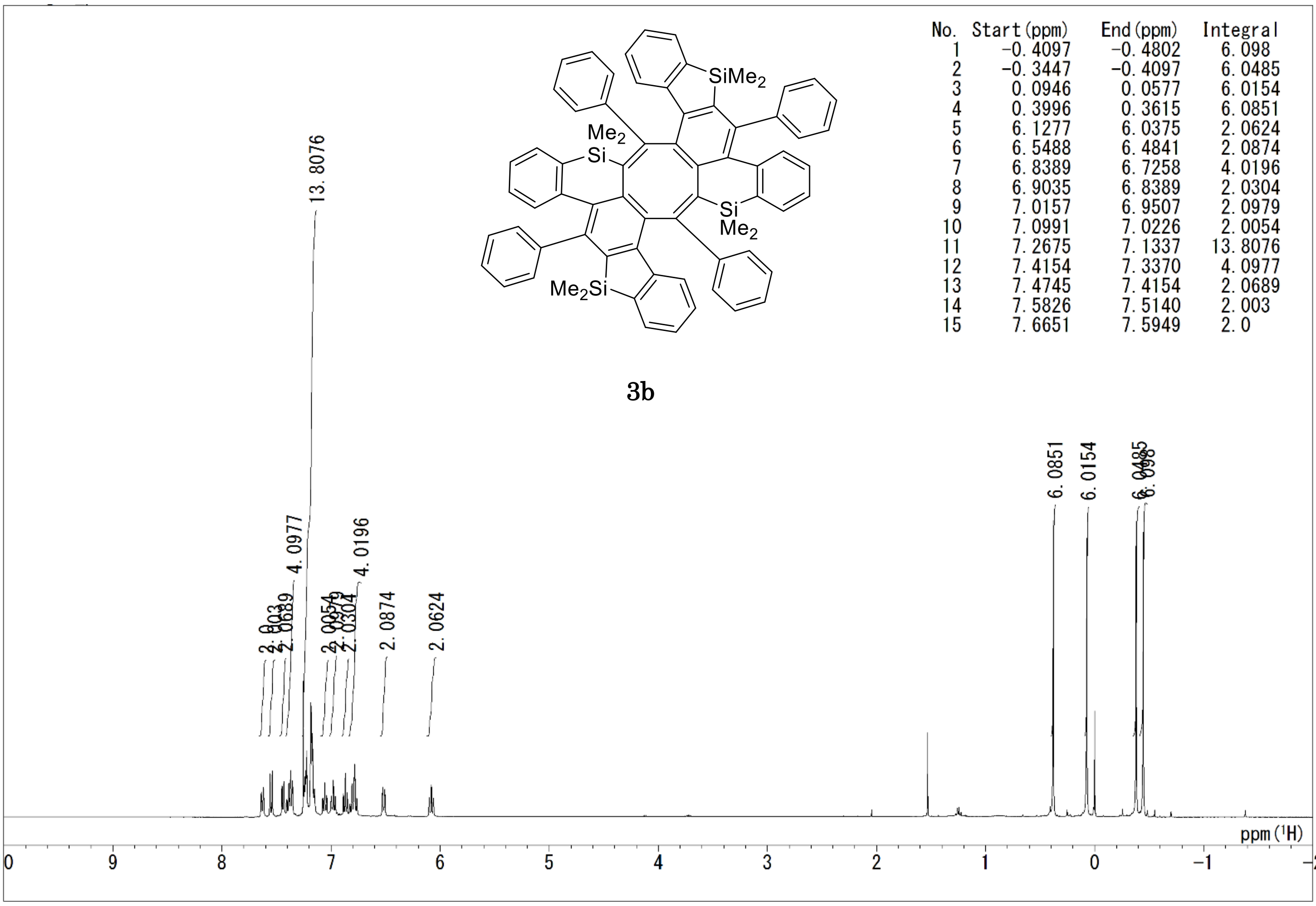
SiMe$_2$
Me$_2$
Si
Si
Me$_2$
Me$_2$Si
3b
No. Start(ppm) End(ppm) Integral
1 −0.4097 −0.4802 6.098
2 −0.3447 −0.4097 6.0485
3 0.0946 0.0577 6.0154
4 0.3996 0.3615 6.0851
5 6.1277 6.0375 2.0624
6 6.5488 6.4841 2.0874
7 6.8389 6.7258 4.0196
8 6.9035 6.8389 2.0304
9 7.0157 6.9507 2.0979
10 7.0991 7.0226 2.0054
11 7.2675 7.1337 13.8076
12 7.4154 7.3370 4.0977
13 7.4745 7.4154 2.0689
14 7.5826 7.5140 2.003
15 7.6651 7.5949 2.0
ppm (^{1}H)

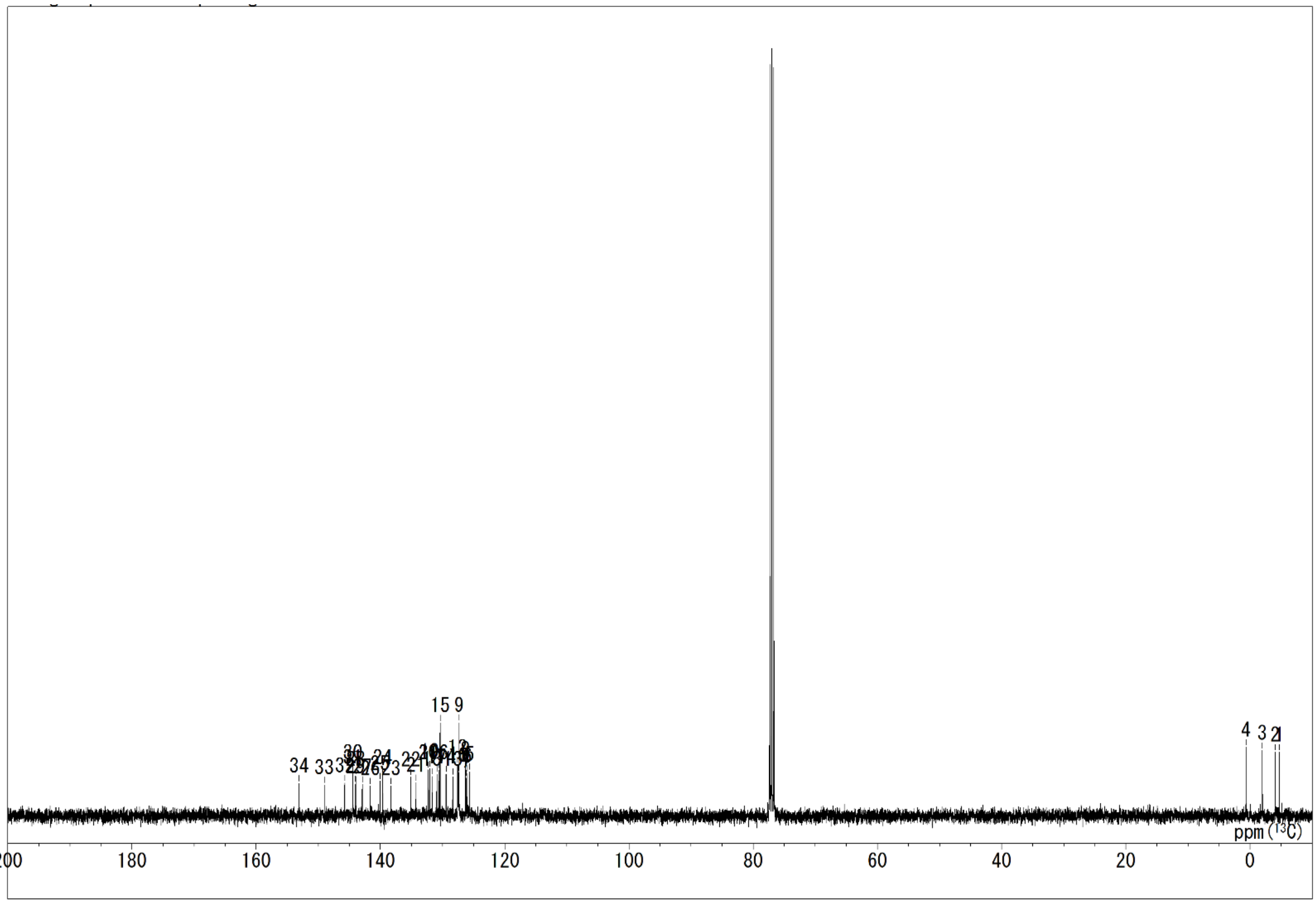
ppm (^{13}C)

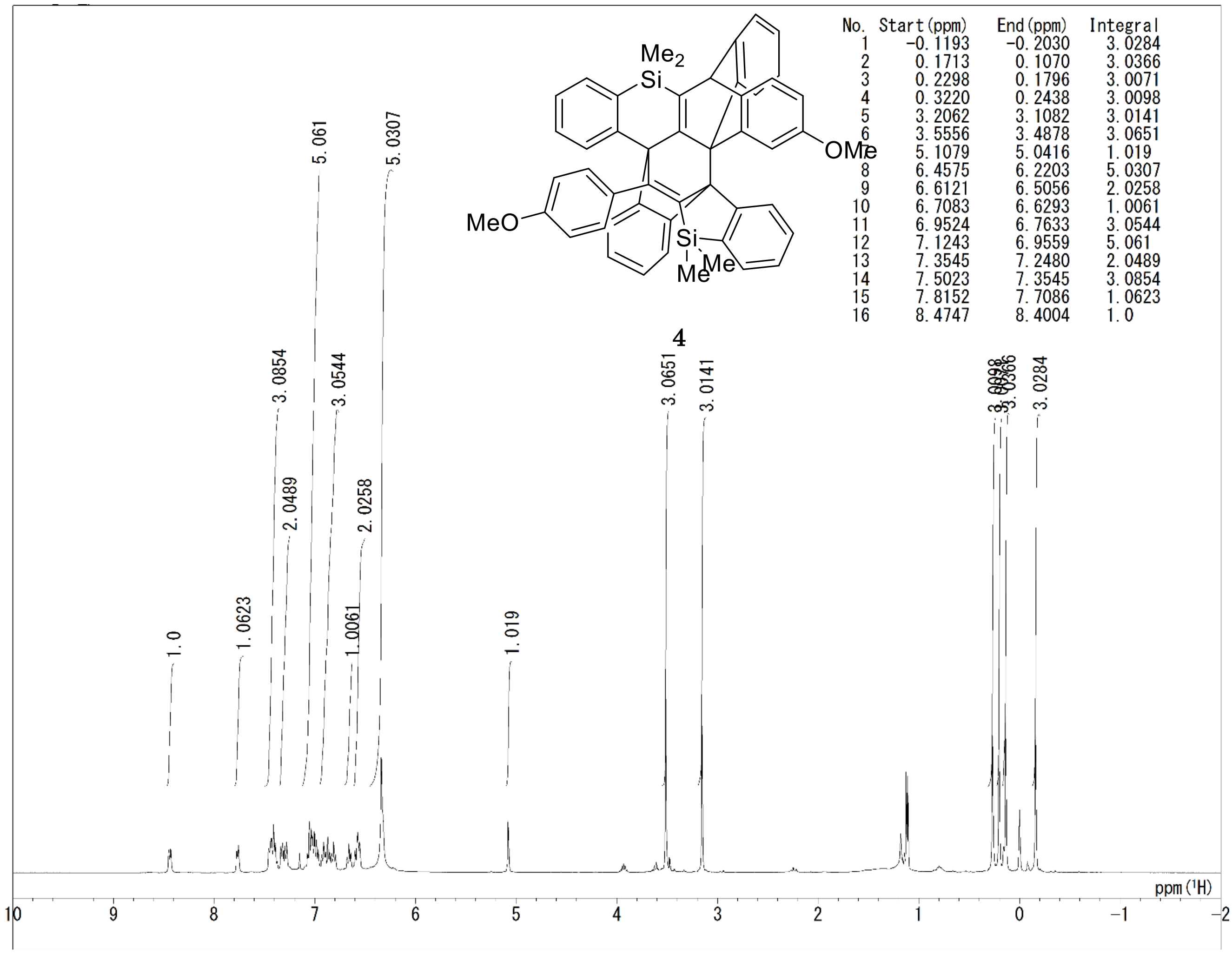

No.	Start (ppm)	End (ppm)	Integral
1	−0.1193	−0.2030	3.0284
2	0.1713	0.1070	3.0366
3	0.2298	0.1796	3.0071
4	0.3220	0.2438	3.0098
5	3.2062	3.1082	3.0141
6	3.5556	3.4878	3.0651
7	5.1079	5.0416	1.019
8	6.4575	6.2203	5.0307
9	6.6121	6.5056	2.0258
10	6.7083	6.6293	1.0061
11	6.9524	6.7633	3.0544
12	7.1243	6.9559	5.061
13	7.3545	7.2480	2.0489
14	7.5023	7.3545	3.0854
15	7.8152	7.7086	1.0623
16	8.4747	8.4004	1.0

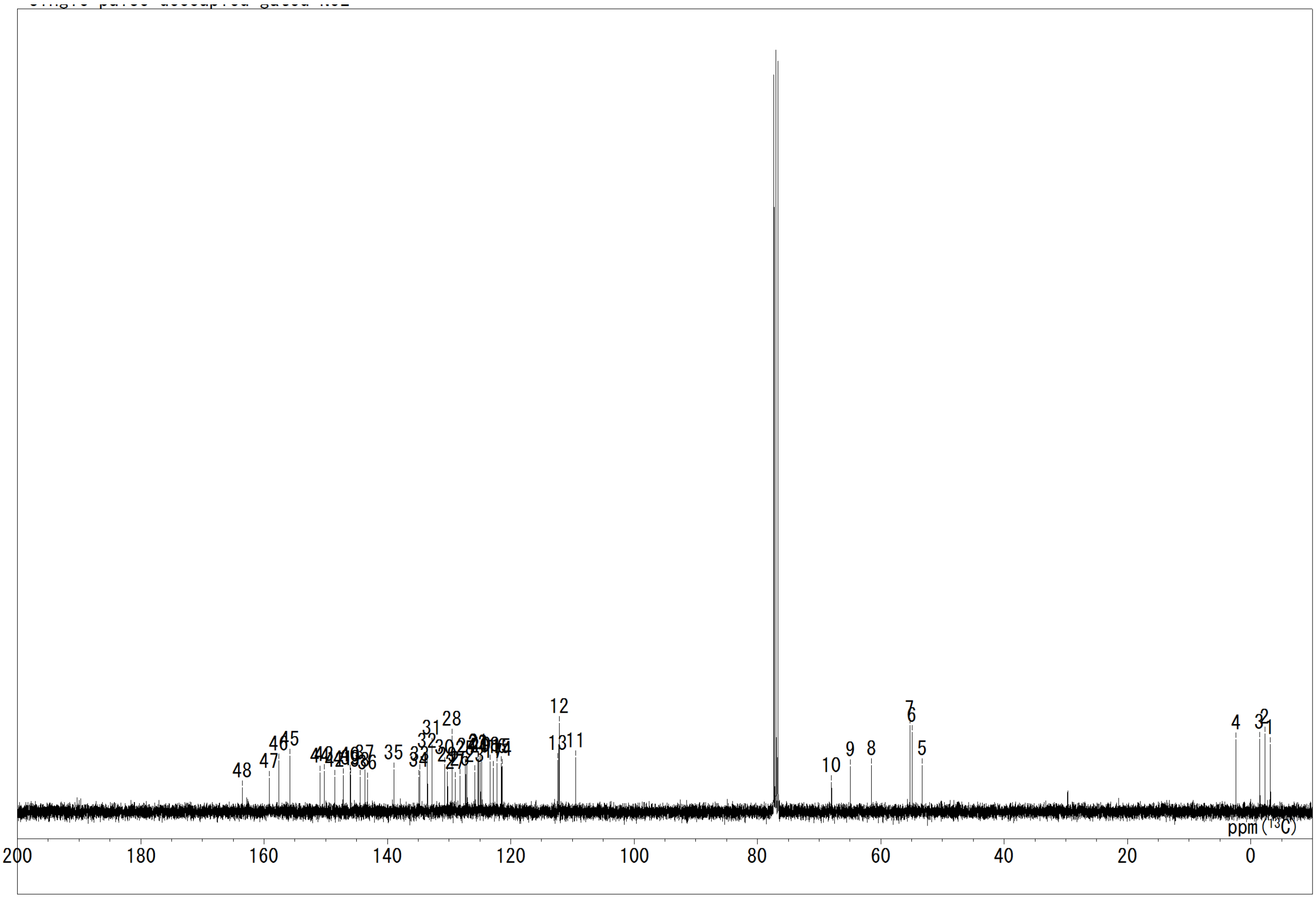

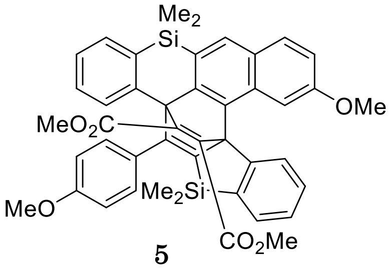

5

No.	Start (ppm)	End (ppm)	Integral
1	-0.4012	-0.4506	3.0038
2	0.0266	-0.0203	3.069
3	0.4867	0.4373	3.084
4	0.6425	0.5931	3.0814
5	3.2413	3.2141	3.0956
6	3.4799	3.4511	3.0001
7	3.6288	3.6016	3.0866
8	3.7775	3.7344	3.0839
9	6.7020	6.6450	2.0604
10	7.1078	7.0406	3.0585
11	7.5437	7.3542	7.0018
12	7.7315	7.6795	1.0046
13	7.7851	7.7315	1.019
14	8.0149	7.9785	1.0065
15	8.0982	8.0480	1.0

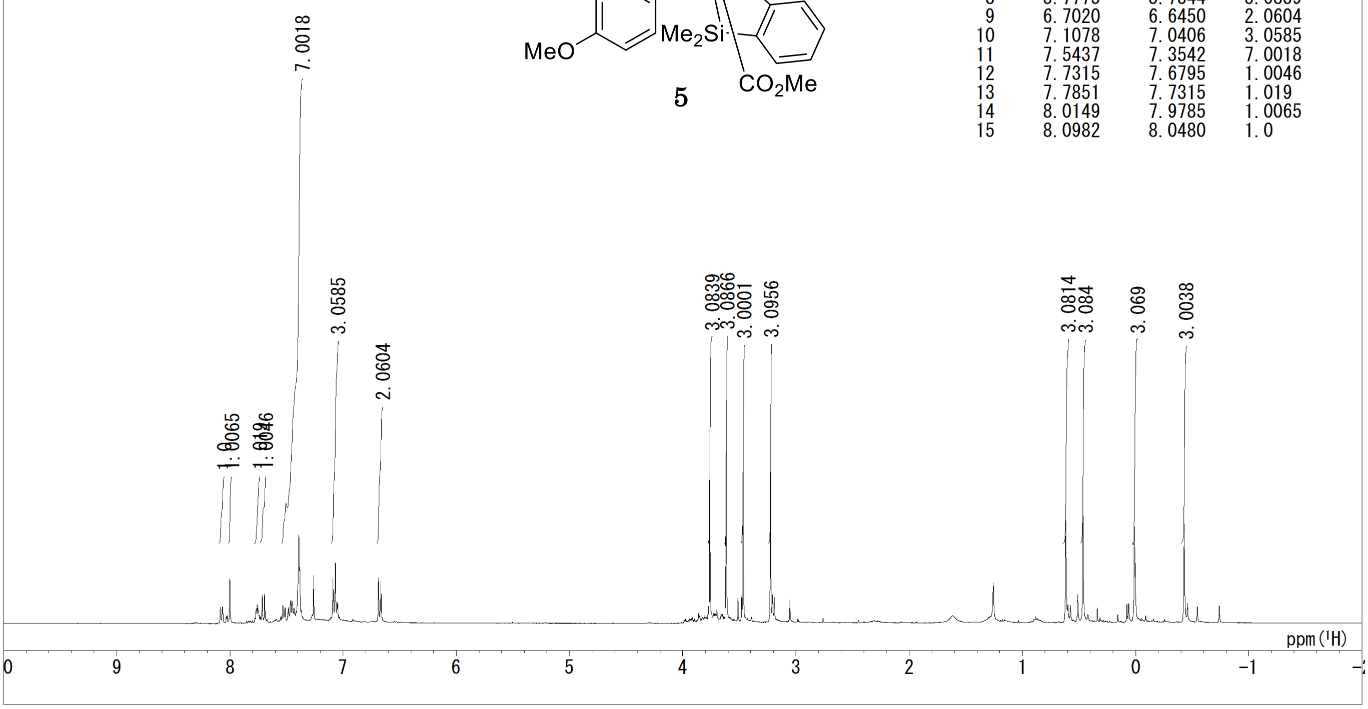

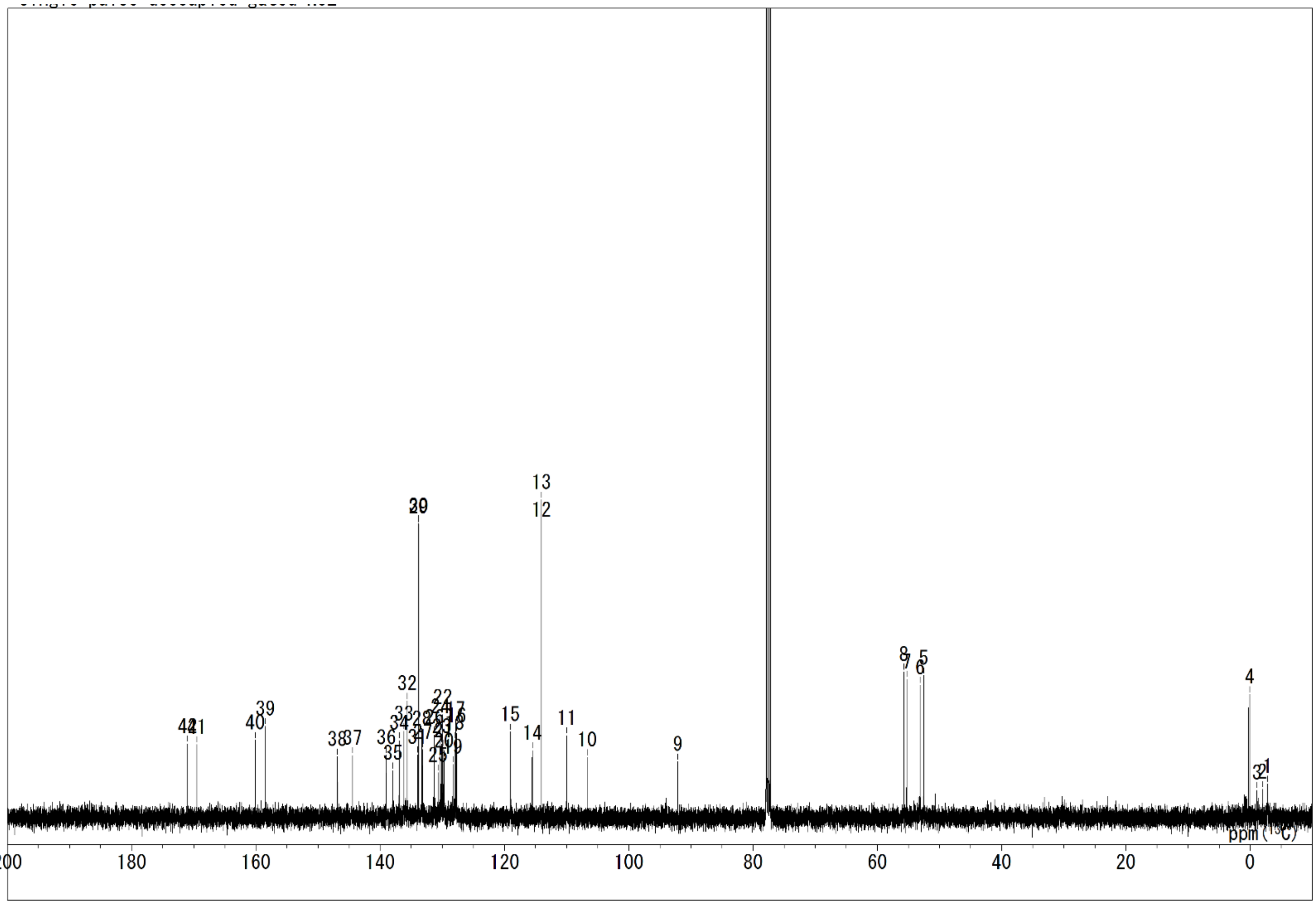

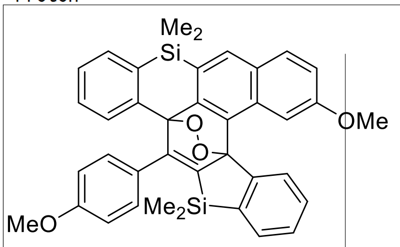

6

No.	Start (ppm)	End (ppm)	Integral	No.	Start (ppm)	End (ppm)	Inte
1	-0.0921	-0.1377	3.0	11	7.1462	7.0865	1.
2	0.5730	0.5372	2.9005	12	7.2605	7.2575	1.
3	0.6383	0.6063	2.89	13	7.3906	7.3450	1.
4	0.6814	0.6404	2.9561	14	7.5702	7.5220	1.
5	3.2402	3.1438	3.0701	15	7.6445	7.5826	2.
6	3.7406	3.6578	3.152	16	7.7507	7.6969	1.
7	6.3582	6.3083	1.0022	17	7.8062	7.7669	1.
8	6.6372	6.5438	2.0451	18	7.8714	7.8296	1.
9	6.8156	6.7426	2.0025	19	8.0399	8.0011	1.
10	6.9982	6.9436	1.0817				

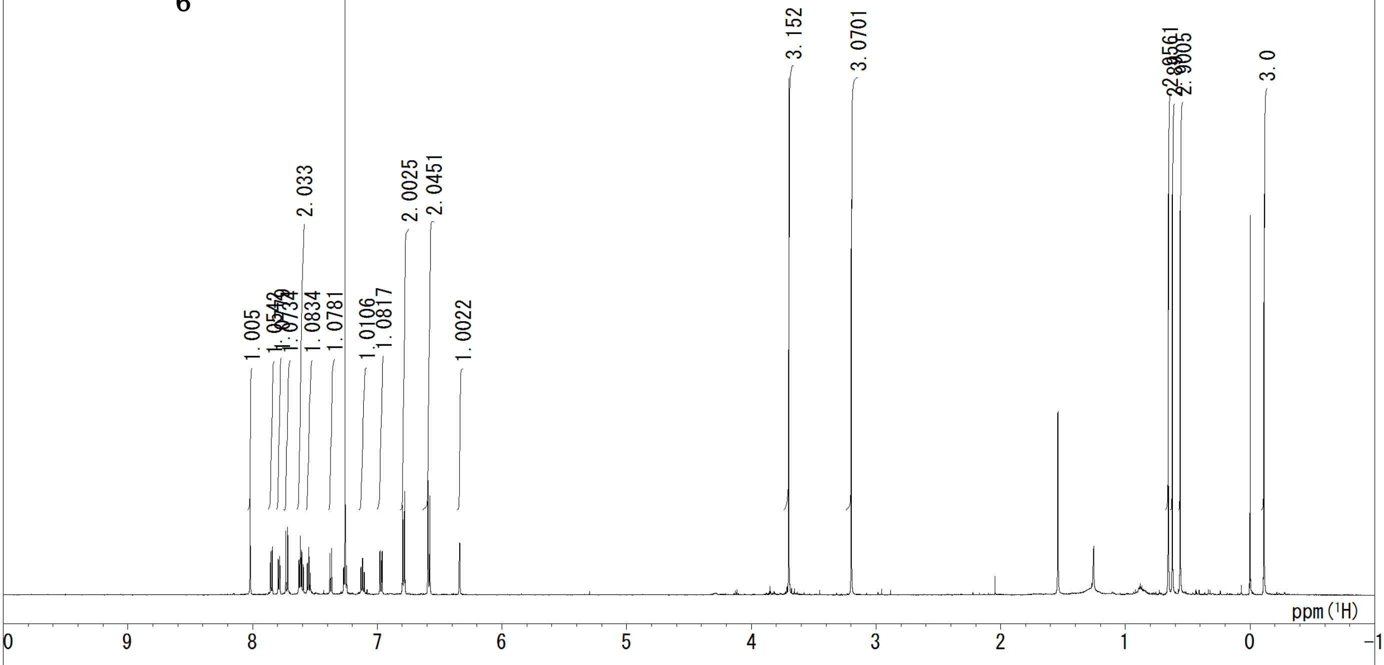

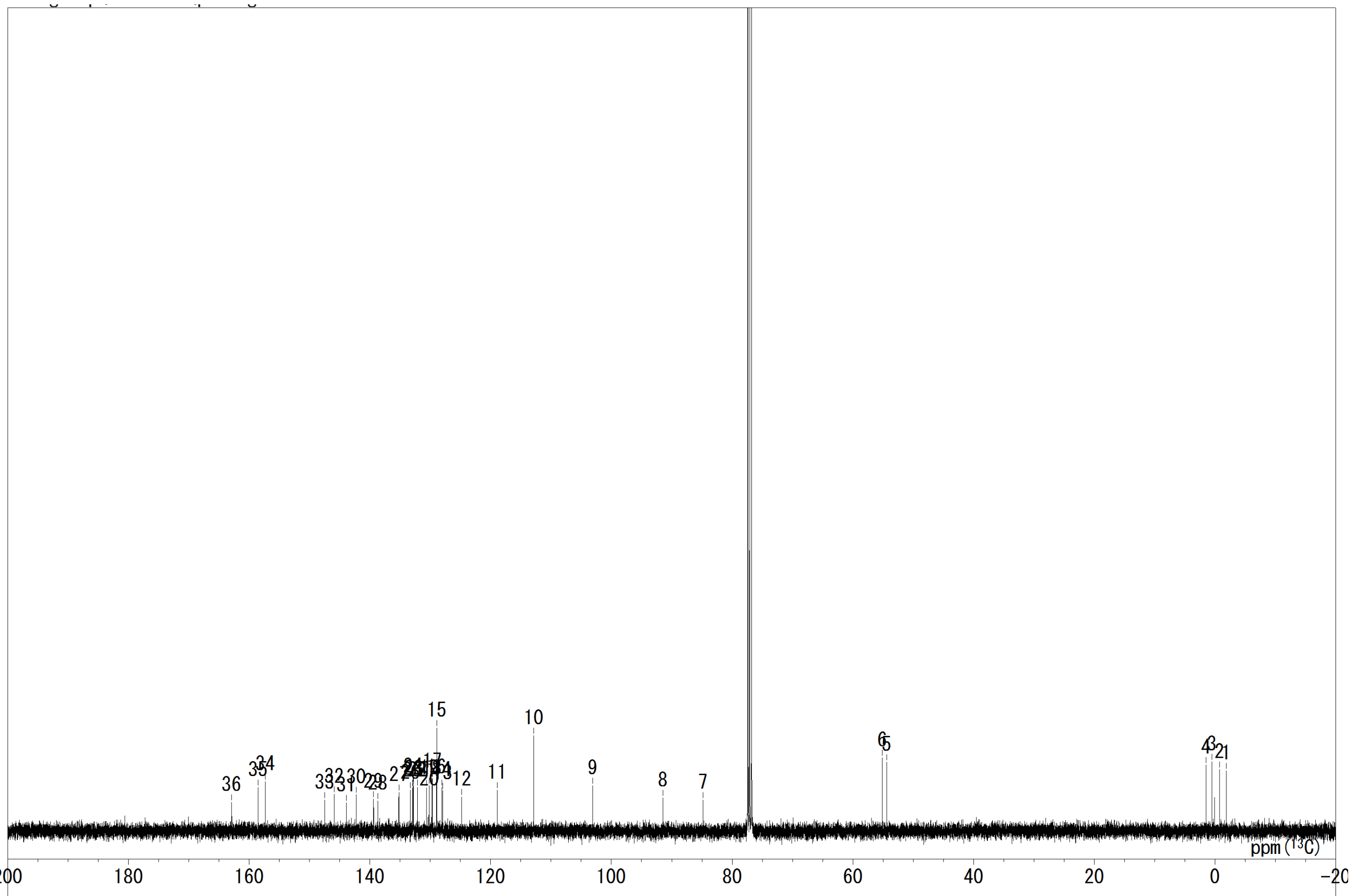

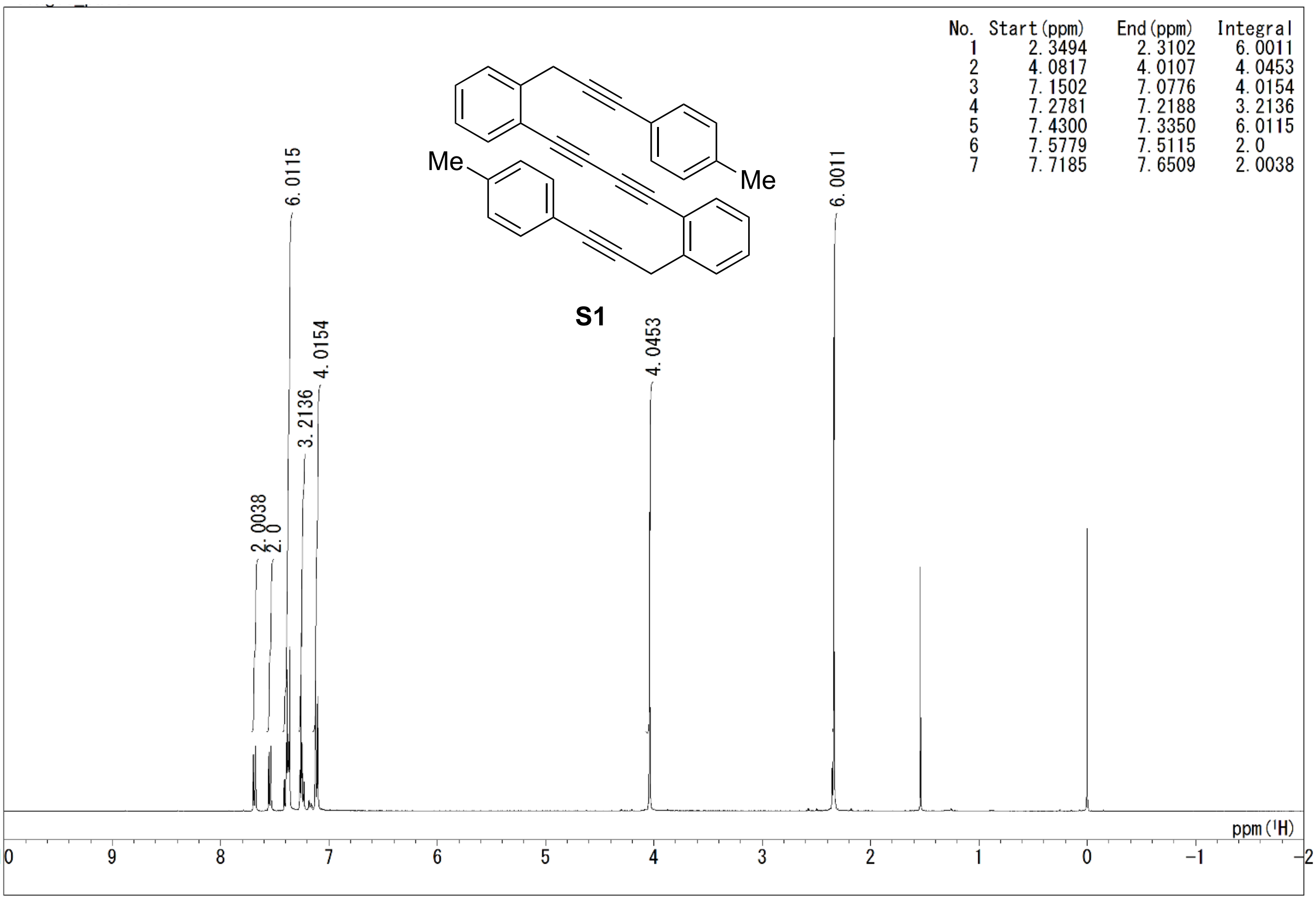

No. Start(ppm) End(ppm) Integral
1 2.3494 2.3102 6.0011
2 4.0817 4.0107 4.0453
3 7.1502 7.0776 4.0154
4 7.2781 7.2188 3.2136
5 7.4300 7.3350 6.0115
6 7.5779 7.5115 2.0
7 7.7185 7.6509 2.0038
Me
Me
S1
6.0115
4.0154
3.2136
2.0038
2.0
4.0453
6.0011
ppm (¹H)
10 9 8 7 6 5 4 3 2 1 0 −1 −2

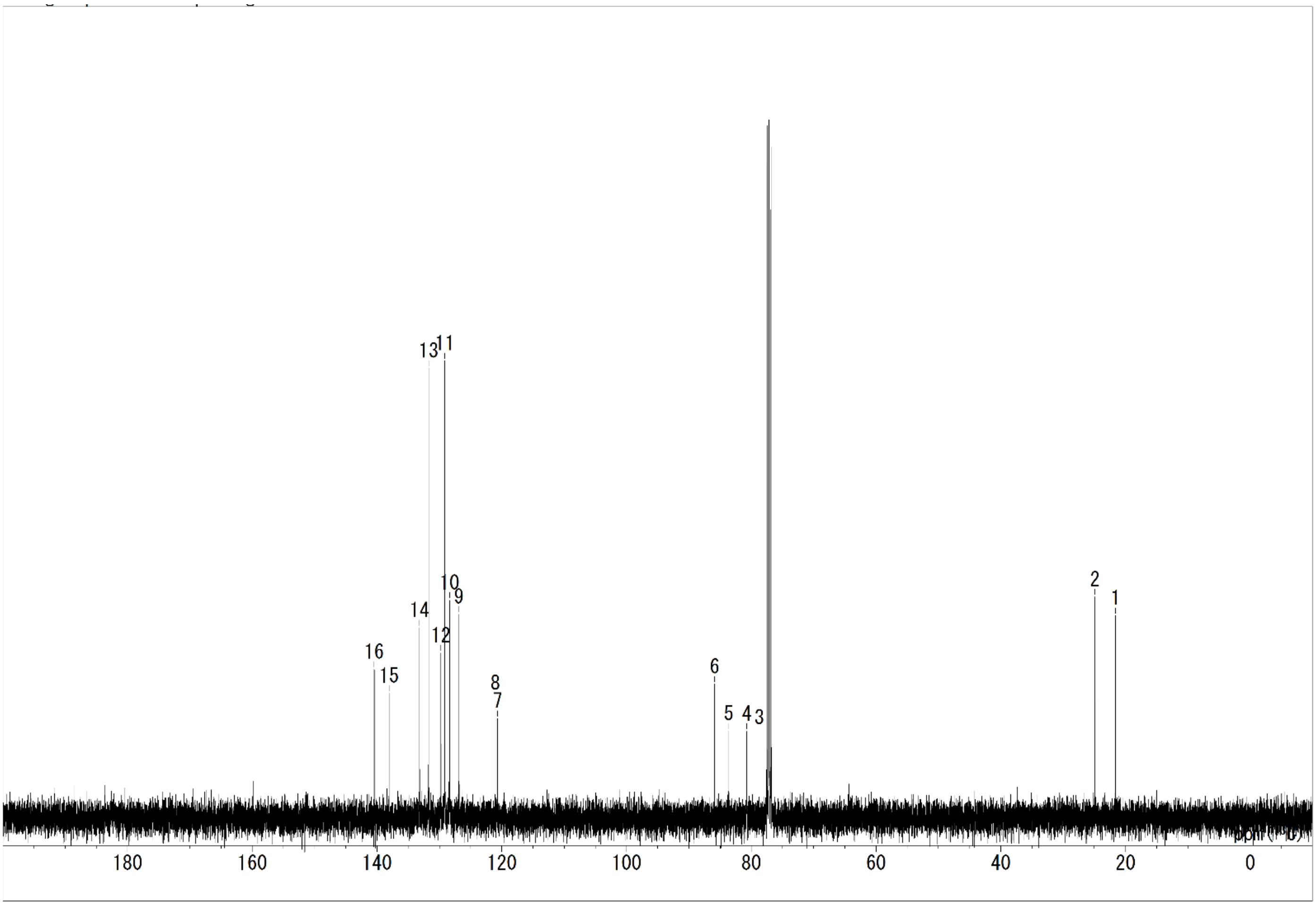

13 11
10 9
14
12
16 15
8 7
6
5 4 3
2 1
180 160 140 120 100 80 60 40 20 0

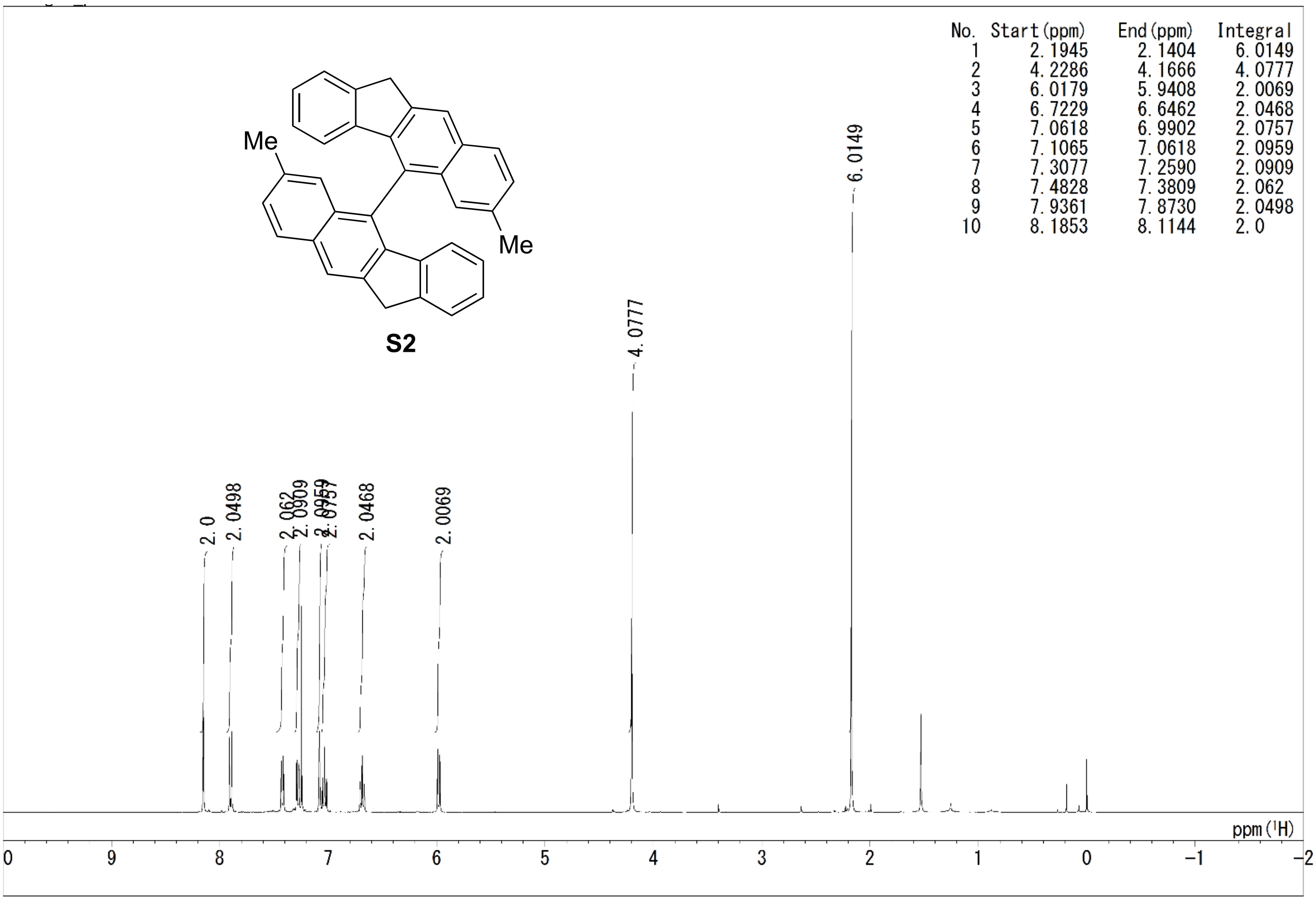
S2
Me
Me
No. Start(ppm) End(ppm) Integral
1 2.1945 2.1404 6.0149
2 4.2286 4.1666 4.0777
3 6.0179 5.9408 2.0069
4 6.7229 6.6462 2.0468
5 7.0618 6.9902 2.0757
6 7.1065 7.0618 2.0959
7 7.3077 7.2590 2.0909
8 7.4828 7.3809 2.062
9 7.9361 7.8730 2.0498
10 8.1853 8.1144 2.0
ppm (¹H)

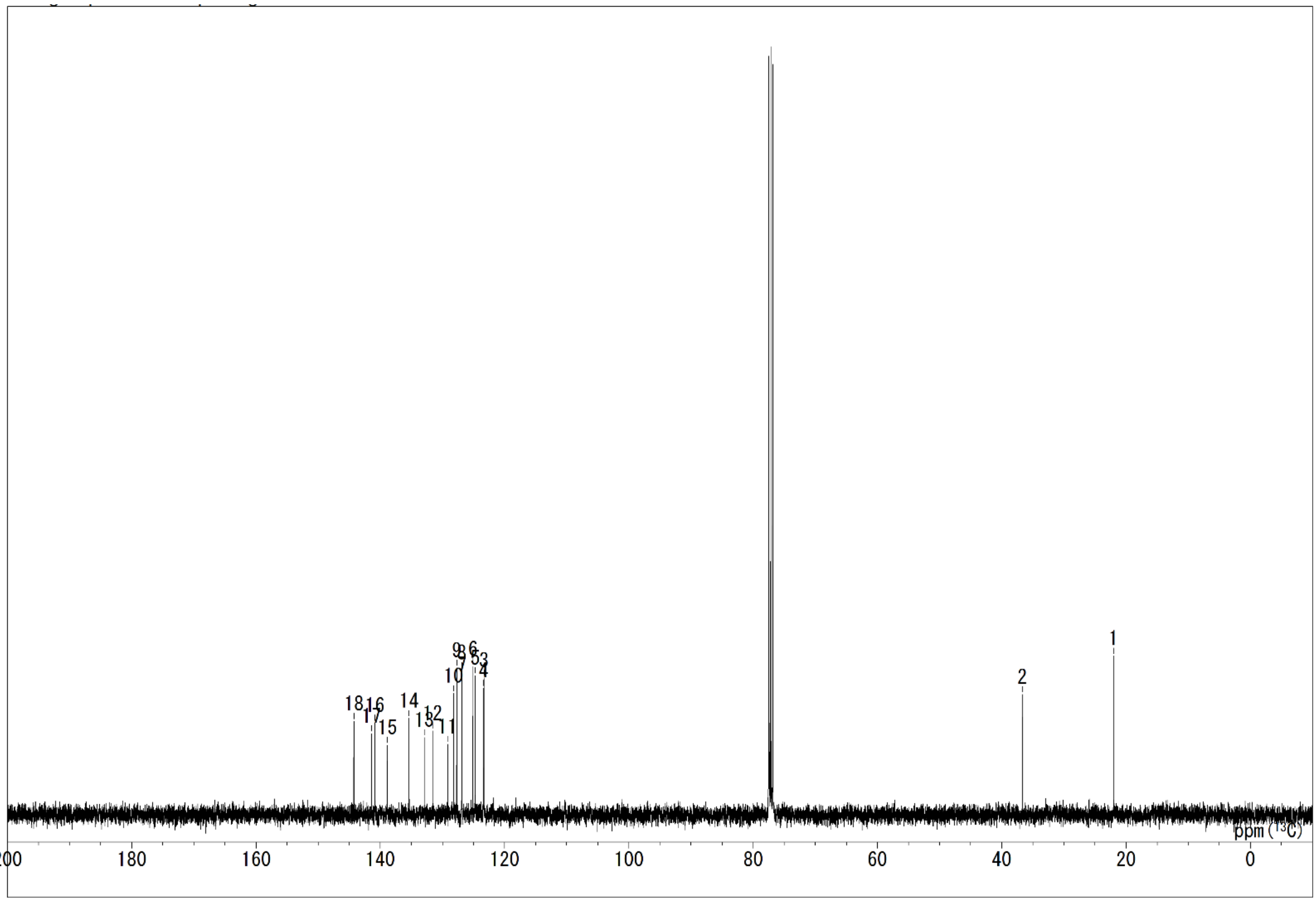
ppm (¹³C)
